# Nitric oxide responses in Arabidopsis hypocotyls are mediated by diverse phytohormone pathways

**DOI:** 10.1093/jxb/ery286

**Published:** 2018-08-02

**Authors:** Mari-Cruz Castillo, Alberto Coego, Álvaro Costa-Broseta, José León

**Affiliations:** Instituto de Biología Molecular y Celular de Plantas (Consejo Superior de Investigaciones Científicas–Universidad Politécnica de Valencia), Valencia, Spain

**Keywords:** ABA, *Arabidopsis thaliana*, brassinosteroids, ethylene, nitric oxide, salicylate, strigolactones, transcription factors, TRANSPLANTA lines

## Abstract

Plants are often exposed to high levels of nitric oxide (NO) that affects development and stress-triggered responses. However, the way in which plants sense NO is still largely unknown. Here we combine the analysis of early changes in the transcriptome of plants exposed to a short acute pulse of exogenous NO with the identification of transcription factors (TFs) involved in NO sensing. The NO-responsive transcriptome was enriched in hormone homeostasis- and signaling-related genes. To assess events involved in NO sensing in hypocotyls, we used a functional sensing assay based on the NO-induced inhibition of hypocotyl elongation in etiolated seedlings. Hormone-related mutants and the TRANSPLANTA collection of transgenic lines conditionally expressing Arabidopsis TFs were screened for NO-triggered hypocotyl shortening. These approaches allowed the identification of hormone-related TFs, ethylene perception and signaling, strigolactone biosynthesis and signaling, and salicylate production and accumulation that are essential for or modulate hypocotyl NO sensing. Moreover, NO inhibits hypocotyl elongation through the positive and negative regulation of some abscisic acid (ABA) receptors and transcripts encoding brassinosteroid signaling components thereby also implicating these hormones in NO sensing.

## Teaser

An NO-sensing mechanism controlling hypocotyl growth in etiolated seedlings requires the biosynthesis and/or signaling of ethylene, strigolactones, salicylate, abscisic acid, and brassinosteroids.

## Introduction

Nitric oxide (NO) is endogenously produced in diverse living organisms and regulates a wide array of biological processes. NO regulates plant developmental processes such as seed germination (Arc *et al.*, 2013), photomorphogenesis (Beligni and Lamattina, 2000; Lozano-Juste and León, 2011), flowering (He *et al.*, 2004; Tsai *et al.*, 2007), fruit ripening (Manjunatha *et al.*, 2010, 2012), or leaf senescence (Liu and Guo, 2013; Du *et al.*, 2014). NO is also a key regulatory molecule in the response of plants to environmental stress factors (Siddiqui *et al.*, 2011; Arasimowicz-Jelonek and Floryszak-Wieczorek, 2014). The regulatory mode of action of NO is probably based on its chemical nature as a free radical. It reacts mainly with metals and other free radicals in the microenvironment where it is produced (Thomas, 2015). In plants, NO is endogenously produced through stress-inducible oxidative and reductive biosynthetic pathways, although recent studies suggest that nitrite reduction is the main source (recently reviewed in Astier *et al.*, 2018). The oxidative status generated by reactive oxygen species in stressed plants seems to be alleviated by NO through the improvement of the antioxidant capacity, thus contributing to redox homeostasis (Correa-Aragunde *et al.*, 2015). However, extensive evidence suggests that NO is involved in somehow paradoxical processes exerting sometimes opposing regulatory functions. It has been reported that NO can enhance or reduce the redox status of the plants depending on acting in either a chronic or acute mode (Groß *et al.*, 2013). This paradox might be due to multiple factors including the relative NO cellular concentration, the location where it is produced, and the complex interacting microenvironment.

If the biosynthesis of NO still remains controversial, the way plants sense NO is even less known. NO perception in animals is accomplished through NO-inducible guanylate cyclases (GCs) that synthesize the second messenger cGMP from GTP (Friebe and Koesling, 2003; Russwurm and Koesling, 2004). Although a flavin monoxygenase called NO-dependent guanylate cyclase 1 (NOGC1), with higher affinity for NO than for molecular oxygen, was identified in Arabidopsis (Mulaudzi *et al.*, 2011), it remains controversial whether this enzyme produces enough cGMP to work as a true NO receptor (Gross and Durner, 2016). Moreover, it is also still unknown whether enzymes involved in cGMP degradation and downstream signaling, such as phosphodiesterases, are functional in plants (Gross and Durner, 2016), thus casting some doubt on the functionality of an NO–cGMP signaling pathway in plants. In the absence of an unequivocal GC receptor for NO in plants, alternative NO sensing mechanisms were searched for. We recently reported a mechanism for NO sensing, involving the specific oxidation of the C2 residue of transcription factors (TFs) of group VII of the ERF/AP2 family (herein after ERFVIIs), which is strictly dependent on NO and molecular oxygen, and allows further arginylation, polyubiquitylation, and proteasome-mediated degradation of ERFVIIs through the N-end rule proteolytic pathway (Gibbs *et al.*, 2014). Despite the relevance of ERFVIIs in sensing NO, other as yet not deciphered components should probably be involved in mediating NO sensitivity and responsiveness. This work deals with the identification of components mediating the plant sensitivity and responsiveness to NO. A simple sensitivity assay based on NO-triggered inhibition of hypocotyl elongation in etiolated seedlings in combination with a non-targeted approach of identification of the NO-responsive transcriptome allowed us to find a tight functional relationship between NO-triggered responses and regulation exerted through several hormone biosynthesis and signaling pathways. This signaling network includes ethylene, strigolactones (SLs), and salicylates as main regulatory factors, and abscisic acid (ABA) and brassinosteroids (BRs) as additional modulators in NO sensing.

## Materials and methods

### Plant materials, growth conditions, and NO treatment

Wild-type Col-0 seeds of *Arabidopsis thaliana* were sown in moistened soil and grown under photoperiod cycles of 16 h day and 8 h night (long days, at 22 °C and 20 °C, respectively), under 150 μE m^−2^ s^−1^ cool-white fluorescent lamps and 60% relative humidity. Alternatively, surface-sterilized seeds were sown after 4 d of stratification at 4 °C under darkness and grown in agar-supplemented Murashige and Skoog (MS) medium (Duchefa, Haarlem, The Netherlands) supplemented with 1% (w/v) sucrose.

The pulse of NO was performed by incubating plants for 5 min in a tightly sealed transparent box after injection of 300 ppm of pure NO gas (Linde AG, Germany).

### Assay for NO-triggered inhibition of hypocotyl elongation

Surface-sterilized seeds were sown in MS–MES media supplemented with 1% sucrose, stratified for 4 d at 4 °C under darkness, the germination program was activated by exposure to light for 6 h, and then they were incubated in tightly sealed boxes under air supplemented with 300 ppm pure NO gas under darkness for an additional 4 d. Control seedlings were incubated under the same conditions in air with no supplemented NO. The screenings of TPT transgenic lines (Coego *et al.*, 2014) were performed by using MS–MES media supplemented or not with 10 μM β-estradiol and treated or not with NO. Hypocotyl length was measured for every seedling of each genotype and condition tested by using Image J. The experiments were repeated three times with at least 20 individuals per genotype, condition, and experiment.

### RNA isolation, real-time quantitative PCR (RT-qPCR), and transcriptomic analyses

Plants grown *in vitro* under long-day (16 h light/8 h darkness) conditions for 12 d, or under skotomorphogenic conditions under darkness for 4 d, as indicated, were exposed to a pulse of NO (300 ppm, 5 min). At the indicated times, total RNA was extracted and purified with the Nucleospin RNA Plant kit (Macherey-Nagel), reverse transcribed with M-MuLV Reverse transcriptase (RNase H minus) and oligo(dT), and the resulting cDNAs quantified by real-time PCR with ABI 7500 Fast Real-Time Thermocyclers by using specific primer pairs (Supplementary Table S1 at *JXB* online). For microarray analyses, seedlings at 0, 15 min, 30 min, and 1 h after NO pulse were frozen in liquid nitrogen and the total RNAs were extracted with Trizol and purified with the RNeasy kit (QIAGEN). RNAs (three independent biological replicates per genotype) were checked for their integrity and purity by nanocapillary electrophoresis in a Bioanalyzer Agilent 2100. The transcriptomes were analyzed by using the Arabidopsis Nimblegen-Roche microarray platform. Labeling, hybridization protocols, and statistical analyses are included in a detailed MIAME rules-based description of the microarray experiments in Supplementary Table S2.

### Statistical analyses

Differential gene transcript levels or hypocotyl lengths were statistically analyzed by Student’s *t*-test and considered significant with a *P*-value ≤0.05. Linear model methods (LiMMA) were used for determining differentially expressed genes in microarray-based analyses. To control the false discovery rate (FDR), *P*-values were corrected using the method of Benjamini and Hochberg (1995). Criteria for selection of genes were fold value >1.5 and FDR ≤0.05. Statistical analysis and graphical visualization of data were performed with the interactive tool FIESTA (http://bioinfogp.cnb.csic.es/tools/FIESTA/).

Comparison of transcriptome data sets was performed with AtCAST3.1 (http://atpbsmd.yokohama-cu.ac.jp/) by selecting data from different ATH1 experiments with a Student’s *t*-test *P*-value of <0.01. Spearman’s rank order correlation coefficients (SCCs) were used to estimate the functional overlap/co-expression between experiments (Kakei and Shimada, 2015).

### *In silico* analyses of Gene Ontology and transcriptome profiles

Gene Ontology enrichment of functional categories in gene lists was performed by the Gene Ontology Consortium tools (http://www.geneontology.org/). Comparison of transcriptome profiles with publicly available data sets was performed with the AtCAST3.1 tool (http://atpbsmd.yokohama-cu.ac.jp/cgi/atcast/search_input.cgi).

### Prediction of *S*-nitrosylation and nitration sites

*S*-nitrosylation and nitration sites on potential NO target proteins were predicted by GPS-SNO (Xue *et al.*, 2010; http://sno.biocuckoo.org/) and iSNOPseAAC (Xu *et al.*, 2013; http://app.aporc.org/iSNO-PseAAC/); and GPS-YNO2 (Liu *et al.*, 2011; http://yno2.biocuckoo.org/) and iNitro-Tyr (Xu, *et al.*, 2014; http://app.aporc.org/iNitro-Tyr/) tools.

## Results

### Over-representation of hormone- and oxygen-related regulatory components in the early NO-responsive transcriptome

**Fig. 1.**
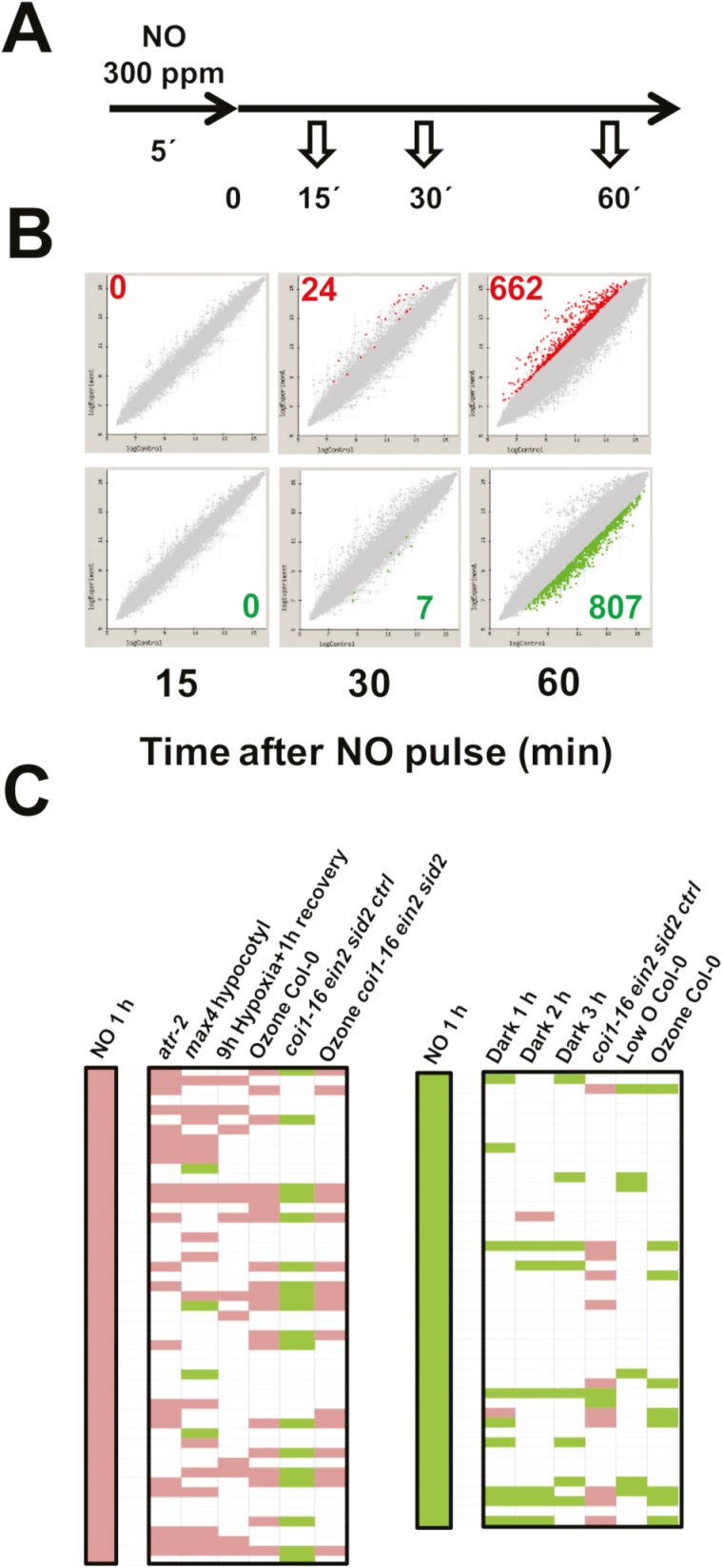
Identification and characterization of the NO-responsive transcriptome. (A) Experimental scheme of sampling. (B) Plots representing the up-regulated (dots in the upper panel) and down-regulated (dots in the lower panel) genes at the indicated times after exposure to NO. (C) AtCAST3.1-based comparison of the up- and down-regulated transcriptomes at 1 h after exposure to NO with publicly available hormone- and redox-related transcriptome data sets.

To unravel the sensing mechanism underlying the early plant responses to NO, we have designed an experimental system based on *A. thaliana* seedlings exposed to a short pulse of pure NO gas. We previously reported an increase in NO-related post-translational modification of proteins and an extensive metabolic re-arrangement by 1 h and 6 h after treatment, respectively (León *et al.*, 2016). These data suggested changes should occur in the time frame between a few minutes and 1 h after NO exposure. Thus, we analyzed changes in the Arabidopsis transcriptome shortly after exposure to an NO pulse for 5 min. Samples were harvested at 15, 30, and 60 min for subsequent transcriptome analyses (Fig. 1A). Overall analyses indicated that by 15 min after exposure to NO, no significant change was observed in any gene transcript when compared with those of untreated seedlings (Fig. 1B). By 30 min, 24 and 7 genes were significantly up- or down-regulated, respectively (Fig. 1B). After that, more extensive changes in transcript levels were detected by 60 min, with ~1500 genes differentially expressed, representing ~5% of the Arabidopsis genome. A total of 662 and 807 genes were up- and down-regulated, respectively (Fig. 1B). The complete list of differentially expressed genes including annotation, fold changes, and *P*-values corrected for FDRs, as well as a full MIAME-description of the transcriptome analyses is shown in Supplementary Table S2. A PANTHER over-representation test of Gene Ontology analysis, using the *A. thaliana* database of the Gene Ontology Consortium (http://www.geneontology.org/), with genes differentially expressed by 1 h after NO exposure allowing identification that the response to chitin, the responses to hormones, particularly to ethylene and jasmonates, as well as the responses to hypoxia functional categories were significantly over-represented (Supplementary Table S3). On the other hand, a comparison of the 50 top up-regulated NO-responsive genes identified here at 1 h after NO with publicly available transcriptome data by using AtCAST3.1 (Kakei and Shimada, 2015; http://atpbsmd.yokohama-cu.ac.jp/cgi/atcast/search_input.cgi) showed significant co-regulation profiles with those also up-regulated in *atr-2* (GEO code GSE63355) and *max4* (GEO code GSE6151) mutants, as well as with the re-oxygenated plants after hypoxia (Branco-Price *et al.*, 2008; GEO code GSE9719), or the ozone-treated wild-type and *coi1-16ein2sid2ctrl* (GEO code GSE65740) plants (Fig. 1C). Also a significant anti-regulation was observed for the NO-responsive transcriptome at 1 h with the transcriptome of the untreated *coi1-16ein2sid2ctrl* (GEO code GSE65740) mutant (Fig. 1C). Genes that were down-regulated by NO showed only a significant co-regulation with the transcriptome of plants under darkness (Fig. 1C).

### A sensing test based on inhibition of hypocotyl elongation allowed identification of hormone-related transcription factors modulating NO sensitivity

**Fig. 2.**
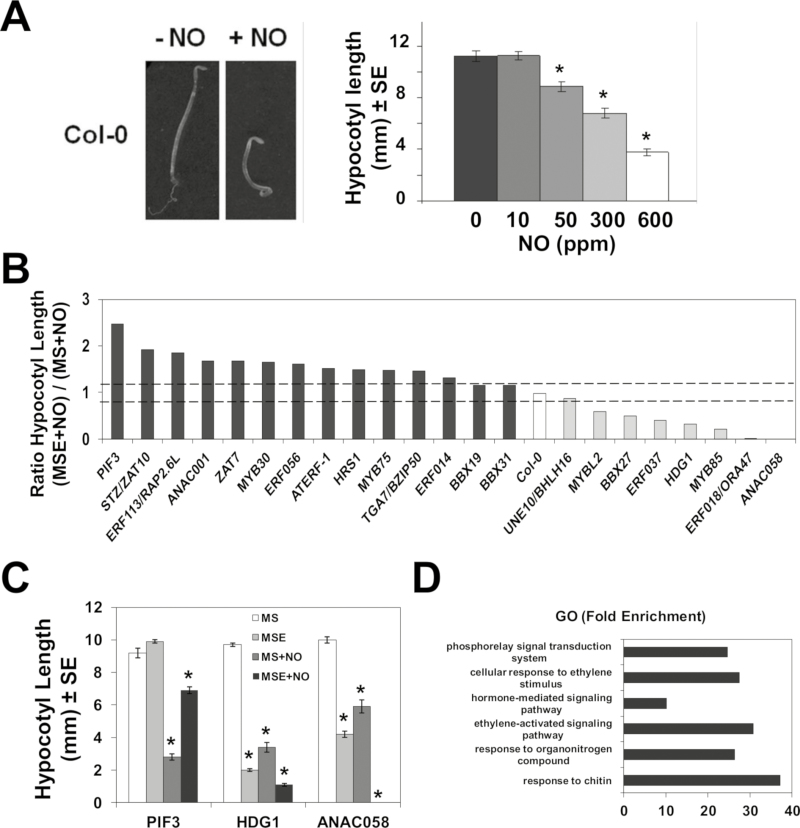
Screening of TPT transgenic lines conditionally expressing transcription factor-encoding genes through an NO-triggered hypocotyl shortening in etiolated seedlings. (A) NO triggers a dose-dependent hypocotyl shortening. (B) Ratio of the hypocotyl lengths in TPT lines exposed to NO in the absence (MS) or presence of the inducer β-estradiol (MSE). Dashed lines represent the upper and lower variability limits of the ratios of wild-type Col-0 hypocotyl lengths. (C) Hypocotyl lengths of TPT lines expressing PIF3, HDG1, and ANAC058 transcription factors are altered by β-estradiol treatment even in the absence of NO. (D) Gene Ontology (GO) analysis points to a significant enrichment of the hormone and organonitrogen compound categories among genes triggering significant hypo- or hypersensitivity to NO in the screening of TPT lines. *P-values <0.05 in Student’s *t*-test.

Several of the transcriptomes that display partial overlapping with the transcriptome of NO-exposed plants (Fig. 1) corresponded to experiments performed with hypocotyl samples, thus suggesting NO-exerted regulation could be relevant in hypocotyls. Etiolated plants exposed to exogenous NO are characterized by root growth arrest and hypocotyl shortening (Fig. 2A). The inhibition of hypocotyl elongation was proportional to NO concentrations, with a 50% inhibition after exposure to 300 ppm NO (Fig. 2A). We have used this simple and quantitative NO sensing test to screen 968 Arabidopsis TRANSPLANTA transgenic lines (Supplementary Table S4), conditionally expressing 263 TFs under a β-estradiol-inducible promoter (Coego *et al.*, 2014). Different levels of induced expression ranging from 5- to 250-fold were detected upon β-estradiol treatment of transgenic lines, as shown for a randomly selected group (Supplementary Fig. S1). Several independent transgenic lines for each TF were analyzed for hypocotyl length in etiolated untreated (MS) or β-estradiol-induced (MSE) seedlings, or those conditions plus NO treatment, MS+NO and MSE+NO, respectively. Our screen searched for TFs causing either attenuated inhibition (hyposensitivity) or enhanced inhibition (hypersensitivity) to NO upon conditional β-estradiol-induced expression. The β-estradiol-induced expression of some TFs such as ZAT10 and MYB85 attenuated and potentiated the NO-triggered inhibition of hypocotyl elongation, thus inducing NO hyposensitivity and hypersensitivity, respectively. The ratios between hypocotyl length of β-estradiol-treated and untreated NO-exposed seedlings determined whether the expression of a given TF brings about hyposensitivity or hypersensitivity to NO relative to wild-type Col-0 plants with ratios of ~1 and variability <12% (Fig. 2B). Table 1 shows the 56 lines corresponding to 22 TFs that showed β-estradiol-dependent hyposensitivity or hypersensitivity to NO. As proofs of concept in our screening, the TF inducing the strongest NO hyposensitivity was PIF3, which has already been reported to promote hypocotyl elongation (Feng *et al.*, 2008). Another TF identified, MYB30, has been reported to be functionally related to NO-triggered responses (Tavares *et al.*, 2014). Moreover, this sort of screening also allows identification of TFs that themselves regulate the elongation of the hypocotyl in the absence of NO treatment, such as HDG1 or ANAC058 that induced shortening, or the above-mentioned PIF3, which promoted elongation (Fig. 2C). Paralleling the effects in the absence of NO, those factors also caused hyposensitivity and hypersensitivity to NO, respectively (Fig. 2C). Gene Ontology analysis points to a significant over-representation of the functional categories of hormone signaling pathways, particularly ethylene, among the TFs listed in Table 1 (Fig. 2D). Among the identified TFs, we found the AP2-related and integrase-type ORA47 and RAP2.6L (Krishnaswamy *et al.*, 2011; Chen *et al.*, 2016), as well as four additional integrase-type ERF TFs, ERF014, ERF037, ERF056, and ERF113/RAP2.6L, and the ethylene-responsive element-binding factor 1 (ATERF-1), all of them related to ethylene signaling. Also TFs that are functionally related to ABA homeostasis or signaling such as the MYB-type HRS1 and MYB30 (Wu *et al.*, 2012; Lee and Seo, 2016) and the NAC058 TF (Coego *et al.*, 2014) were identified in the screening. The functional interaction between NO sensitivity and hormone signaling was not restricted to ABA and ethylene. Among TFs identified in the screening, ORA47 and ZAT10 have also been characterized as positive and negative regulators, respectively, of jasmonic acid (JA) signaling (Pauwels *et al.*, 2008), and MYB30 regulates BR signaling (Li *et al.*, 2009). Finally, PAP1/MYB75 and MYBL2 regulate the biosynthesis of anthocyanins and flavonoids, the latter functioning as a node between JA and gibberellin (GA) signaling (Xie *et al.*, 2016); and MYB75/PAP1 and MYB85 seems to be also involved in the secondary cell wall formation or thickening, and in the lignification of stems (Bhargava *et al.*, 2013; Zhong *et al.*, 2008), a process that tightly controls the growth of hypocotyls and other plant organs (Hamant and Traas 2010). These data, together with the Gene Ontology analyses (Supplementary Table S3) and the co-regulation of transcriptomes (Fig. 1) shown above, strongly suggest a determinant involvement of hormone biosynthesis and signaling in NO sensing.

**Table 1.** TPT lines showing hypo- or hypersensitivity to NO for hypocotyl elongation of etiolated seedlings upon conditional expression of TF-encoding genes

AGI code	Annotation	TPT lines	NO sensitivity
AT1G09530	PIF3_ phytochrome interacting factor 3	1.09530.1F4	Hyposensitive
AT4G17500	ATERF-1_ ethylene responsive element binding factor 1	4.17500.1B3, E2, I7	Hyposensitive
AT1G01010	ANAC001_ NAC domain containing protein 1	1.01010.1E5, F9, G3	Hyposensitive
AT1G13300	HRS1__myb-like transcription factor family protein	1.13300.1A9, D3, E5	Hyposensitive
AT1G56650	MYB75_ PAP1_ production of anthocyanin pigment 1	1.56650.1C5, H3	Hyposensitive
AT3G28910	MYB30_ myb domain protein 30	3.28910.1C1, E5	Hyposensitive
AT5G13330	Rap2.6L__related to AP2 6l	5.13330.1D9, G9, I4	Hyposensitive
AT2G22200	ERF056_Integrase-type DNA-binding superfamily protein	2.22200.1B1, E7, G8	Hyposensitive
AT1G44830	ERF014_Integrase-type DNA-binding superfamily protein	1.44830.1A99, F99, G99	Hyposensitive
AT3G46090	ZAT7__C2H2 and C2HC zinc fingers superfamily protein	3.46090.1B99,E99,H99,I99	Hyposensitive
AT1G27730	STZ_ZAT10__salt tolerance zinc finger	1.27730.1E5, I1	Hyposensitive
AT3G21890	BBX31_B-box type zinc finger family protein	3.21890.1A8, B5, H8	Hyposensitive
AT4G38960	BBX19_B-box type zinc finger family protein	4.38960.1A3, G3	Hyposensitive
AT1G77920	TGA7_bZIP transcription factor family protein	1.77920.1B3, E1, G3, H2	Hyposensitive
AT3G18400	ANAC058_ NAC domain containing protein 58	3.18400.1D3, G9	Hypersensitive
AT1G71030	MYBL2_ MYB-like 2	1.71030.1C5, H9	Hypersensitive
AT4G22680	MYB85_ myb domain protein 85	4.22680.1F1	Hypersensitive
AT1G77200	ERF037_Integrase-type DNA-binding superfamily protein	1.77200.1C99, H99	Hypersensitive
AT1G74930	ERF018/ORA47__Integrase-type DNA-binding protein	1.74930.1E2, F8, H4	Hypersensitive
AT2G30250	WRKY25_ WRKY DNA-binding protein 25	2.30250.1C5, D2, F2	Hypersensitive
AT1G68190	BBX27_B-box zinc finger family protein	1.68190.1D1	Hypersensitive

### Ethylene perception and signaling as well as salicylate and strigolactone biosynthesis are required for NO sensing

**Fig. 3.**
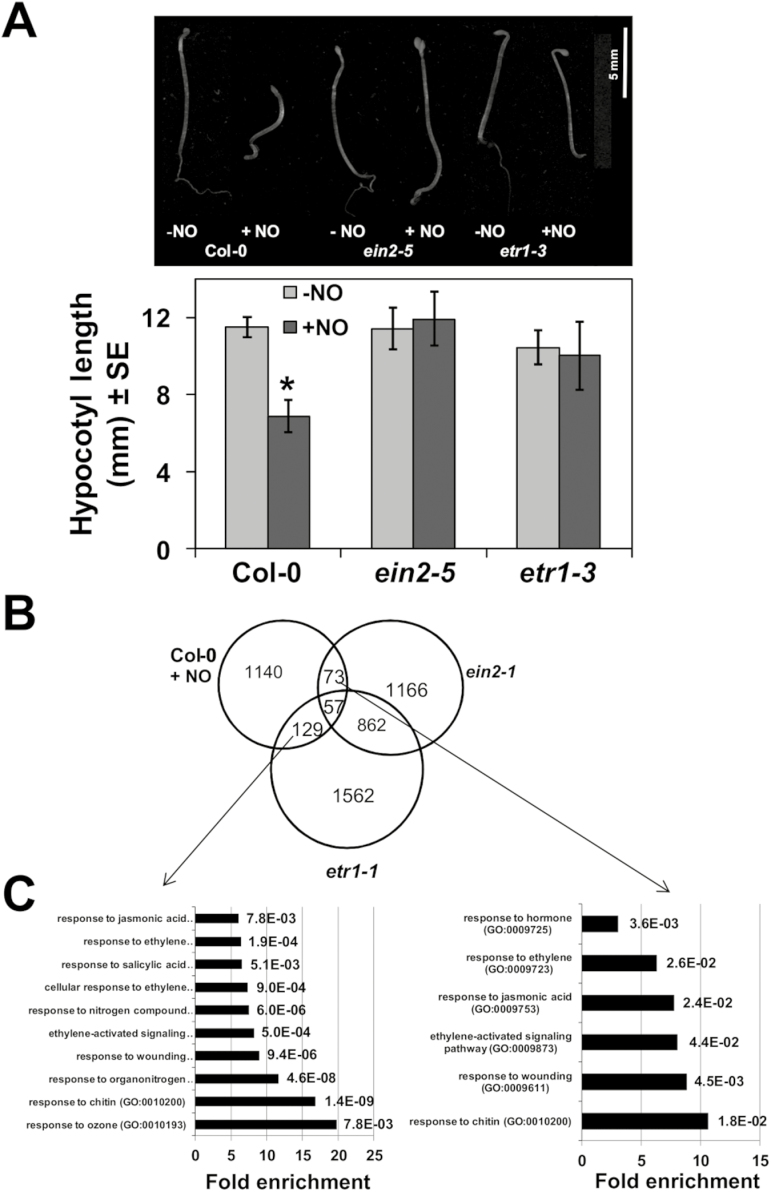
Ethylene perception and signaling is required for NO sensing. (A) Hypocotyl length of untreated (–NO) and NO-treated (+NO) wild-type and ethylene signaling-related mutant seedlings. Values are the mean ±SE (*n*=25) with * representing *P*-values <0.05 in Student’s *t*-test. (B) Venn diagrams representing the intersection between the NO-responsive transcriptome and those differentially regulated in *etr1* and *ein2* mutants. (C) Gene Ontology analysis of the genes found in the intersection between the NO-responsive and *etr1*-regulated (left panel) or *ein2*-regulated (right panel) transcriptomes. Fold enrichment (black bars) and the FDR-corrected *P*-values (at the right side of bars) for the functional categories are shown.

The transcriptome analyses of NO-exposed seedlings and the screening of transgenic plants conditionally expressing TFs suggested the involvement of ethylene signaling in NO-triggered responses. To check whether ethylene perception and signaling are involved in sensing NO, we tested the sensitivity to NO in hypocotyl shortening assays with the ethylene-insensitive *etr1-3* and *ein2-5* mutants (Roman *et al.*, 1995). Figure 3A shows that *etr1-3* and *ein2-5* seedlings were fully insensitive to NO in inhibiting hypocotyl elongation, thus suggesting that ethylene perception and signaling were essential for NO sensing. The comparison of the NO-responsive transcriptome with the differential transcriptomes previously reported for the *ein2-1* and *etr1-1* mutants (Cheng *et al.*, 2009; GEO Accession GSE12715) points to a significant overlap (Fig. 3B). There were 57 genes that were common for the three transcriptomes and an additional 73 and 129 genes in the intersections of the NO-responsive genes and *ein2-1*, and the NO-responsive genes and *etr1-1*, respectively (Fig. 3B), thus supporting a potential relevant involvement of ETR1 and EIN2 in NO sensing. We can rule out that the involvement of ETR1 and EIN2 in NO sensing was due to transcriptional regulation of the corresponding genes by NO, as only slight increases below 1.6-fold in the corresponding transcripts were detected in NO-treated plants (Supplementary Fig. S2A). A Gene Ontology analysis with these groups of commonly regulated genes yielded a significant over-representation of the expected functional categories related to ethylene responses but, of note, also of responses to JA- and salicylic acid (SA)-related functional categories (Fig. 3C).

**Fig. 4.**
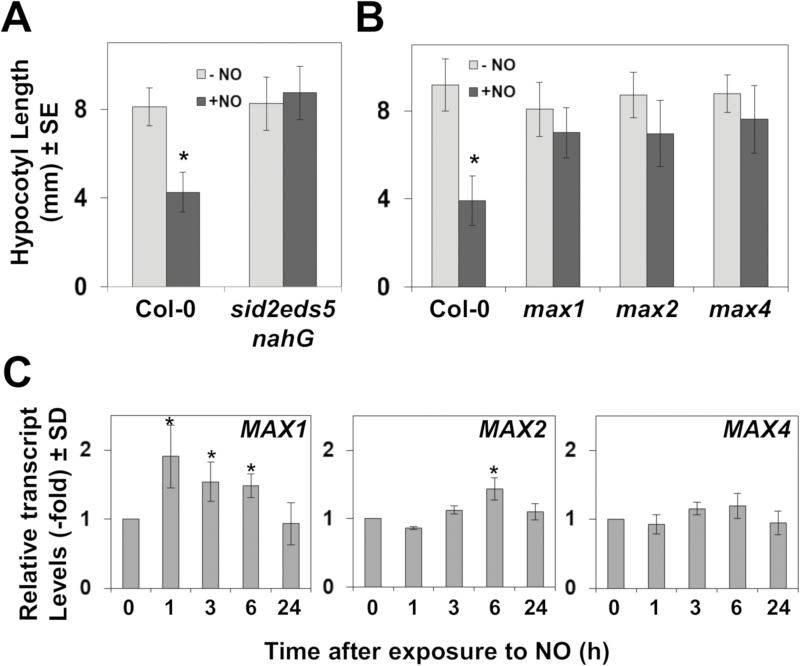
Involvement of salicylates and strigolactones in NO sensing. Hypocotyl length of untreated (–NO) and NO-treated (+NO) (A) wild-type and salicylate-deficient mutant seedlings; and (B) wild-type and strigolactone biosynthesis and signaling mutants. Values are the mean ±SE (*n*=25). (C) Effect of NO treatment on the transcript levels of strigolactone biosynthetic/or signaling-encoding genes. The relative transcript levels were analyzed by RT-qPCR from RNAs obtained at the indicated times after NO exposure of seedlings. Values are the mean ±SD of three independent replicates. *P-values <0.05 in Student’s *t*-test.

Among genes that were similarly regulated by NO treatment in wild-type plants or in untreated ethylene-insensitive mutants (Fig. 3B), we found some that participate in SA-triggered responses. SA is synthesized in Arabidopsis mostly through the isochorismate pathway involving the function of the chloroplast transporter EDS5/SID1 and the isochrorismate synthase 1 (ICS1)/SID2 and ICS2 proteins (Serino *et al.*, 1995; Wildermuth *et al.*, 2001; Garcion *et al.*, 2008). We have tested the sensitivity to NO of SA-deficient *sid2-1eds5-3nahG* plants, which overexpress the *nahG* bacterial gene coding for a SA hydroxylase converting SA to catechol (Gaffney *et al.*, 1993; Delaney *et al.*, 1994) in a genetic background carrying mutations in EDS5/SID1 and ICS1/SID2 genes. Figure 4A shows that, in contrast to wild-type plants, the hypocotyls of etiolated *sid2-1eds5-3nahG* plants were not shortened upon exposure to NO, thus suggesting that the biosynthesis and accumulation of SA is essential for NO sensing in hypocotyls.

On the other hand, we found a significant co-regulation between up-regulated genes in NO-treated wild-type plants and in the SL biosynthesis *max4* mutant (Fig. 1C). SLs are synthesized from the diterpene all-*trans*-β-carotene by the sequential catalysis of D27, MAX3, MAX4, and MAX1, and then SLs are perceived by the D14 receptor, which interacts with the E3 ubiquitin ligase MAX2 that polyubiquitylates negative regulators of the SMXL family and sends them fpr proteasome-mediated degradation (Morffy *et al.*, 2016). We have analyzed whether SLs may be relevant for sensing NO by testing NO-triggered hypocotyl shortening with the biosynthetic and signaling *max1*, *max2*, and *max4* mutants (Sorefan *et al.*, 2003; Booker *et al.*, 2005; Stirnberg *et al.*, 2007). Figure 4B shows that all three SL mutants were insensitive to NO, thus indicating that the NO-triggered inhibition of hypocotyl elongation required the biosynthesis and signaling of SLs, and thus the involvement of these hormones in NO sensing. Only the *MAX1* gene was significantly, although moderately, up-regulated upon exposure to NO (Fig. 4C), thus suggesting that the transcriptional activation of SL biosynthetic and signaling genes was not the limiting step in the SL-mediated NO response mechanism.

### NO sensing and ABA signaling

**Fig. 5.**
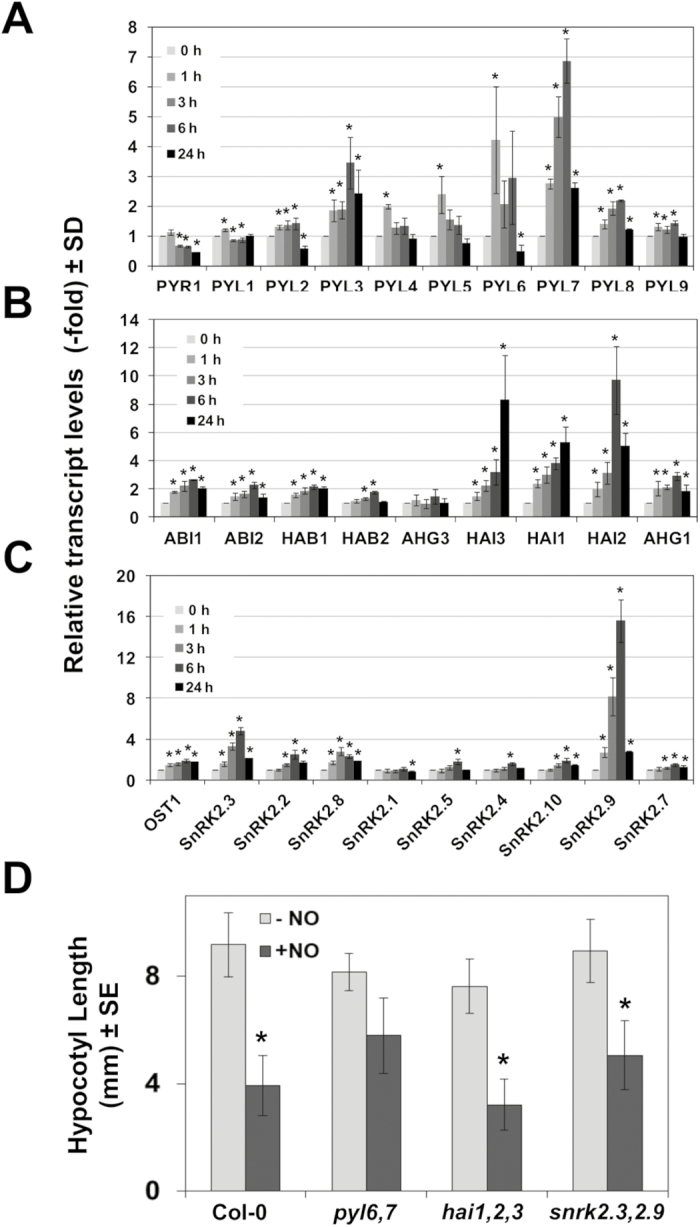
Involvement of ABA signaling in NO sensing. The relative transcript levels of (A) ABA receptor; (B) clade A of protein 2C phosphatase; and (C) SnRK2 family of protein kinase-encoding genes were analyzed by RT-qPCR from RNAs obtained at the indicated times (hours) after NO exposure of seedlings. Values are the mean ±SD of three independent replicates. (D) Hypocotyl length of untreated control (–NO) and NO-treated (+NO) wild-type and ABA signaling mutant seedlings. Values are the mean ±SE (*n* = 25) with * *P*-values <0.05 in Student’s *t*-test.

We have previously reported that NO antagonizes ABA in regulating multiple processes from seed germination and seedling establishment to abiotic stress responses (Lozano-Juste and León, 2010; León *et al.*, 2014). More precisely, the negative effect of NO on ABA perception is mediated, at least in part, by the post-translational Y-nitration and the subsequent inactivation and degradation of PYR/PYL receptors (Castillo *et al.*, 2015). Other positive regulators of the ABA core signaling pathway, such as the kinase OST1/SnRK2.6, were reported to be inactivated through post-translational *S*-nitrosylation of key C residues (Wang *et al.*, 2015a, b). Despite NO exerting regulation on ABA signaling at the post-translational level, our data suggest that NO also regulates ABA signaling at the transcriptional level. The ABA hypersensitivity detected in the NO-deficient *nia1,2noa1-2* mutant plants (Lozano-Juste and León, 2010) correlated well with a significant over-representation of ABA-related genes among up-regulated genes in *nia1,2noa1-2* plants (Gibbs *et al.*, 2014). Finally, further support for the involvement of ABA signaling in NO-triggered responses comes from the identification of several TFs related to ABA signaling in our screening of NO-triggered shortening of hypocotyls in transgenic conditional overexpressing lines (Fig. 2B; Table 1). Those include *BBX31*, which is one of the ABA-specific marker genes, as well as the ABA-up-regulated *STZ/ZAT10*, *PIF3*, *ERF056*, and *RAP2.6L* genes and the down-regulated *MYB30* and *HRS1* genes (Nemhauser *et al.*, 2006). Because ABA perception and signaling involve multicomponent families of receptors and regulators, we decided to check first whether a specific NO-regulated ABA signaling pathway might exist in Arabidopsis. To test this hypothesis, the transcript levels of genes coding for core components of the ABA signaling pathway were analyzed by RT-qPCR at different times after exposure to NO. Figure 5A shows a transient up-regulation of several ABA receptor-encoding genes by 1–6 h after exposure to NO. PYL3, PYL6, and PYL7 were strongly up-regulated, whereas others, such as PYL4, PYL5, and PYL8, were moderately up-regulated (Fig. 5A). Only PYR1 was significantly down-regulated (Fig. 5A). In the next step of the signaling pathway, members of the clade A of type 2C phosphatases (PP2Cs) act as negative regulators of ABA signaling. Although most of the genes coding for ABA-related phosphatases, with the exception of HAB2 and AHG3/PP2CA, were slightly up-regulated in NO-treated plants, only those from the highly ABA-induced subfamily (HAI1, HAI2/AIP1, and HAI3) were strongly up-regulated (>4-fold) by 6–24 h after NO treatment (Fig. 5B). Finally, the next step in ABA signaling is carried out by the positive regulation exerted by protein kinases of the Sucrose non-fermenting 1-Related protein Kinase 2 (SnRK2) family. Among SnRK2-encoding genes, only SnRK2.9 and to a lesser extent SnRK2.3 were up-regulated upon NO treatment (Fig. 5C). From these data, we identified a subset of NO-responsive ABA signaling components including the receptors PYL6, PYL7, and PYL3, the phosphatases HAI1, HAI2/AIP1, and HAI3, and the SnRK2.9 and SnRK2.3 kinases. To check whether these NO-responsive ABA signaling-encoding genes were involved in regulating the plant sensitivity to NO, we tested the effect of exogenously supplied NO on the elongation of hypocotyls from etiolated seedlings of ABA signaling mutant combinations in the NO-responsive genes identified. We generated double *pyl6,7* and *snrk2.3,2.9* mutants, and, together with the available *hai1,2,3* mutant (Bhaskara *et al.*, 2012), these were analyzed in hypocotyl shortening assays. Figure 5D shows that *hai1,2,3* and *snrk2.3,2.9* mutants showed a hypocotyl shortening not significantly different from that detected in wild-type plants, and only the hypocotyls of *pyl6,*7 plants were significantly insensitive to NO.

### NO sensing and jasmonate signaling

**Fig. 6.**
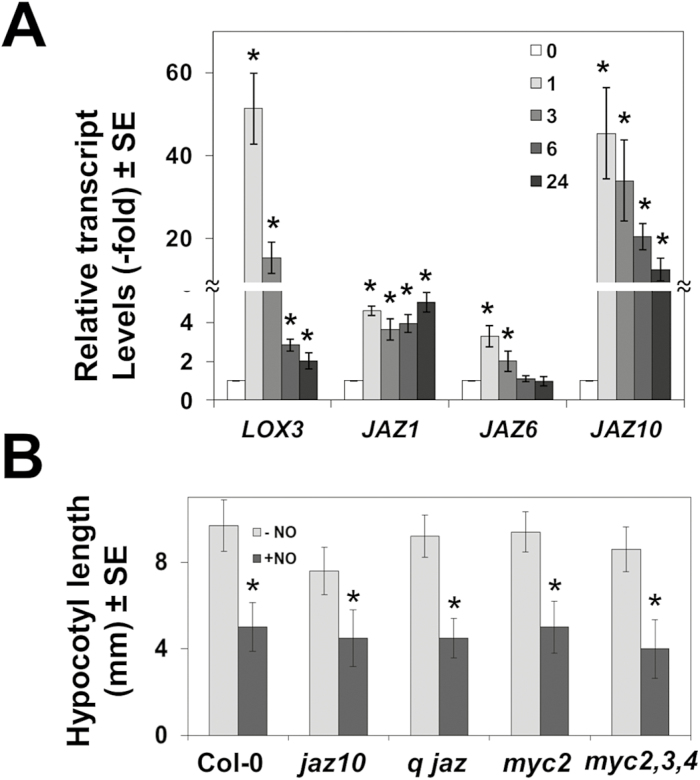
Involvement of jasmonate signaling in NO sensing. (A) Relative transcript levels of the indicated genes were analyzed by RT-qPCR from RNAs obtained at the indicated times (hours) after NO exposure of seedlings. Values are the mean ±SD of three independent replicates. (B) Hypocotyl length of untreated (–) and NO-treated (+NO) wild-type and jasmonate signaling-related mutant seedlings. *q jaz* stands for the quintuple *jaz1,3,5,9,10* mutant. Values are the mean ±SE (*n*=25), with *P-values <0.05 in Student’s *t*-test.

Three independent transcriptome analysis-based lines of evidence suggest that JA may be important for NO sensing mechanisms. We found a significant over-representation of the JA signaling categories among NO-responsive genes (Supplementary Table S3) but also among the particular set of genes that were also differentially expressed in ethylene signaling-deficient mutants (Fig. 3B). Moreover, we found a significant anti-regulation of NO-responsive genes in plants affected in JA perception and signaling (Fig. 1C, D). Among NO-responsive genes listed in Supplementary Table S2, we found that the lipoxygenase-encoding genes *LOX3* and *LOX4*, the 12-oxophytodienoate reductase-encoding gene *OPR1*, and the allene oxide cyclase-encoding gene *AOC3* were all >2-fold up-regulated. Similarly, *JAZ1*, *JAZ5*, *JAZ8*, and *JAZ10* genes coding for different components of the JAZ family of negative regulators of JA signaling were also up-regulated (Supplementary Table S2). Interestingly enough, the gene *JMT* coding for the JA carboxyl methyltransferase, which metabolizes JA to methyl-JA, was strongly down-regulated (Supplementary Table S2). Together, these data suggest the existence of NO-sensitive branches of the JA biosynthetic and signaling pathways. We have confirmed that some JA biosynthetic and signaling genes were up-regulated by NO, thus supporting that the NO-responsive transcriptome identified in the microarray analyses is truly representative. Figure 6A shows that *LOX3* and *JAZ10* were strongly up-regulated (>40-fold), whereas *JAZ1* and *JAZ6* were also up-regulated though more slightly (3- to 4-fold) by 1 h after NO treatment. To explore whether NO-regulated components of the JA signaling pathway modulate NO sensitivity, the response to exogenously supplied NO of JA signaling (*jaz10*, quintuple *jaz1,3,4,9,10*, the single *myc2*, and the triple *myc2,3,4*) mutants was assayed in hypocotyl shortening assays. Figure 6B shows that none of them was significantly different in sensitivity to NO-triggered hypocotyl shortening when compared with wild-type plants. We also checked whether the levels of *MYC* transcripts were regulated in NO-treated plants as shown above for some of the *JAZ* genes (Fig. 6A). *MYC2* was strongly up-regulated upon exposure to NO, whereas *MYC3* was only slightly up-regulated and *MYC4* was not significantly altered (Supplementary Fig. S2B). These findings together suggest that despite the fact that many of the JA signaling component-encoding genes are regulated by NO, this hormone is not involved in NO sensing.

### Involvement of brassinostereoids in NO sensing

**Fig. 7.**
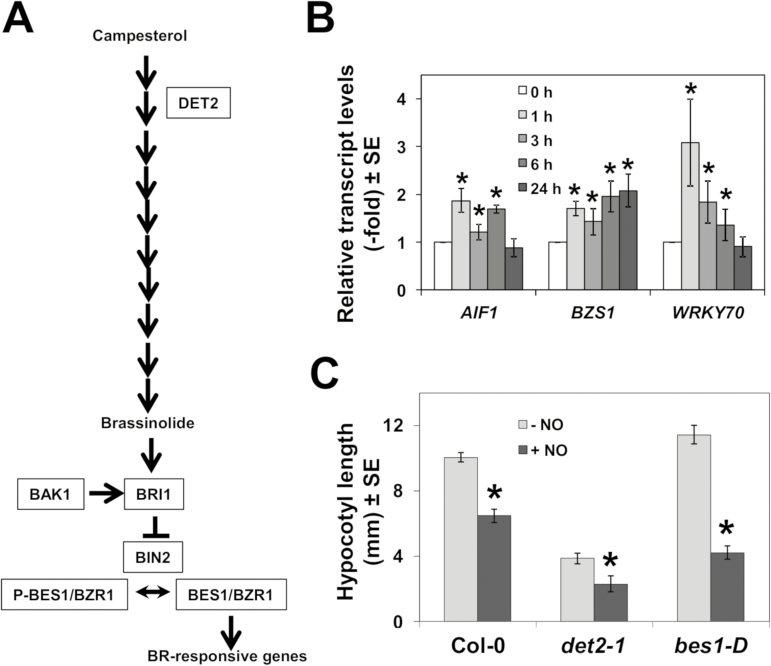
Involvement of brassinosteroid signaling in NO sensing. (A) Scheme of brassinosteroid biosynthesis and signaling pathways. (B) The relative transcript levels of BR-related transcription factor-encoding genes were analyzed by RT-qPCR from RNAs obtained at the indicated times (hours) after NO exposure of seedlings. Values are the mean ±SD of three independent replicates. *P-values <0.05 in Student’s *t*-test. (C) Hypocotyl length of untreated (–NO) and NO-treated (+NO) wild-type and brassinosteroid biosynthesis and signaling mutant seedlings. Values are the mean ±SE (*n*=25). *P-values <0.05 in Student’s *t*-test.

Among genes that were up-regulated by NO at 1 h (Supplementary Table S2), we found a significant overlap (Supplementary Fig. S3) with those that were also up-regulated in responses to BRs (Nemhauser *et al.*, 2006). We also found several TF-encoding genes involved in BR-regulated processes. BRs are synthesized from campesterol through a complex pathway involving 11 steps (Fig. 7A). The second step catalyzed by DET2 seems to be a determinant for BR biosynthesis, in such a way that *det2* mutant plants are severely impaired in BR production (Fujioka *et al.*, 1997; Noguchi *et al.*, 1999). Once the active BR brassinolide is synthesized, it is further perceived by the BRI1 receptor (Jiang *et al.*, 2013), which is controlled by reversible phosphorylation by BAK1 (He *et al.*, 2013). Then, BRI1 negatively regulates BIN2, which in turn modulates the phosphorylation state of the transcriptional activators BES1/BZR1 that accumulate in the nucleus (Yin *et al.*, 2002), triggering BR-responsive gene expression (Fig. 7A). We have confirmed that *AIF1*, *BZS1* , and *WRKY70*, which are involved in BR-regulated control of plant growth and responses to stress (Wang *et al.*, 2009; He *et al.*, 2005; Chen *et al.*, 2017), were up-regulated by NO to different extents (Fig. 7B), thus pointing to BR signaling as a target of NO action. To check whether BR biosynthesis was necessary for NO sensing in hypocotyls, we analyzed the loss-of-function *det2-1* mutant in DE-ETIOLATED2 that is severely BR deficient and dwarf (Chory *et al.*, 1991). The hypocotyls of etiolated *det2-1* seedlings, despite being short, were still fully sensitive to NO in inhibiting hypocotyl elongation, thus indicating that biosynthesis of BRs is not required for NO sensing. Moreover, the dominant gain-of-function *bes1-D* mutant in BRI1-EMS-SUPPRESSOR 1 (BES1)/BRASSINAZOLE-RESISTANT 2 (BZR2) (Shin *et al.*, 2016), despite displaying longer hypocotyls in non-treated seedlings, was hypersensitive to NO in shortening their hypocotyls (Fig. 7C), thus suggesting that BES1-mediated BR signaling potentiates NO sensing. It is worth mentioning that in accordance with BES1 being an NO target, the *BES1* gene was strongly down-regulated by NO (Supplementary Fig. S2C), thus representing a potential node for self-controlled sensing.

## Discussion

Despite the increasing characterization of NO-triggered responses, the mechanisms underlying NO perception/sensing in plants remain largely unknown. This study is focused on understanding the early processes after plants are exposed to a pulse of exogenous NO. We previously reported that endogenous NO positively regulates photomorphogenesis through the control of processes such as hypocotyl elongation (Lozano-Juste and León, 2011) or apical hook opening (Abbas *et al.*, 2015) in etiolated seedlings. Here, in this work, we have combined the information obtained from a transcriptomic approach with plants exposed to an NO pulse, with an NO sensitivity test based on the inhibition of hypocotyl elongation after exposure of etiolated seedlings to NO. Transcriptome data reveal that the first change in gene expression occurred <1 h after exposure to NO (Fig. 1). The mode of NO action is mainly based on the rapid reaction with other molecules, which occurs on the time scale of seconds to a few minutes in water-based environments (Olasehinde *et al.*, 2010). The lag in the transcriptome response is likely to be due to the time required for the activation of the transcriptional machinery. We previously found that the first significant altered patterns of NO-related post-translational protein modifications, such as nitration and *S*-nitrosylation, are observed by 1 h after exposure to NO pulse and preceded transient metabolic changes occurring by 6 h after exposure (León *et al.*, 2016). Therefore, the extensive changes in the transcriptome and in the nitrated or *S*-nitrosylated proteome are coincident in time. These data suggest that, in the absence of a specific NO receptor such as the GC in mammals (Gross and Durner, 2016), the NO-triggered post-translational modifications of proteins with signaling potential primarily sense NO, and then transmit the corresponding signal to other amplifying signaling proteins. The large numbers of genes coding for proteins involved in the biosynthesis and signaling of multiple hormones that we found in the transcriptomic analyses (Supplementary Tables S2, S3) suggest that the hormone biosynthesis and signaling pathways are direct targets of NO action. It has been proposed that NO acts as a key component in different hormone-regulated processes (Simontacchi *et al.*, 2013). Alternatively, NO–hormone interactions could be considered as an indication of NO using some components of the hormone signaling pathways to achieve sensing and further signal transduction. Whether NO-triggered alterations in hormone signaling are connected or not with NO sensing has been addressed in this study by a genetic approach. NO-triggered hypocotyl shortening was tested in hormone biosynthesis and signaling mutant seedlings. Mutants in either the perception (*etr1-3*) or signaling (*ein2-5*) of ethylene did not show any significant hypocotyl shortening and are thus insensitive to NO (Fig. 3). Ethylene signaling requirement connects our findings here with our previous report identifying the role of group VII of ethylene response factors (ERFVII) as NO sensors (Gibbs *et al.*, 2014). The ERFVII-based mechanism of NO sensing relies on the specific O_2_- and NO-dependent oxidation of the C2 N-terminal residue of these transcription factors allowing further modifications and the subsequent ubiquitylation and proteasomal degradation. Regarding this, the *prt6* mutant, null for the function of the E3 ubiquitin ligase PRT6 and thus unable to ubiquitylate ERFVIIs, is also insensitive in NO-triggered hypocotyl shortening assays (Gibbs *et al.*, 2014). It is noteworthy that the genes coding for the Plant Cysteine Oxidase 1 and 2, which catalyze the O_2_- and NO-dependent oxidation of the C2 N-terminal residue of ERFVIIs (Weits *et al.*, 2014; White *et al.*, 2017), were strongly up-regulated by NO (Supplementary Table S2), thus suggesting that the N-end rule-based NO-sensing mechanism is also relevant under the conditions tested in this work. Furthermore, it has been reported that the *ein2* mutant has shorter hypocotyls than wild type plants in the light, suggesting that the ethylene signaling participates in promoting hypocotyl elongation in the light (Smalle *et al.*, 1997). However, *ein2-5* mutant hypocotyls are indistinguishable from those of the wild type in length under darkness (Fig. 3A), thus suggesting that ethylene signaling is not required to elongate hypocotyls under skotomorphogenic conditions. The ethylene receptor *etr1-3* mutant behaves similarly to *ein2-5* (Fig. 3A), thus suggesting that ethylene perception and signaling are, in turn, necessary to inhibit hypocotyl elongation by NO under darkness, and thus point to a key role for ethylene signaling in NO sensing. The functional interactions between NO and ethylene in plants are complex and sometimes controversial. Reports describing NO as a potent inducer of ethylene biosynthesis in apple embryos (Gniazdowska *et al.*, 2007) co-exist with others that report on inhibition of ethylene biosynthesis by NO through the inactivation of ACC (1-aminocyclopropane-1-carboxylic acid) synthase (Montilla-Bascón *et al.*, 2017) and methionine adenosyltransferase (Lindermayr *et al.*, 2006). Despite the complex interaction in terms of hormone biosynthesis, it has recently been reported that the NO control of cell cycle progression requires the function of EIN2 in Arabidopsis cell cultures (Novikova *et al.*, 2017), thus suggesting that this mechanism could be the basis of the hypocotyl growth inhibition by NO we describe in this work.

Hypocotyl NO sensing not only requires ethylene perception and signaling, but also seems to be dependent on SL biosynthesis and signaling (Fig. 4B). SLs are required for the NO-mediated root apex growth in maize (Manoli *et al.*, 2016), and also for the NO-induced root elongation in rice under phosphate and nitrogen deficiency (Sun *et al.*, 2016). In contrast, SLs inhibited hypocotyl elongation (Jia *et al.*, 2014). Remarkably, SL mutants were all fully insensitive to NO-triggered inhibition of hypocotyl elongation (Fig. 4B), thus suggesting that NO requires SL biosynthesis and signaling to inhibit hypocotyl elongation. These data point to NO as a potential key factor in SL-exerted opposite regulatory effects in root and shoot growth. As recently reviewed (Kolbert 2018), the functional SL–NO interaction seems to be quite complex. It seems that NO positively regulates SL signaling but it does not influence either SL production or the expression of SL biosynthetic genes in rice roots (Sun *et al.*, 2016). In contrast, the exogenous application of SLs induced the production of NO (Kolbert, 2018; Lv *et al.*, 2018), but it remains unclear whether NO actually induce the production of SLs. We found that the exogenous application of NO mildly activated the expression of *MAX1* and even to a lesser extent *MAX2* genes (Fig. 4C), thus suggesting in this experimental system that NO might trigger SL production, although this has to be confirmed by measuring SL levels in NO-treated seedlings. Moreover, the rather low induction of *MAX* genes suggested that SL biosynthesis and signaling should not be rate-limiting steps in NO-triggered responses, although MAX protein levels and activity should be analyzed to support this hypothesis. On the other hand, salicylate biosynthesis and accumulation seem also to be essential for hypocotyl NO sensing, as *sid2eds5nahG* plants were fully insensitive in NO-induced inhibition of hypocotyl elongation (Fig. 4A). Remarkably, SL application triggers the biosynthesis of SA (Rozpądek *et al.*, 2018). Thus our findings point to a potential signaling cascade involving the NO-triggered production of SLs, which in turn would activate the biosynthesis of salicylates. However, this scenario might be more complicated considering that the metabolic flux described above could also function in the opposite direction, as it has been reported that salicylates can induce the production of NO (Zottini *et al.*, 2007; Hao *et al.*, 2010; Tari *et al.*, 2011), and also that SLs may either enhance or decrease the endogenous NO content in sunflower roots (Bharti and Bhatla, 2015). This sort of reciprocal regulatory effects could be the basis of a self-regulatory hormonal loop involved in ensuring the correct NO sensing under different conditions and/or organ/cellular locations.

We also identified some ABA signaling genes that were up-regulated in NO-exposed plants (Fig. 5; Supplementary Table S2). Because mutants in some of these NO-inducible ABA signaling genes were less sensitive to NO than wild-type plants in hypocotyl shortening assays, these components might also be potential targets of NO sensing/action. This seems to be the case for the NO-inducible PYL6 and PYL7 receptors of ABA, as the NO-exposed hypocotyls of the double *pyl6,7* mutant seedlings were not significantly different in length from those which were untreated (Fig. 5B). Although PYL7 remains as one of the less studied ABA receptors of the PYR/PYL/RCAR family, it is noteworthy that PYL7 interacts preferentially with the type 2C protein phosphatases of the HAI family (Bhaskara *et al.*, 2012) and AHG1 (Tischer *et al.*, 2017), which we have found to be the PP2C-encoding genes more strongly up-regulated by NO (Fig. 5A). The existence of a subset of ABA signaling genes, which might be regulated both at the transcriptional and, potentially, at the post-translational level by NO might be suggested. We previously reported a mechanism of inactivation of PYR/PYL/RCAR receptors, which is based on the NO/peroxynitrite-triggered nitration of tyrosine residues and the concomitant polyubiquitylation and proteasome-mediated degradation of the receptors (Castillo *et al.*, 2015). The central ABA regulator ABI5 has been reported to be *S*-nitrosylated and further degraded, thus promoting seed germination (Albertos *et al.*, 2015). NO–ABA functional interactions have been reported to be relevant in a wide array of developmental and stress-related responses (León *et al.*, 2014; Sanz *et al.*, 2015).

We found that the gain of function in the BR signaling *bes1-D* mutant was more sensitive to NO in hypocotyl shortening assays than wild-type seedlings (Fig. 7C). The interaction between the BR signaling BES1 TF and TOPLESS controls shoot and root development (Espinosa-Ruiz *et al.*, 2017). Moreover, the NF-YC4 TF-encoding gene, which was up-regulated by both NO and BR (Supplementary Fig. S3), has been reported to control photomorphogenesis (Myers *et al.*, 2016). NF-YC4 acts as a repressor of hypocotyl elongation (Tang *et al.*, 2017), thus supporting the functional connection between NO and BRs in controlling the length of hypocotyls in etiolated seedlings.

It seems that NO control of hypocotyl growth may involve multiple hormone-related targets including SL biosynthetic MAX1 and MAX4 enzymes as well as MAX2 signaling protein, ethylene signaling ETR1 and EIN2 proteins, ABA perception PYL6 and PYL7 proteins, and finally the BR BES1/BZR2 TF. NO could alter the function of those proteins by modifying them either through nitrosothiol-mediated *S*-nitrosylation of cysteine residues or through peroxynitrite-mediated nitration of tyrosine residues. We performed an *in silico* prediction for these types of post-translational modifications for those potential targets. Supplementary Table S5 shows that all analyzed proteins may be potentially *S*-nitrosylated or nitrated. Among them, some residues were predicted to be more likely to be modified as prediction coincided in two different platforms. This is the case for the *S*-nitrosylation of C1063 and C1218 of EIN2, and C63 of MAX2, as well as for the nitration of Y783 of EIN2 and Y176 of PYL6 (Supplementary Table S5). However, these are just predictions and none of these proteins has been identified yet as post-translationally modified. Further proteomic work will clarify whether the NO-related post-translational modifications of these signaling proteins could be important for the NO-sensing mechanisms operating in etiolated hypocotyls.

All these data together suggest that NO sensing in Arabidopsis hypocotyls essentially requires the biosynthesis and/or signaling of ethylene, SLs, salicylates, and ABA, and that the negative regulation of BR signaling genes such as *BES1* by NO also contribute to the NO-triggered inhibition of hypocotyl elongation. The fact that mutations in genes involved in only one of the hormone signaling pathways, such as ethylene, SLs, or salicylates, were enough to make plants fully insensitive to NO seems to rule out the possibility that the different pathways operate in parallel in integrating NO sensing. It seems more likely that different hormones integrate NO responses by acting in a cascade-like or highly interconnected network, with some nodes being more quantitatively relevant than others. Whether this model for NO sensing in hypocotyls can be extrapolated or not to other organs, such as roots, or stomata in leaves, will require further work as the endogenous levels of NO and hormones change dramatically from organ to organ, or even in different tissues in the same organ.

## Supplementary data

Supplementary data are available at *JXB* online.

Fig. S1. β-Estradiol-induced transcript accumulation in randomly selected TRANSPLANTA transgenic lines.

Fig. S2. Effect of NO treatment on the transcript levels of ethylene, strigolactone, jasmonate, and brassinosteroid biosynthetic/or signaling-encoding genes.

Fig. S3. Genes up-regulated by brassinolide (BL) or NO treatments.

Table S1. Oligonucleotides used in this work.

Table S2. Microarray analyses for identifying the early NO-responsive transcriptome.

Table S3. Gene Ontology analyses of NO-responsive genes.

Table S4. TPT lines that were screened for a differential effect of NO on hypocotyl elongation under darkness.

Table S5. Prediction of *S*-nitrosylation and nitration sites of potential NO targets.

Supplementary Figures and Tables
