# Supplementary material for: Nitric oxide responses in Arabidopsis hypocotyls are mediated by diverse phytohormone pathways
Source: J Exp Bot. 2018 Aug 2;69(21):5265–78. doi: 10.1093/jxb/ery286 (PMC6184486; doi:10.1093/jxb/ery286)
Supplement: Supplementary Figures and Tables [file ery286_suppl_supplementary_figures-s1-s3_tables-s1-s5.pdf]

Supplementary data

Title: Nitric oxide responses in Arabidopsis hypocotyls are mediated by diverse phytohormone pathways

Authors: Mari-Cruz Castillo[§], Alberto Coego[§], Álvaro Costa-Broseta[§], José León

Affiliation: Instituto de Biología Molecular y Celular de Plantas (Consejo Superior de Investigaciones Científicas–Universidad Politécnica de Valencia), 46022 Valencia, Spain

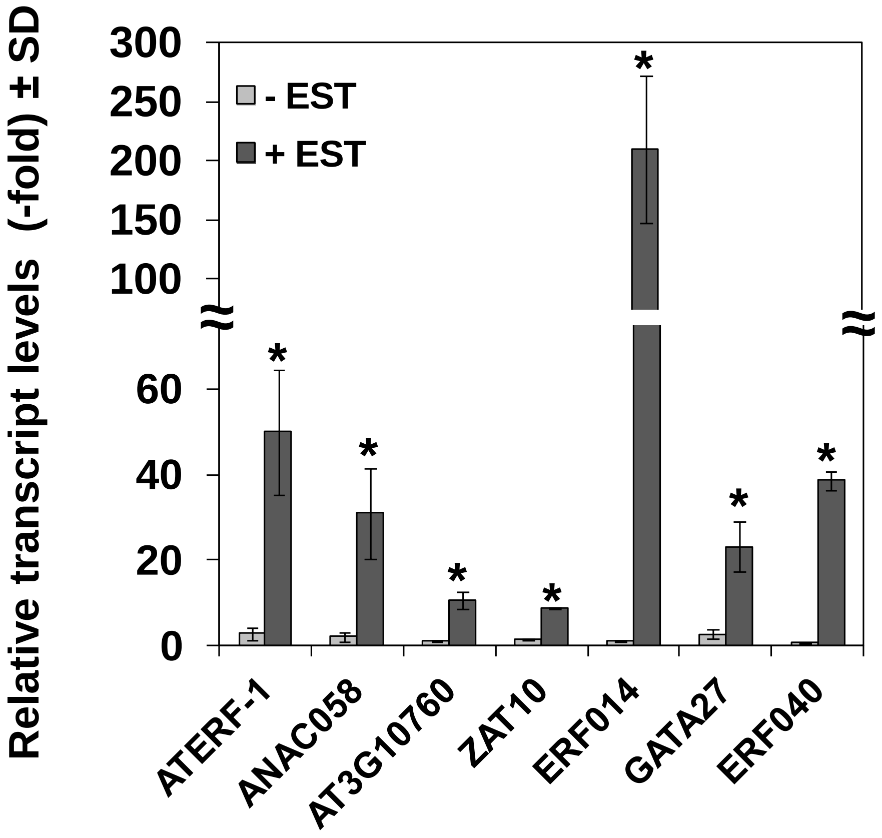

Supplementary Fig. S1. β-estradiol induced transcript accumulation in randomly selected TRANSPLANTA transgenic lines. The relative transcript levels were analyzed by RT-qPCR from RNAs obtained at the indicated times after NO exposure of seedlings. Values are normalized to the levels in wild type plant and represent the mean ± SD of three independent replicates. * representing p-values <0.005 in Student´s t-test.

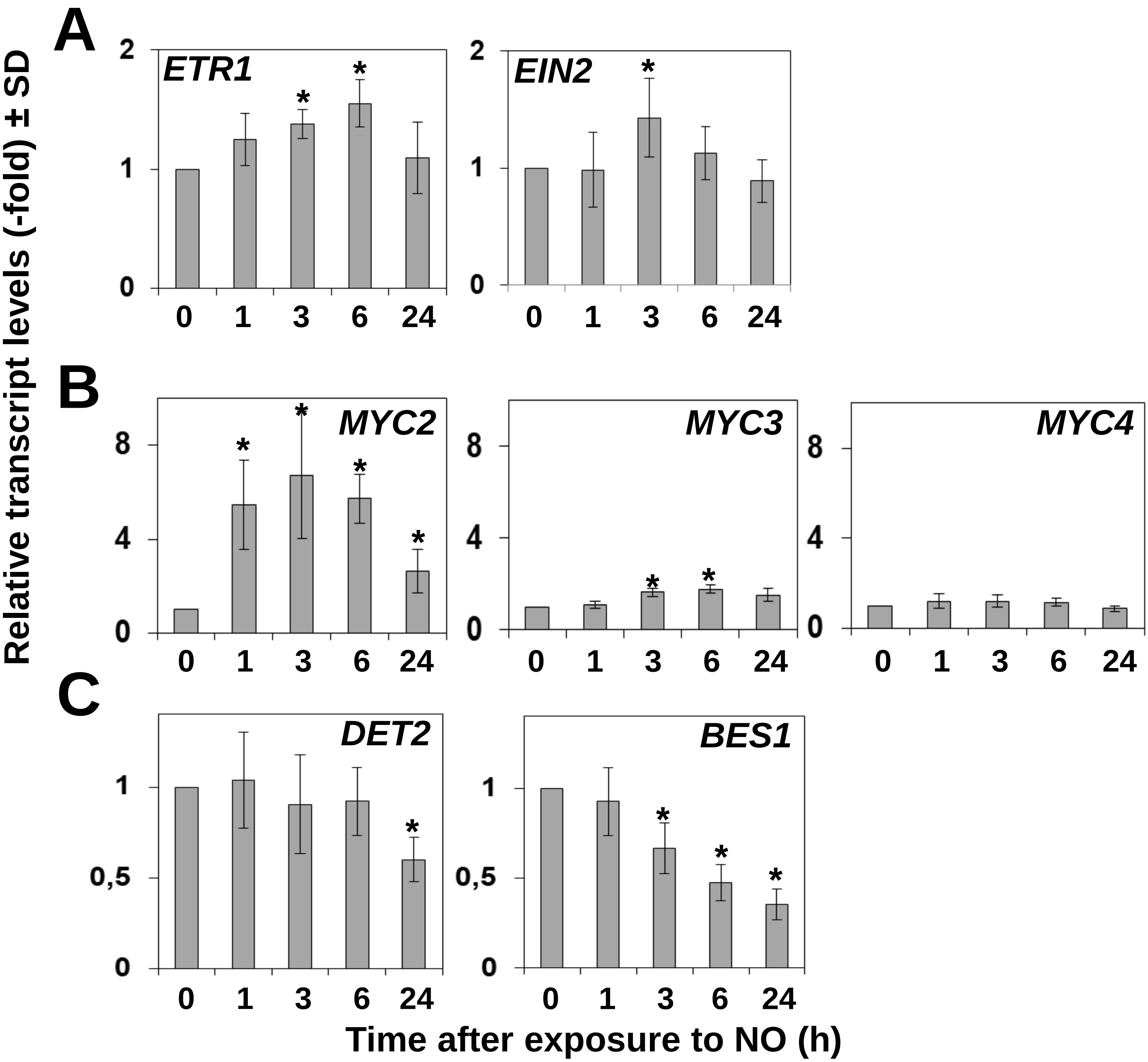

Supplementary Fig. S2. Effect of NO treatment on the transcript levels of A, ethylene, B, jasmonate and C, brassinosteroid biosynthetic/or signaling encoding genes. The relative transcript levels were analyzed by RT-qPCR from RNAs obtained at the indicated times after NO exposure of seedlings. Values are the mean ± SD of three independent replicates. * representing p-values <0.05 in Student´s t-test.

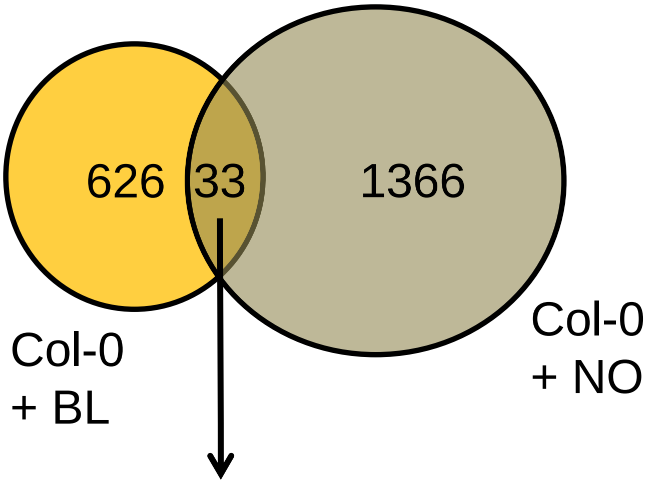

At4g31800 ATWRKY18_WRKY18__WRKY DNA-binding protein 18
At4g25810 XTH23_XTR6__xyloglucan endotransglycosylase 6
At3g26085 CAAX amino terminal protease family protein
At5g15850 ATCOL1_BBX2_COL1__CONSTANS-like 1
At3g28340 GATL10_GolS8__galacturonosyltransferase-like 10
At5g13220 JAS1_JAZ10_TIFY9__jasmonate-zim-domain protein 10
At3g60160 ABCC9_ATMRP9_MRP9__multidrug resistance-associated protein 9
At4g11280 ACS6_ATACS6__1-aminocyclopropane-1-carboxylic acid (acc) synthase 6
At1g75460 ATP-dependent protease La (LON) domain protein
At1g24280 G6PD3__glucose-6-phosphate dehydrogenase 3
At4g37980 ATCAD7_CAD7_ELI3_ELI3-1__elicitor-activated gene 3-1
At4g27970 SLAH2__SLAC1 homologue 2
At1g32920 Unknown
At5g45340 CYP707A3__cytochrome P450, family 707, subfamily A, polypeptide 3
At1g01420 UGT72B3__UDP-glucosyl transferase 72B3
At1g55545 Unknown
At2g26530 AR781__Protein of unknown function (DUF1645)
At4g30280 ATXTH18_XTH18__xyloglucan endotransglucosylase/hydrolase 18
At1g23080 ATPIN7_PIN7__Auxin efflux carrier family protein
At5g15950 Adenosylmethionine decarboxylase family protein
At5g63470 NF-YC4__nuclear factor Y, subunit C4
At1g17990 Putative 12-oxophytodienoate reductase-like protein 2A
At5g64780 Uncharacterised conserved protein UCP009193
At4g25780 CAP (C-rich secretory, Antigen 5, and Pathogenesis-related 1) superfamily protein
At3g57450 Unknown
At5g65300 Unknown
At1g29460 SAUR65__SAUR-like auxin-responsive protein family
At3g50560 NAD(P)-binding Rossmann-fold superfamily protein
At2g04240 XERICO__RING/U-box superfamily protein
At3g23550 MATE efflux family protein
At2g04230 FBD, F-box and Leucine Rich Repeat domains containing protein
At3g49620 DIN11__2-oxoglutarate (2OG) and Fe(II)-dependent oxygenase superfamily protein
At1g10550 XET_XTH33__xyloglucan:xyloglucosyl transferase 33

Involved in ABA signaling, ethylene biosynthesis, auxin signaling, jasmonate signaling, cell wall loosening and cell elongation besides NO- and BR-triggered responses

Supplementary Fig. S3. Genes up-regulated by either brassinolide (BL) or NO treatments. The color codes corresponded to the functional categories outlined at the end of the table.

Supplementary Table S1. Oligonucleotides used in this work.

**Name**	**Sequence (5´to 3´)**	**AGI**	**Application**
qP-ACT2-R	TGTCTCGTGGATTCCAGCAG	AT3G18780	qRT-PCR
qP-ACT2-F	TTGTTCCAGCCCTCGTTTGT	AT3G18780	qRT-PCR
qPYR1-R	CGTCGTACGATTGACCAGACGAG	AT4G17870	qRT-PCR
qPYR1-F	GCCTTCGGAGTTAACACCAGAAG	AT4G17870	qRT-PCR
qPYL1-R	GGGAGAGTTGGGTGAATTCG	AT5G46790	qRT-PCR
qPYL1-F	GGCGAATTCAGAGTCCTCCTCC	AT5G46790	qRT-PCR
qPYL2-R	GCCGACGACCCTGAAGCT	AT2G26040	qRT-PCR
qPYL2-F	CAGTACCGAGCGGCTTGAG	AT2G26040	qRT-PCR
qPYL3-R	CGAAGTCGCGGACGAATCT	AT1G73000	qRT-PCR
qPYL3-F	CATCACTCATAGCACACCGTGTAG	AT1G73000	qRT-PCR
qPYL4-R	CGCGATCATCATCGGAATC	AT2G38310	qRT-PCR
qPYL4-F	GCTTGCCGTTCACCGTCCTTC	AT2G38310	qRT-PCR
qPYL5-F	GGTCACCGGTGCAACTCC	AT5G05440	qRT-PCR
qPYL5-R	CGCGTGGATCATCTGCACC	AT5G05440	qRT-PCR
qPYL6-R	CCTCCACGTCTTGTACCACG	AT2G40330	qRT-PCR
qPYL6-F	CCAACGTCGATACAGTTTCAG	AT2G40330	qRT-PCR
qPYL7-R	AGTGGTGAAGATGACGCAACCT	AT4G01026	qRT-PCR
qPYL7-F	GATCGGAGGAGACGATACAGATACA	AT4G01026	qRT-PCR
qPYL8-R	CGGCTTATACTTCTGTGGC	AT5G53160	qRT-PCR
qPYL8-F	GGAAGCTAACGGGATTGAG	AT5G53160	qRT-PCR
qPYL9-R	ATGCGTCCGTACGTATTGCA	AT1G01360	qRT-PCR
qPYL9-F	GATATGATGGACGGCGTTGAA	AT1G01360	qRT-PCR
qHAI1-R	CTCGCACCGGCATTTTG	AT5G59220	qRT-PCR
qHAI1-F	ACGCGCATGGACATGGA	AT5G59220	qRT-PCR
qHAI2-R	ACACATGCGCACCATCGTA	AT1G07430	qRT-PCR
qHAI2-F	GCGACGGACGGGCTATG	AT1G07430	qRT-PCR
qHAI3-F	CCGTCGTCGGTTTATGAATCA	AT2G29380	qRT-PCR
qHAI3-R	TGCATCACCGTTTGGAATCTC	AT2G29380	qRT-PCR
qHAB2-R	CCCACCAGCCGTTTATTCTC	AT1G17550	qRT-PCR
qHAB2-F	CTGTTCAGCGGAGGTACATACTTC	AT1G17550	qRT-PCR
qHAB1-R	CTGAGTGATCTCGACAGGTGATG	AT1G72770	qRT-PCR
qHAB1-F	TCCCGCAGTTGCAATGACT	AT1G72770	qRT-PCR
qABI2-R	CACCGTTGCAATAGCCTCTAAGT	AT5G57050	qRT-PCR
qABI2-F	CTGCAGTCGCTGTTCCATTC	AT5G57050	qRT-PCR
qAB1-F	TTCTCCGAAACCCAGATGGA	AT4G26080	qRT-PCR
qABI1-R	CGTTCTCGGAATCTTGATTTGAG	AT4G26080	qRT-PCR
qAHG1-F	CGAGGGAGGCTGCGATTT	AT5G51760	qRT-PCR
qAHG1-R	ACAATAATATGATCGTGCGTCAAAA	AT5G51760	qRT-PCR
qAHG3-F	TTGTTGCGGTGTTGTTGGA	AT3G11410	qRT-PCR
qAHG3-R	AGAGAAGCTCGAGAAGTTGAATCA A	AT3G11410	qRT-PCR
qSnRK2.10-R	ACTACACAAAGTCACAAACCCAGAAA	AT1G60940	qRT-PCR
qSnRK2.10-F	TTCTATCACTTTGTGCCTTTTTTAGC	AT1G60940	qRT-PCR
qSnRK2.4-R	CAAAATCAAGGATGCGATTCC	AT1G10940	qRT-PCR
qSnRK2.4-F	CATCGTCATCTCTTTCCCTTT	AT1G10940	qRT-PCR
qSnRK2.5-R	CAATACCTTCACAACCTCATACTTGT	AT5G63650	qRT-PCR
qSnRK2.5-F	CAACGAGAGATCGTGATCGTACTT	AT5G63650	qRT-PCR
qSnRK2.1-R	TGGCTTTGACAAATGTGTTTTTCT	AT5G08590	qRT-PCR
qSnRK2.1-F	TGCTCT CTGTTTCTCTTACTTTATTTCTTC	AT5G08590	qRT-PCR
qSnRK2.7-F	TTTCCTCCGAGTGCAAGCAT	AT4G40010	qRT-PCR
qSnRK2.7-R	GTGCTTTTCGATTTCCGGTACA	AT4G40010	qRT-PCR
qSnRK2.8-R	CGGCGCTGCAGATTCTTC	AT1G78290	qRT-PCR
qSnRK2.8-F	GGCAACACATTTGGCGTTAGT	AT1G78290	qRT-PCR
qSnRK2.2-R	TCAAGATTATCCGCCATGAAATC	AT3G50500	qRT-PCR
qSnRK2.2-F	GCAGATAATATCGGAGGCTACGA	AT3G50500	qRT-PCR
qSnRK2.3-R	ACTGTCGTGCATAATCGGCATA	AT5G66880	qRT-PCR

qSnRK2.3-F	TCGAATTTCTCTTTTTGTGATCAGA	AT5G66880	qRT-PCR
qOST1-R	TGCTTCTGCAATGATCTGCAT	AT4G33950	qRT-PCR
qOST1-F	CACGATGACCACTCAGTTTGATG	AT4G33950	qRT-PCR
qSnRK2.9-R	CACAAGCTCGTTTGTTTGCTTATT	AT2G23030	qRT-PCR
qSnRK2.9-F	GGAGAAGTATGAGATGGTGAAGGATT	AT2G23030	qRT-PCR
qAIF1-R	GACGTAGAGCCTCCATCAGC	AT3G05800	qRT-PCR
qAIF1-F	ATCGAATCCTTCCACGTCAC	AT3G05800	qRT-PCR
qBZS1-R	TCCGGTTCTCTCTCACTTCTC	AT4G39070	qRT-PCR
qBZS1-F	CCGGTAAGGAGGAATCTATTGTG	AT4G39070	qRT-PCR
qDET2-F	TCCTCAATGGTTATATCCAGGCGA	AT2G38050	qRT-PCR
qDET2-R	TCTTCAATCGTACCAAAGTCCGGT	AT2G38050	qRT-PCR
qBES1-F	AACTACCCGTTTTATGCGGTGTCTG	AT1G19350	qRT-PCR
qBES1-R	GTTGGCACCATAGAGGCAGAGAAT	AT1G19350	qRT-PCR
qWRKY70-R	TGGGAGTTTCTGCGTTGG	AT3G56400	qRT-PCR
qWRKY70-F	AAAAGATTGGGACCCGTTAA	AT3G56400	qRT-PCR
qMYC2-F	GTGCGGGATTAGCTGGTAAA	AT1G32640	qRT-PCR
qMYC2-R	ATGCATCCCAAACACTCCTC	AT1G32640	qRT-PCR
qMYC3-F	TGTTGAAGCAGAGAGGCAGA	AT5G46760	qRT-PCR
qMYC3-R	CTCCGAGAAGCGAAGCTTTA	AT5G46760	qRT-PCR
qMYC4-F	AGGAGCAAACGAGAACTGGA	AT4G17880	qRT-PCR
qMYC4-R	CCATCTCCCCAACCTAACAA	AT4G17880	qRT-PCR
qJAZ1-F	AGCTTCACTTCACCGGTTCTTGGA	AT1G19180	qRT-PCR
qJAZ1-R	TCTTGTCTTGAAGCAACGTCGTCA	AT1G19180	qRT-PCR
qJAZ3-F	TGTAATGGCTCCAACAGTGGCATTAC	AT3G17860	qRT-PCR
qJAZ3-R	ATTCAGACATTGATCTGCGACAATCTGT	AT3G17860	qRT-PCR
qJAZ6-F	TCATCTTCCTCCCAAGCCAGAGAT	AT1G72450	qRT-PCR
qJAZ6-R	ACTAGAAACGTGAACTCGATCGTGCAT	AT1G72450	qRT-PCR
qJAZ10-F	TCGCAAGGAGAAAGTCACTGCAAC	AT5G13220	qRT-PCR
qjAZ10-R	CGATTTAGCAACGACGAAGAAGGC	AT5G13220	qRT-PCR
qLOX3-F	CGGATAGAGAAAGAGATTGAGAAAAGGAAC	AT1G17420	qRT-PCR
qLOX3-R	AGGTACACCTCTACACGTAACACCAGGC	AT1G17420	qRT-PCR
qMAX1-F	GGCCCTATTTTCAGATTTCAG	AT2G26170	qRT-PCR
qMAX1-R	TGGTGAAGAAGAGGCCTTTC	AT2G26170	qRT-PCR
qMAX2-F	CCGGAGAACGATATGAGCAC	AT2G42620	qRT-PCR
qMAX2-R	CAAGTTTCAGTCAATGATGTTGC	AT2G42620	qRT-PCR
qMAX4-F	GTTTTACCCGATGCTAGGATC	AT4G32810	qRT-PCR
qMAX4-R	TGATGCTGCACATATCCATCG	AT4G32810	qRT-PCR
qETR1-F	TTCCCCGACATTCAAATTTCAC	AT1G66340	qRT-PCR
qETR1-R	GTGGATTTGTCAGTGTTACCAC	AT1G66340	qRT-PCR
qEIN2-F	TGAGACCTCAGCTAGGGTTTATC	AT5G03280	qRT-PCR
qEIN2-R	TAAGTGCATGCGCAACTCCCAC	AT5G03280	qRT-PCR

Supplemental Table S2. Microarray analyses for identifying the early NO-responsive transcriptome.

Description of microarray experiments according to MIAME

**Investigation Design Format (IDF)**	
Investigation title	IBMCP JLeon lab NO-exposed (15, 30 and 60 min after pulse of 300 ppm NO during 5 min) wt Col-0 versus untreated wt Col -0
Experimental designs	NO-treated vs untreated wild type Col-0 seedlings
Person Last Name	León
Person First Name	José
E-mail	jleon@ibmcp.upv.es
Telephone	(+)3496387782
Affiliation & Address	IBMCP (CSIC-UPV), CPI Edificio 8E, Ingeniero Fausto Elio s/n, 46022 Valencia (Spain)
Person role	Investigator, submitter
Replicate types	3 independent biological replicates per genotype
Experiment description	1. Type of experiment:<br>Compared analysis of the transcriptomes of 12-day old seedlings from wild type Col-0 treated with NO for 15, 30 and 60 min vs untreated control seedlings.
	2. Experimental factors:<br>Samples were harvested 12 h after dawn of day 12 after sowing and seedlings were grown under long days (16 h light / 8 h darkness) photoperiodic conditions.
	3. Number of hybridizations to Arabidopsis Nimblegen-Roche microarrays (GPL13970):<br>12 distributed as 3 independent biological replicates of untreated, NO-treated 15 min, NO-treated 30 min and NO-treated 60 min.
	4. Goals of proposed experiments:<br>Identification of the differential transcriptome affected by a pulse of NO.

Sample and Data Relationship Format (SDRF)

1. Hybridization design:				
**#**	**Label**	**Genotype**	**Growth conditions**	**Tissue**
1	Réplica 1 Col0	Col-0	MS media plus 1 % sucrose, 12 days	Seedlings
2	Réplica 1 Col0 (NO) 15 min	Col-0	MS media plus 1 % sucrose, 12 days	Seedlings
3	Réplica 1 Col0 (NO) 30 min	Col-0	MS media plus 1 % sucrose, 12 days	Seedlings
4	Réplica 1 Col0 (NO) 60 min	Col-0	MS media plus 1 % sucrose, 12 days	Seedlings
5	Réplica 2 Col0	Col-0	MS media plus 1 % sucrose, 12 days	Seedlings
6	Réplica 2 Col0 (NO) 15 min	Col-0	MS media plus 1 % sucrose, 12 days	Seedlings
7	Réplica 2 Col0 (NO) 30 min	Col-0	MS media plus 1 % sucrose, 12 days	Seedlings
8	Réplica 2 Col0 (NO) 60 min	Col-0	MS media plus 1 % sucrose, 12 days	Seedlings

9	Réplica 3 Col0	Col-0	MS media plus 1 % sucrose, 12 days	Seedlings
10	Réplica 3 Col0 (NO) 15 min	Col-0	MS media plus 1 % sucrose, 12 days	Seedlings
11	Réplica 3 Col0 (NO) 30 min	Col-0	MS media plus 1 % sucrose, 12 days	Seedlings
12	Réplica 3 Col0 (NO) 60 min	Col-0	MS media plus 1 % sucrose, 12 days	Seedlings

2. RNA extraction:
Total RNA from wild type and NO-deficient plants was isolated and purified by the Micro-to-Midi Total RNA Purification System from Invitrogen (Carlsbad, CA, USA).

3. Quality controls:
RNAs from every genotype were checked by RT-PCR for mRNA levels of the *AHB1/GLB1 and ACT2* genes. Moreover, total RNAs used for further preparation of hybridization probes were analysed to check integrity and purity by nanocapìlar electrophoresis in Bioanalyzer Agilent 2100.

4. Labeling and hybridization protocols:

RNAs were ligated to an RNA oligonucleotide adaptor (Invitrogen) using T4 RNA ligase (Ambion, http://www.ambion.com/). The RNAs were extracted once with phenol-chloroform and non-ligated adapter was removed by chromatography with MicroSpin S-300 HR columns (GE Healthcare, http://www.gehealthcare.com/). Purified ligation products were precipitated in ethanol and used as templates for reverse transcription with Superscript III (Invitrogen) for 3 h at 46°C, using oligonucleotide oligo(dT) as primer. Template RNA was removed by alkaline hydrolysis and first-strand cDNA purified with S.N.A.P. columns (Invitrogen). Second-strand synthesis was performed with Taq DNA polymerase (Roche, http://www.roche.com/) for 5 min at 94°C, 5 min; 58°C, 1 min; 72°C, 10 min. A forward oligonucleotide T7-Adap primer, which was complementary to the RNA adapter and contained the sequence of bacteriophage T7 promoter, was used. Double-stranded cDNA was then purified with MinElute columns (Qiagen) and in vitro transcribed with T7 RNA polymerase, using a MessageAmp aRNA kit (Ambion). Amplified RNA was treated with DNase I (Roche) to remove cDNA templates, purified with an aRNA Purification Module (Ambion) and then used as template for single-stranded cDNA synthesis, according to Affymetrix instructions (http://www.affymetrix.com/) as dolows: aRNA was reverse transcribed with SuperScript II (Invitrogen) for 1 h at 42°C with oligo(dT) as primer. After alkaline hydrolysis of aRNA and purification (MinElute columns, Qiagen), cDNA was fragmented with 1.5 units of DNase I (GE Healthcare) into fragments in the 50–200 bp range. Finally, 3' ends of fragmented cDNA were biotin-ddUTP labeled with terminal deoxynucleotidyl transferase (Promega, http://www.promega.com/) and GeneChip DNA labeling reagent (Affymetrix).

Three biological replicates and their corresponding negative controls were independently hybridized to ATH1 microarrays (Affymetrix), containing 22 500 transcript variants from 24 000 well-characterized Arabidopsis genes. Each sample was added to a hybridization solution containing 100 mm 2-(N-morpholino) ethanesulfonic acid, 1 m Na+, and 20 mm of EDTA in the presence of 0.01% Tween-20. Hybridization was performed for 16 h at 45°C. Each microarray was washed and stained with streptavidin–phycoerythrin in a Fluidics station 450 (Affymetrix) and scanned at 2.5-μm resolution in a GeneChip® Scanner 3000 7G system (Affymetrix). Data analyses were performed using genechip operating software (GCOS), to generate the corresponding CEL files.

Three biological replicates and their corresponding negative controls were independently hybridized to ATH1 microarrays (Affymetrix), containing 22 500 transcript variants from 24 000 well-characterized Arabidopsis genes. Each sample was added to a hybridization solution containing 100 mm 2-(N-morpholino) ethanesulfonic acid, 1 m Na+, and 20 mm of EDTA in the presence of 0.01% Tween-20. Hybridization was performed for 16 h at 45°C. Each microarray was washed and stained with streptavidin–phycoerythrin in a Fluidics station 450 (Affymetrix) and scanned at 2.5-μm resolution in a GeneChip® Scanner 3000 7G system (Affymetrix). Data analyses were performed using genechip operating software (GCOS), to generate the corresponding CEL files.

5. Sample comparisons:

NO(15 min) vs Untreated plants, NO(30 min) vs Untreated plants and NO(60 min) vs Untreated plants.

6. Statistical analysis:

Linear model methods (LiMMA) were used for determining differentially expressed genes. To control the false-discovery rate, P-values were corrected using the method of Benjamini and Hochberg (1995). Criteria for selection of genes were fold value >1.5 and false-discovery rate ≤ 0.05. Statistical analysis and graphical visualization of data were performed with the interactive tool fiesta (http://bioinfogp.cnb.csic.es/tools/FIESTA/).

Supplemental Table S2. Microarray analyses for identifying the early NO-responsive transcriptome (30 min after exposure to NO).

Fold Change	logSignal (A)	logRatio (M)	logControl	FDR (limma)	ProbeID	GENE_INFO
6.44	13.28	2.69	11.93	0.04642283	AT5G66210	CPK28__calcium-dependent protein kinase 28
6.22	11.83	2.64	10.51	0.04658549	AT1G63940	MDAR6__monodehydroascorbate reductase 6
5.3	12.82	2.4	11.62	0.0493034	AT5G45340	CYP707A3__cytochrome P450, family 707, subfamily A, polypeptide 3CYP707A3__cytochrome P450, family 707, subfamily A, polypeptide 3
5.18	8.94	2.37	7.75	0.03000485	AT5G64130	cAMP-regulated phosphoprotein 19-related protein
5.01	12.99	2.33	11.83	0.04789237	AT5G45340	CYP707A3__cytochrome P450, family 707, subfamily A, polypeptide 3CYP707A3__cytochrome P450, family 707, subfamily A, polypeptide 3
4.94	13.11	2.31	11.96	0.04642283	AT5G10210	
4.89	13.56	2.29	12.42	0.04015667	AT5G51190	ERF105__Integrase-type DNA-binding superfamily protein
4.87	10.79	2.28	9.64	0.04658549	AT5G28610	
4.2	12.01	2.07	10.97	0.03731617	AT3G23250	ATMYB15_ATY19_MYB15__myb domain protein 15ATMYB15_ATY19_MYB15__myb domain protein 15
4.08	13.3	2.03	12.28	0.04658549	AT5G47230	ATERF-5_ATERF5_AtMACD1_ERF102_ERF5__ethylene responsive element binding factor 5
4.05	12	2.02	10.99	0.04642283	AT3G23250	ATMYB15_ATY19_MYB15__myb domain protein 15ATMYB15_ATY19_MYB15__myb domain protein 15
3.46	13.8	1.79	12.9	0.03731617	AT3G50930	BCS1__cytochrome BC1 synthesis
3.25	14.43	1.7	13.58	0.03731617	AT4G29780	
3.2	12.55	1.68	11.71	0.04642283	AT1G61340	AtFBS1_FBS1__F-box family protein
3.2	14.26	1.68	13.42	0.0493034	AT4G17490	ATERF6_ERF-6-6_ERF6__ethylene responsive element binding factor 6
2.41	8.08	1.27	7.44	0.04658549	AT4G25030	
2.38	14.49	1.25	13.86	0.04789237	AT5G04340	C2H2_CZF2_ZAT6__zinc finger of Arabidopsis thaliana 6
2.13	13.04	1.09	12.49	0.0493034	AT4G27652	
2.12	9.84	1.08	9.3	0.04789237	AT5G22530	
2.1	12.94	1.07	12.4	0.04642283	AT2G39650	Protein of unknown function (DUF506)
2.06	13.18	1.05	12.65	0.04642283	AT1G17147	VQ motif-containing protein
2.04	12.46	1.03	11.94	0.04789237	AT5G47960	ATRABA4C_RABA4C_SMG1__RAB GTPase homolog A4C
1.79	10.65	0.84	10.23	0.0493034	AT5G26038	MIR860A__MIR860a; miRNA
1.75	8.76	0.8	8.36	0.0493034	AT5G41080	AtGDPD2_GDPD2__PLC-like phosphodiesterases superfamily protein
-2.04	10.77	-1.03	11.28	0.04789237	AT1G60190	AtPUB19_PUB19__ARM repeat superfamily protein
-2.04	11.86	-1.03	12.37	0.04789237	AT5G61350	CAP1__Protein kinase superfamily protein
-2.24	8.16	-1.17	8.74	0.04789237	AT3G57810	Cysteine proteinases superfamily protein

-3.06	7.82	-1.61	8.63	0.03000485	AT1G26140	
-3.19	11.01	-1.67	11.84	0.03731617	AT1G58889	transposable element gene
-3.85	11.71	-1.95	12.69	0.04658549	AT3G21660	UBX domain-containing protein
-4.27	10.06	-2.09	11.1	0.0493034	AT1G09500	NAD(P)-binding Rossmann-fold superfamily protein

Supplemental Table S2. Microarray analyses for identifying the early NO-responsive transcriptome (60 min after exposure to NO).

Fold Change	logSignal (A)	logRatio (M)	logControl	FDR (limma)	ProbeID	GENE_INFO
25.72	11.25	4.68	8.91	0.00294186	AT1G56240	AtPP2-B13_PP2-B13__phloem protein 2-B13
17.32	11	4.11	8.94	0.00162136	AT4G34410	RRTF1__redox responsive transcription factor 1
17.29	11.17	4.11	9.12	0.00356968	AT1G56250	AtPP2-B14_PP2-B14__phloem protein 2-B14_F-box protein VBF
12.12	12.31	3.6	10.51	0.00013825	AT1G63940	MDAR6__monodehydroascorbate reductase 6
10.63	11.35	3.41	9.64	0.00195989	AT5G28610	BEST Arabidopsis thaliana protein match is: glycine-rich protein (TAIR:AT5G28630.1); Has 1536 Blast hits to 1202 proteins in 136 species: Archae - 0; Bacteria - 8; Metazoa - 888; Fungi - 120; Plants - 71; Viruses - 39; Other Eukaryotes - 410 (source: NCBI BLink).
10.06	8.67	3.33	7	0.01344817	AT3G43250	Family of unknown function (DUF572)
9.72	12.98	3.28	11.34	0.00420969	AT1G43800	Plant stearoyl-acyl-carrier-protein desaturase family protein
9.67	12	3.27	10.36	0.00162136	AT3G45960	ATEXLA3_ATEXPL3_ATHEXP BETA 2.3_EXLA3_EXPL3__expansin-like A3 ATEXLA3_ATEXPL3_ATHEXP BETA 2.3_EXLA3_EXPL3__expansin-like A3
9.3	11.41	3.22	9.8	0.0012111	AT2G44840	ATERF13_EREBP_ERF13__ethylene-responsive element binding factor 13
9.29	12.14	3.22	10.53	0.00122807	AT3G45960	ATEXLA3_ATEXPL3_ATHEXP BETA 2.3_EXLA3_EXPL3__expansin-like A3 ATEXLA3_ATEXPL3_ATHEXP BETA 2.3_EXLA3_EXPL3__expansin-like A3
9.09	11.64	3.18	10.05	0.00288203	AT5G19110	Eukaryotic aspartyl protease family protein
8.97	12.3	3.17	10.72	0.00309366	AT4G27970	SLAH2__SLAC1 homologue 2
8.52	12.52	3.09	10.97	0.00221781	AT3G23250	ATMYB15_ATY19_MYB15__myb domain protein 15 ATMYB15_ATY19_MYB15__myb domain protein 15
8.38	12.52	3.07	10.99	0.00229082	AT3G23250	ATMYB15_ATY19_MYB15__myb domain protein 15 ATMYB15_ATY19_MYB15__myb domain protein 15
8.31	11.31	3.06	9.78	0.00470122	AT1G64160	Disease resistance-responsive (dirigent-like protein) family protein
8.19	11.61	3.03	10.09	0.00394474	AT4G24110	unknown protein; Has 76 Blast hits to 76 proteins in 13 species: Archae - 0; Bacteria - 0; Metazoa - 1; Fungi - 0; Plants - 75; Viruses - 0; Other Eukaryotes - 0 (source: NCBI BLink).
8.01	8.68	3	7.18	0.00294186	AT5G40000	P-loop containing nucleoside triphosphate hydrolases superfamily protein
7.62	13.4	2.93	11.93	0.00219513	AT5G66210	CPK28__calcium-dependent protein kinase 28
7.6	12.46	2.93	11	0.00229082	AT1G11185	

7.14	10.06	2.84	8.64	0.003464	AT5G28630	glycine-rich protein
7.04	10.22	2.82	8.81	0.00692117	AT5G11140	Arabidopsis phospholipase-like protein (PEARLI 4) family
6.78	9.27	2.76	7.89	0.01984953	AT5G13220	JAS1_JAZ10_TIFY9__jasmonate-zim-domain protein 10 JAS1_JAZ10_TIFY9__jasmonate-zim-domain protein 10 JAS1_JAZ10_TIFY9__jasmonate-zim-domain protein 10 JAS1_JAZ10_TIFY9__jasmonate-zim-domain protein 10
6.76	10.43	2.76	9.05	0.00782924	AT1G71520	Integrase-type DNA-binding superfamily protein
6.59	12.86	2.72	11.5	0.00294186	AT1G76650	CML38__calmodulin-like 38 CML38__calmodulin-like 38
6.57	11.63	2.72	10.27	0.01083634	AT3G49620	DIN11__2-oxoglutarate (2OG) and Fe(II)-dependent oxygenase superfamily protein
6.49	12.06	2.7	10.72	0.003464	AT2G16060	AHB1_ARATH GLB1_ATGLB1_GLB1_HB1_NSHB1__hemoglobin 1
6.48	9.69	2.7	8.35	0.00488717	AT2G13706	
6.42	11.94	2.68	10.6	0.00830701	AT1G65390	ATPP2-A5_PP2-A5__phloem protein 2 A5 ATPP2-A5_PP2-A5__phloem protein 2 A5
6.35	10.74	2.67	9.4	0.00996592	AT3G23230	Integrase-type DNA-binding superfamily protein
6.33	9.73	2.66	8.4	0.00294186	AT1G32910	HXXXD-type acyl-transferase family protein
6.28	9.13	2.65	7.8	0.00322354	AT2G06045	
6.19	8.3	2.63	6.98	0.00179583	AT1G66860	Class I glutamine amidotransferase-like superfamily protein
6.14	11.52	2.62	10.21	0.00356968	AT2G44578	RING/U-box superfamily protein
6.12	9.06	2.61	7.75	0.00195989	AT5G64130	cAMP-regulated phosphoprotein 19-related protein cAMP-regulated phosphoprotein 19-related protein
6.05	12.92	2.6	11.62	0.0021898	AT5G45340	CYP707A3__cytochrome P450, family 707, subfamily A, polypeptide 3 CYP707A3__cytochrome P450, family 707, subfamily A, polypeptide 3
5.96	12.56	2.57	11.27	0.00309366	AT3G29000	Calcium-binding EF-hand family protein
5.92	9.41	2.56	8.12	0.02504352	AT3G12910	NAC (No Apical Meristem) domain transcriptional regulator superfamily protein
5.87	13.24	2.55	11.96	0.0012111	AT5G10210	CONTAINS InterPro DOMAIN/s: C2 calcium-dependent membrane targeting (InterPro:IPR000008); BEST Arabidopsis thaliana protein match is: unknown protein (TAIR:AT5G65030.1); Has 1807 Blast hits to 1807 proteins in 277 species: Archae - 0; Bacteria - 0; Metazoa - 736; Fungi - 347; Plants - 385; Viruses - 0; Other Eukaryotes - 339 (source: NCBI BLink).
5.86	11.66	2.55	10.38	0.00050433	AT1G14480	Ankyrin repeat family protein
5.83	11.9	2.54	10.63	0.00784132	AT1G65390	ATPP2-A5_PP2-A5__phloem protein 2 A5 ATPP2-A5_PP2-A5__phloem protein 2 A5
5.8	11.9	2.54	10.63	0.00294186	AT1G15040	Class I glutamine amidotransferase-like superfamily protein Class I glutamine amidotransferase-like superfamily protein
5.8	12.54	2.54	11.27	0.00356968	AT1G76650	CML38__calmodulin-like 38 CML38__calmodulin-like 38
5.59	11.91	2.48	10.67	0.00574644	AT4G15975	RING/U-box superfamily protein
5.56	9.16	2.48	7.92	0.00326244	AT3G10930	unknown protein; FUNCTIONS IN: molecular_function unknown; INVOLVED IN: biological_process unknown; LOCATED IN: mitochondrion; EXPRESSED IN: 18 plant structures; EXPRESSED DURING: 9 growth stages; Has 1 Blast hits to 1 proteins in 1 species:

						Archae - 0; Bacteria - 0; Metazoa - 0; Fungi - 0; Plants - 1; Viruses - 0; Other Eukaryotes - 0 (source: NCBI BLink).
5.45	11.62	2.45	10.39	0.0049944	AT5G15120	Plant Cysteine Oxidase 1 (PCO1)
5.43	9.62	2.44	8.4	0.00410995	AT3G14260	Protein of unknown function (DUF567)
5.35	10.86	2.42	9.65	0.00429467	AT5G67080	MAPKKK19__mitogen-activated protein kinase kinase kinase 19
5.31	13.03	2.41	11.83	0.00266801	AT5G45340	CYP707A3__cytochrome P450, family 707, subfamily A, polypeptide 3 CYP707A3__cytochrome P450, family 707, subfamily A, polypeptide 3
5.31	13.62	2.41	12.42	0.0026004	AT5G51190	Integrase-type DNA-binding superfamily protein
5.18	11.93	2.37	10.74	0.00514581	AT2G22880	VQ motif-containing protein
5.1	8.74	2.35	7.57	0.01873743	AT1G69930	ATGSTU11_GSTU11__glutathione S-transferase TAU 11
4.96	12.69	2.31	11.54	0.00294186	AT1G05575	unknown protein; FUNCTIONS IN: molecular_function unknown; INVOLVED IN: anaerobic respiration; LOCATED IN: endomembrane system; EXPRESSED IN: 17 plant structures; EXPRESSED DURING: 9 growth stages; BEST Arabidopsis thaliana protein match is: unknown protein (TAIR:AT2G31945.1); Has 63 Blast hits to 63 proteins in 10 species: Archae - 0; Bacteria - 0; Metazoa - 0; Fungi - 0; Plants - 63; Viruses - 0; Other Eukaryotes - 0 (source: NCBI BLink).
4.91	8.69	2.3	7.55	0.00461806	AT1G22810	Integrase-type DNA-binding superfamily protein
4.81	11.93	2.27	10.79	0.00250146	AT1G30370	DLAH__alpha/beta-Hydrolases superfamily protein
4.8	13.06	2.26	11.93	0.00229082	AT5G13220	JAS1_JAZ10_TIFY9__jasmonate-zim-domain protein 10 JAS1_JAZ10_TIFY9__jasmonate-zim-domain protein 10 JAS1_JAZ10_TIFY9__jasmonate-zim-domain protein 10 JAS1_JAZ10_TIFY9__jasmonate-zim-domain protein 10
4.79	12.09	2.26	10.96	0.00309366	AT2G18210	unknown protein; BEST Arabidopsis thaliana protein match is: unknown protein (TAIR:AT4G36500.1); Has 50 Blast hits to 50 proteins in 7 species: Archae - 0; Bacteria - 0; Metazoa - 0; Fungi - 0; Plants - 50; Viruses - 0; Other Eukaryotes - 0 (source: NCBI BLink).
4.67	13.27	2.22	12.16	0.00254885	AT5G13220	JAS1_JAZ10_TIFY9__jasmonate-zim-domain protein 10 JAS1_JAZ10_TIFY9__jasmonate-zim-domain protein 10 JAS1_JAZ10_TIFY9__jasmonate-zim-domain protein 10 JAS1_JAZ10_TIFY9__jasmonate-zim-domain protein 10
4.65	10.4	2.22	9.3	0.00542693	AT5G22530	unknown protein; BEST Arabidopsis thaliana protein match is: unknown protein (TAIR:AT5G22520.1); Has 30201 Blast hits to 17322 proteins in 780 species: Archae - 12; Bacteria - 1396; Metazoa - 17338; Fungi - 3422; Plants - 5037; Viruses - 0; Other Eukaryotes - 2996 (source: NCBI BLink).
4.5	13.24	2.17	12.16	0.003464	AT2G24600	Ankyrin repeat family protein Ankyrin repeat family protein Ankyrin repeat family protein
4.49	9.03	2.17	7.95	0.0052996	AT1G43040	SAUR-like auxin-responsive protein family
4.37	12.44	2.13	11.38	0.00583609	AT1G21326	VQ motif-containing protein
4.36	12.88	2.12	11.82	0.00603875	AT3G54150	S-adenosyl-L-methionine-dependent methyltransferases superfamily protein
4.36	13.06	2.13	12	0.0031719	AT4G21390	B120__S-locus lectin protein kinase family protein
4.36	7.52	2.12	6.45	0.00323071	AT4G27654	unknown protein; FUNCTIONS IN: molecular_function unknown; INVOLVED IN:

						biological_process unknown; LOCATED IN: endomembrane system; EXPRESSED IN: 17 plant structures; EXPRESSED DURING: 9 growth stages; Has 30201 Blast hits to 17322 proteins in 780 species: Archae - 12; Bacteria - 1396; Metazoa - 17338; Fungi - 3422; Plants - 5037; Viruses - 0; Other Eukaryotes - 2996 (source: NCBI BLink).
4.25	13.6	2.09	12.55	0.00294186	AT2G24600	Ankyrin repeat family protein Ankyrin repeat family protein Ankyrin repeat family protein
4.24	13.32	2.08	12.28	0.0029534	AT5G47230	ATERF-5_ATERF5_ERF5__ethylene responsive element binding factor 5
4.23	12.4	2.08	11.36	0.00294186	AT2G32030	Acyl-CoA N-acyltransferases (NAT) superfamily protein
4.22	12.78	2.08	11.74	0.00309366	AT1G17380	JAZ5_TIFY11A__jasmonate-zim-domain protein 5
4.22	13.05	2.08	12.02	0.00310291	AT3G25780	AOC3__allene oxide cyclase 3
4.11	13.92	2.04	12.9	0.00229082	AT3G50930	BCS1__cytochrome BC1 synthesis
4.1	9.92	2.03	8.9	0.01056861	AT2G47950	unknown protein; FUNCTIONS IN: molecular_function unknown; INVOLVED IN: biological_process unknown; LOCATED IN: endomembrane system; EXPRESSED IN: root, flower; EXPRESSED DURING: petal differentiation and expansion stage; BEST Arabidopsis thaliana protein match is: unknown protein (TAIR:AT3G62990.1); Has 22 Blast hits to 22 proteins in 5 species: Archae - 0; Bacteria - 0; Metazoa - 0; Fungi - 0; Plants - 22; Viruses - 0; Other Eukaryotes - 0 (source: NCBI BLink).
3.99	12.71	2	11.71	0.00162136	AT1G61340	F-box family protein
3.95	13.66	1.98	12.67	0.00417546	AT1G19530	unknown protein; FUNCTIONS IN: molecular_function unknown; INVOLVED IN: N-terminal protein myristoylation, anaerobic respiration; LOCATED IN: cellular_component unknown; EXPRESSED IN: leaf apex, inflorescence meristem, hypocotyl, root, flower; EXPRESSED DURING: petal differentiation and expansion stage; Has 47 Blast hits to 47 proteins in 13 species: Archae - 0; Bacteria - 0; Metazoa - 0; Fungi - 0; Plants - 47; Viruses - 0; Other Eukaryotes - 0 (source: NCBI BLink).
3.95	13.45	1.98	12.46	0.0062763	AT4G21680	NRT1.8__NITRATE TRANSPORTER 1.8
3.88	9.22	1.96	8.24	0.01033062	AT1G19210	Integrase-type DNA-binding superfamily protein
3.88	9.3	1.96	8.32	0.0059133	AT2G37430	C2H2 and C2HC zinc fingers superfamily protein
3.87	11.74	1.95	10.76	0.007271	AT5G64750	ABR1__Integrase-type DNA-binding superfamily protein
3.86	12.53	1.95	11.56	0.00610609	AT4G13395	DVL10_RTFL12__ROTUNDIFOLIA like 12
3.86	12.75	1.95	11.77	0.00402989	AT5G14700	NAD(P)-binding Rossmann-fold superfamily protein
3.83	7.61	1.94	6.64	0.01039189	AT5G21960	Integrase-type DNA-binding superfamily protein
3.83	10.47	1.94	9.5	0.00539566	AT5G46295	unknown protein; BEST Arabidopsis thaliana protein match is: unknown protein (TAIR:AT1G06475.1); Has 30201 Blast hits to 17322 proteins in 780 species: Archae - 12; Bacteria - 1396; Metazoa - 17338; Fungi - 3422; Plants - 5037; Viruses - 0; Other Eukaryotes - 2996 (source: NCBI BLink).
3.75	13.8	1.91	12.85	0.00306866	AT1G72520	ATLOX4_LOX4__PLAT/LH2 domain-containing lipoxygenase family protein
3.75	13.09	1.91	12.14	0.00195989	AT3G59480	pfkB-like carbohydrate kinase family protein

3.74	14.09	1.9	13.14	0.00309366	AT1G14040	EXS (ERD1/XPR1/SYG1) family protein
3.74	10.29	1.9	9.34	0.01218332	AT4G10270	Wound-responsive family protein
3.73	13.69	1.9	12.74	0.00294186	AT1G28370	ATERF11_ERF11__ERF domain protein 11
3.71	10.99	1.89	10.05	0.00356968	AT3G04220	Disease resistance protein (TIR-NBS-LRR class) family
3.69	10.77	1.88	9.83	0.02313154	AT1G77640	Integrase-type DNA-binding superfamily protein
3.69	14.3	1.89	13.35	0.00102599	AT4G31800	ATWRKY18_WRKY18__WRKY DNA-binding protein 18
3.68	14.52	1.88	13.58	0.00102599	AT4G29780	unknown protein; BEST Arabidopsis thaliana protein match is: unknown protein (TAIR:AT5G12010.1); Has 945 Blast hits to 944 proteins in 87 species: Archae - 0; Bacteria - 0; Metazoa - 519; Fungi - 43; Plants - 365; Viruses - 0; Other Eukaryotes - 18 (source: NCBI BLink).
3.67	11.37	1.88	10.44	0.00518233	AT2G47890	BBX11_B-box type zinc finger protein with CCT domain
3.58	11.71	1.84	10.79	0.01399612	AT5G39890	Plant Cysteine Oxidase 2 (PCO2)
3.56	13.19	1.83	12.27	0.00196725	AT5G54490	PBP1__pinoid-binding protein 1
3.51	11.37	1.81	10.46	0.0031892	AT5G58680	ARM repeat superfamily protein
3.49	10.11	1.8	9.21	0.00326047	AT5G64905	PROPEP3__elicitor peptide 3 precursor
3.45	14.08	1.79	13.19	0.0172413	AT5G39580	Peroxidase superfamily protein Peroxidase superfamily protein
3.44	14.31	1.78	13.42	0.00221781	AT4G17490	ATERF6_ERF-6-6_ERF6__ethylene responsive element binding factor 6
3.43	11.37	1.78	10.48	0.00251625	AT3G55840	Hs1pro-1 protein
3.42	6.95	1.77	6.06	0.01354972	AT1G12805	nucleotide binding
3.41	12.62	1.77	11.73	0.00372592	AT4G28085	unknown protein; Has 35333 Blast hits to 34131 proteins in 2444 species: Archae - 798; Bacteria - 22429; Metazoa - 974; Fungi - 991; Plants - 531; Viruses - 0; Other Eukaryotes - 9610 (source: NCBI BLink).
3.39	11.72	1.76	10.84	0.01256559	AT5G23510	unknown protein; LOCATED IN: cellular_component unknown; BEST Arabidopsis thaliana protein match is: unknown protein (TAIR:AT5G23490.1); Has 30201 Blast hits to 17322 proteins in 780 species: Archae - 12; Bacteria - 1396; Metazoa - 17338; Fungi - 3422; Plants - 5037; Viruses - 0; Other Eukaryotes - 2996 (source: NCBI BLink).
3.38	13.96	1.76	13.08	0.0182418	AT5G39580	Peroxidase superfamily protein Peroxidase superfamily protein
3.37	7.96	1.75	7.08	0.01103668	AT4G10265	Wound-responsive family protein
3.36	11.19	1.75	10.32	0.00402989	AT3G60550	CYCP3;2__cyclin p3;2
3.36	9.59	1.75	8.72	0.01476349	AT4G11470	CRK31__cysteine-rich RLK (RECEPTOR-like protein kinase) 31
3.36	10.68	1.75	9.81	0.00929952	AT4G14450	unknown protein; LOCATED IN: chloroplast; BEST Arabidopsis thaliana protein match is: unknown protein (TAIR:AT3G23170.1); Has 74 Blast hits to 74 proteins in 6 species: Archae - 0; Bacteria - 0; Metazoa - 0; Fungi - 0; Plants - 74; Viruses - 0; Other Eukaryotes - 0 (source: NCBI BLink).
3.34	14.05	1.74	13.18	0.00294186	AT1G69890	Protein of unknown function (DUF569)
3.32	10.37	1.73	9.5	0.00710216	AT4G21920	unknown protein; FUNCTIONS IN: molecular_function unknown; INVOLVED IN: N-terminal protein myristoylation; LOCATED IN: cellular_component unknown; BEST Arabidopsis thaliana

						protein match is: unknown protein (TAIR:AT3G20340.1); Has 40 Blast hits to 40 proteins in 10 species: Archae - 0; Bacteria - 0; Metazoa - 0; Fungi - 0; Plants - 40; Viruses - 0; Other Eukaryotes - 0 (source: NCBI BLink).
3.28	13.68	1.71	12.82	0.00394474	AT3G02550	LBD41__LOB domain-containing protein 41
3.28	10.48	1.72	9.62	0.00953954	AT3G23240	ATERF1_ERF1__ethylene response factor 1
3.28	11.49	1.71	10.63	0.00429467	AT5G42380	CML37_CML39__calmodulin like 37
3.26	10.35	1.7	9.5	0.00836735	AT3G28580	P-loop containing nucleoside triphosphate hydrolases superfamily protein
3.25	13.19	1.7	12.34	0.00195989	AT1G28480	GRX480_roxy19__Thioredoxin superfamily protein
3.25	8.91	1.7	8.06	0.01508049	AT5G17430	BBM__Integrase-type DNA-binding superfamily protein
3.24	12.1	1.69	11.25	0.00309366	AT3G25250	AGC2_AGC2-1_AtOXI1_OXI1__AGC (cAMP-dependent, cGMP-dependent and protein kinase C) kinase family protein
3.22	11.4	1.69	10.56	0.01357873	AT2G27690	CYP94C1__cytochrome P450, family 94, subfamily C, polypeptide 1
3.21	12.13	1.68	11.29	0.00495528	AT3G25790	HHO1, HRS1 Homologue 1_myb-like transcription factor family protein
3.2	7.66	1.68	6.81	0.00420934	AT1G70130	Concanavalin A-like lectin protein kinase family protein
3.18	9.63	1.67	8.8	0.01676505	AT3G02840	ARM repeat superfamily protein
3.15	10.01	1.66	9.18	0.00229082	AT1G50750	Plant mobile domain protein family
3.15	12.75	1.66	11.92	0.00420866	AT2G24600	Ankyrin repeat family protein Ankyrin repeat family protein Ankyrin repeat family protein
3.14	13.6	1.65	12.78	0.00309366	AT1G78050	PGM__phosphoglycerate/bisphosphoglycerate mutase
3.13	7.86	1.65	7.04	0.0165446	AT1G76640	Calcium-binding EF-hand family protein
3.13	13.26	1.64	12.44	0.00306866	AT4G18340	Glycosyl hydrolase superfamily protein
3.08	12.79	1.62	11.98	0.0029534	AT2G35710	Nucleotide-diphospho-sugar transferases superfamily protein Nucleotide-diphospho-sugar transferases superfamily protein
3.07	14.34	1.62	13.53	0.00309366	AT1G80840	ATWRKY40_WRKY40__WRKY DNA-binding protein 40
3.03	11.85	1.6	11.05	0.00309366	AT3G10040	sequence-specific DNA binding transcription factors
3.03	9.6	1.6	8.8	0.0151051	AT5G59570	BOA__Homeodomain-like superfamily protein
3.02	10.7	1.59	9.9	0.01708562	AT4G37670	NAGS2__N-acetyl-l-glutamate synthase 2
2.99	13.87	1.58	13.08	0.00356968	AT1G17750	AtPEPR2_PEPR2__PEP1 receptor 2
2.96	10.8	1.56	10.02	0.01506067	AT5G22520	unknown protein; BEST Arabidopsis thaliana protein match is: unknown protein (TAIR:AT5G22530.1); Has 14 Blast hits to 14 proteins in 2 species: Archae - 0; Bacteria - 0; Metazoa - 0; Fungi - 0; Plants - 14; Viruses - 0; Other Eukaryotes - 0 (source: NCBI BLink).
2.94	13.72	1.56	12.94	0.00382427	AT3G44260	AtCAF1a_CAF1a__Polynucleotidyl transferase, ribonuclease H-like superfamily protein
2.92	12.68	1.54	11.91	0.00356968	AT3G11840	PUB24__plant U-box 24
2.92	11.48	1.55	10.71	0.0385492	AT4G35180	LHT7__LYS/HIS transporter 7
2.91	13.2	1.54	12.43	0.00950083	AT5G62340	Plant invertase/pectin methylesterase inhibitor superfamily protein
2.9	11.55	1.54	10.78	0.01874475	AT4G26200	ACS7_ATACS7__1-amino-cyclopropane-1-carboxylate synthase 7
2.9	11.17	1.53	10.41	0.00394838	AT5G27420	ATL31_CNI1__carbon/nitrogen insensitive 1

2.87	10.14	1.52	9.38	0.00294186	AT3G06435	Expressed protein
2.87	8.73	1.52	7.97	0.0134152	AT5G19097	
2.85	11.36	1.51	10.61	0.00904592	AT5G65300	unknown protein; Has 30201 Blast hits to 17322 proteins in 780 species: Archae - 12; Bacteria - 1396; Metazoa - 17338; Fungi - 3422; Plants - 5037; Viruses - 0; Other Eukaryotes - 2996 (source: NCBI BLink).
2.83	12.49	1.5	11.74	0.00486057	AT1G80820	ATCCR2_CCR2__cinnamoyl coa reductase
2.83	14.62	1.5	13.86	0.00290947	AT5G04340	C2H2_CZF2_ZAT6__zinc finger of Arabidopsis thaliana 6
2.82	9.79	1.5	9.04	0.00927726	AT1G72416	Chaperone DnaJ-domain superfamily protein Chaperone DnaJ-domain superfamily protein Chaperone DnaJ-domain superfamily protein Chaperone DnaJ-domain superfamily protein
2.81	13.27	1.49	12.52	0.01044382	AT1G19380	Protein of unknown function (DUF1195)
2.81	10.64	1.49	9.9	0.00269259	AT5G58720	smr (Small MutS Related) domain-containing protein
2.81	11.68	1.49	10.94	0.00201069	AT5G66070	RING/U-box superfamily protein
2.78	14.34	1.48	13.61	0.00306866	AT1G18570	AtMYB51_BW51A_BW51B_HIG1_MYB51__myb domain protein 51
2.78	12.05	1.48	11.31	0.00264502	AT2G01300	unknown protein; BEST Arabidopsis thaliana protein match is: unknown protein (TAIR:AT1G15010.1); Has 73 Blast hits to 73 proteins in 12 species: Archae - 0; Bacteria - 0; Metazoa - 0; Fungi - 0; Plants - 73; Viruses - 0; Other Eukaryotes - 0 (source: NCBI BLink).
2.78	13.41	1.47	12.68	0.00309366	AT5G66650	Protein of unknown function (DUF607)
2.77	12.62	1.47	11.88	0.0332894	AT3G26200	CYP71B22__cytochrome P450, family 71, subfamily B, polypeptide 22
2.75	12.84	1.46	12.11	0.00250146	AT3G43430	RING/U-box superfamily protein
2.74	14.52	1.45	13.79	0.00326511	AT1G27730	STZ_ZAT10__salt tolerance zinc finger
2.74	14.57	1.45	13.84	0.00358217	AT4G25810	XTH23_XTR6__xyloglucan endotransglycosylase 6
2.73	10.38	1.45	9.66	0.00449194	AT3G06490	AtMYB108_BOS1_MYB108__myb domain protein 108
2.72	11.03	1.45	10.31	0.00825991	AT1G56680	Chitinase family protein
2.72	9.45	1.44	8.73	0.00294186	AT1G58420	Uncharacterised conserved protein UCP031279
2.71	12.35	1.44	11.63	0.00392318	AT4G28703	RmlC-like cupins superfamily protein
2.69	12.25	1.43	11.53	0.00451369	AT2G35710	Nucleotide-diphospho-sugar transferases superfamily protein Nucleotide-diphospho-sugar transferases superfamily protein
2.68	11.14	1.42	10.42	0.00420969	AT1G79310	AtMC7_AtMCP2a_MC7_MCP2a__metacaspase 7
2.68	13.1	1.42	12.39	0.00610609	AT4G20780	CML42__calmodulin like 42
2.68	13.86	1.42	13.15	0.00479295	AT5G41080	AtGDPD2_GDPD2__PLC-like phosphodiesterases superfamily protein AtGDPD2_GDPD2__PLC-like phosphodiesterases superfamily protein
2.67	10.33	1.42	9.62	0.01622576	AT2G12646	PLATZ transcription factor family protein
2.67	10.51	1.42	9.8	0.0079029	AT2G27080	Late embryogenesis abundant (LEA) hydroxyproline-rich glycoprotein family
2.67	10.98	1.41	10.27	0.00426996	AT5G64130	cAMP-regulated phosphoprotein 19-related protein cAMP-regulated phosphoprotein 19-related protein
2.66	11.9	1.41	11.19	0.00426578	AT5G01100	O-fucosyltransferase family protein

2.66	13.43	1.41	12.73	0.00309366	AT5G07440	GDH2__glutamate dehydrogenase 2
2.66	12.91	1.41	12.2	0.01481975	AT5G10130	Pollen Ole e 1 allergen and extensin family protein
2.65	13.5	1.41	12.79	0.01264106	AT1G10990	unknown protein; Has 6 Blast hits to 6 proteins in 2 species: Archae - 0; Bacteria - 0; Metazoa - 0; Fungi - 0; Plants - 6; Viruses - 0; Other Eukaryotes - 0 (source: NCBI BLink). unknown protein; Has 6 Blast hits to 6 proteins in 2 species: Archae - 0; Bacteria - 0; Metazoa - 0; Fungi - 0; Plants - 6; Viruses - 0; Other Eukaryotes - 0 (source: NCBI BLink).
2.65	9.13	1.41	8.43	0.00622149	AT1G15330	Cystathionine beta-synthase (CBS) protein
2.65	13.19	1.41	12.49	0.00229082	AT4G27652	unknown protein; BEST Arabidopsis thaliana protein match is: unknown protein (TAIR:AT4G27657.1); Has 30201 Blast hits to 17322 proteins in 780 species: Archae - 12; Bacteria - 1396; Metazoa - 17338; Fungi - 3422; Plants - 5037; Viruses - 0; Other Eukaryotes - 2996 (source: NCBI BLink).
2.64	14.2	1.4	13.5	0.00481576	AT1G15040	Class I glutamine amidotransferase-like superfamily protein Class I glutamine amidotransferase-like superfamily protein
2.64	14.16	1.4	13.46	0.00565467	AT5G57220	CYP81F2__cytochrome P450, family 81, subfamily F, polypeptide 2
2.63	13.64	1.39	12.94	0.00740087	AT1G72416	Chaperone DnaJ-domain superfamily protein Chaperone DnaJ-domain superfamily protein Chaperone DnaJ-domain superfamily protein Chaperone DnaJ-domain superfamily protein
2.63	11.76	1.4	11.06	0.01051861	AT5G65600	Concanavalin A-like lectin protein kinase family protein
2.62	12.67	1.39	11.97	0.01661574	AT1G10990	unknown protein; Has 6 Blast hits to 6 proteins in 2 species: Archae - 0; Bacteria - 0; Metazoa - 0; Fungi - 0; Plants - 6; Viruses - 0; Other Eukaryotes - 0 (source: NCBI BLink). unknown protein; Has 6 Blast hits to 6 proteins in 2 species: Archae - 0; Bacteria - 0; Metazoa - 0; Fungi - 0; Plants - 6; Viruses - 0; Other Eukaryotes - 0 (source: NCBI BLink).
2.62	11.88	1.39	11.19	0.00338136	AT4G23810	ATWRKY53_WRKY53__WRKY family transcription factor
2.62	12.29	1.39	11.6	0.0040103	AT5G48110	Terpenoid cyclases/Protein prenyltransferases superfamily protein
2.61	13.37	1.38	12.68	0.00422741	AT2G25735	unknown protein; Has 31 Blast hits to 31 proteins in 9 species: Archae - 0; Bacteria - 0; Metazoa - 0; Fungi - 0; Plants - 31; Viruses - 0; Other Eukaryotes - 0 (source: NCBI BLink).
2.6	12.1	1.38	11.41	0.01241051	AT4G08345	
2.59	12.05	1.38	11.37	0.00676276	AT1G21320	nucleotide binding;nucleic acid binding
2.59	8.15	1.38	7.47	0.01812225	AT2G14290	F-box family protein with a domain of unknown function (DUF295)
2.59	12.47	1.38	11.78	0.00459015	AT2G41800	Protein of unknown function, DUF642
2.58	14.36	1.37	13.67	0.00286412	AT3G57450	unknown protein; Has 65 Blast hits to 65 proteins in 11 species: Archae - 0; Bacteria - 0; Metazoa - 0; Fungi - 0; Plants - 65; Viruses - 0; Other Eukaryotes - 0 (source: NCBI BLink).
2.58	10.53	1.36	9.85	0.00426996	AT4G28930	unknown protein; FUNCTIONS IN: molecular_function unknown; INVOLVED IN: biological_process unknown; LOCATED IN: endomembrane system; BEST Arabidopsis thaliana protein match is: unknown protein (TAIR:AT4G12930.1); Has 12 Blast hits to 12 proteins in 2 species: Archae - 0; Bacteria - 0; Metazoa - 0; Fungi - 0; Plants - 12; Viruses - 0; Other Eukaryotes - 0 (source: NCBI BLink).

2.58	9.04	1.37	8.36	0.00306866	AT5G41080	AtGDPD2_GDPD2__PLC-like phosphodiesterases superfamily protein AtGDPD2_GDPD2__PLC-like phosphodiesterases superfamily protein
2.57	9.86	1.36	9.18	0.00638451	AT1G35210	unknown protein; FUNCTIONS IN: molecular_function unknown; INVOLVED IN: biological_process unknown; LOCATED IN: chloroplast; EXPRESSED IN: 14 plant structures; EXPRESSED DURING: 9 growth stages; CONTAINS InterPro DOMAIN/s: Protein of unknown function DUF740 (InterPro:IPR008004); BEST Arabidopsis thaliana protein match is: unknown protein (TAIR:AT1G22470.1); Has 83 Blast hits to 83 proteins in 12 species: Archae - 0; Bacteria - 0; Metazoa - 0; Fungi - 0; Plants - 81; Viruses - 0; Other Eukaryotes - 2 (source: NCBI BLink).
2.57	11.9	1.36	11.22	0.00356968	AT4G39580	Galactose oxidase/kelch repeat superfamily protein
2.56	9.09	1.36	8.41	0.01899215	AT1G66600	ABO3_ATWRKY63_WRKY63__ABA overly sensitive mutant 3
2.56	10.62	1.36	9.95	0.00426996	AT2G13650	GONST1__golgi nucleotide sugar transporter 1
2.56	6.73	1.36	6.05	0.01590268	AT2G17850	Rhodanese/Cell cycle control phosphatase superfamily protein
2.56	13.47	1.35	12.79	0.01357873	AT2G23170	GH3.3__Auxin-responsive GH3 family protein
2.56	9.09	1.36	8.41	0.01600194	AT2G44581	RING/U-box superfamily protein
2.56	11.48	1.36	10.8	0.00382427	AT5G19100	Eukaryotic aspartyl protease family protein
2.55	12.49	1.35	11.82	0.01241845	AT5G49850	Mannose-binding lectin superfamily protein
2.55	12.14	1.35	11.46	0.00784613	ATCG01030	
2.54	13.33	1.35	12.65	0.00426996	AT1G17147	VQ motif-containing protein
2.54	12.73	1.35	12.06	0.00507483	AT1G17420	ATLOX3_LOX3__lipoxygenase 3
2.54	11.85	1.35	11.18	0.01150797	AT2G22860	ATPSK2_PSK2__phytosulfokine 2 precursor
2.54	12.37	1.35	11.7	0.00309366	AT2G32020	Acyl-CoA N-acyltransferases (NAT) superfamily protein
2.54	13.78	1.34	13.11	0.02046243	AT3G23550	MATE efflux family protein
2.54	12.7	1.35	12.03	0.00845049	AT3G48450	RPM1-interacting protein 4 (RIN4) family protein
2.54	11.81	1.35	11.14	0.00309366	AT5G32511	
2.53	11.92	1.34	11.25	0.00315341	AT1G61820	BGLU46__beta glucosidase 46 BGLU46__beta glucosidase 46
2.53	10.62	1.34	9.95	0.019243	AT4G19980	unknown protein; FUNCTIONS IN: molecular_function unknown; INVOLVED IN: biological_process unknown; LOCATED IN: chloroplast; EXPRESSED IN: root, pedicel; EXPRESSED DURING: 4 anthesis; Has 30201 Blast hits to 17322 proteins in 780 species: Archae - 12; Bacteria - 1396; Metazoa - 17338; Fungi - 3422; Plants - 5037; Viruses - 0; Other Eukaryotes - 2996 (source: NCBI BLink).
2.53	12.93	1.34	12.26	0.01231488	AT4G33070	Thiamine pyrophosphate dependent pyruvate decarboxylase family protein
2.53	13.68	1.34	13.01	0.00410995	AT5G25930	Protein kinase family protein with leucine-rich repeat domain
2.53	12.16	1.34	11.49	0.00873067	AT5G38200	Class I glutamine amidotransferase-like superfamily protein
2.53	9.83	1.34	9.16	0.01871038	AT5G57510	unknown protein; Has 27 Blast hits to 27 proteins in 9 species: Archae - 0; Bacteria - 0; Metazoa - 0; Fungi - 0; Plants - 27; Viruses - 0; Other Eukaryotes - 0 (source: NCBI BLink).
2.52	12.94	1.33	12.28	0.01371693	AT1G76680	ATOPR1_OPR1__12-oxophytodienoate reductase 1

2.52	9.77	1.33	9.1	0.00294186	AT5G31891	
2.51	10.47	1.33	9.8	0.02467167	AT2G16230	O-Glycosyl hydrolases family 17 protein
2.5	11.12	1.32	10.46	0.00420969	AT5G36925	unknown protein; BEST Arabidopsis thaliana protein match is: unknown protein (TAIR:AT5G36920.1); Has 9 Blast hits to 9 proteins in 2 species: Archae - 0; Bacteria - 0; Metazoa - 0; Fungi - 0; Plants - 9; Viruses - 0; Other Eukaryotes - 0 (source: NCBI BLink).
2.49	13.5	1.32	12.84	0.00603002	AT1G72416	Chaperone DnaJ-domain superfamily protein Chaperone DnaJ-domain superfamily protein Chaperone DnaJ-domain superfamily protein Chaperone DnaJ-domain superfamily protein
2.48	12.35	1.31	11.7	0.03671352	AT1G49230	RING/U-box superfamily protein
2.48	12.67	1.31	12.02	0.03724128	AT3G43190	ATSUS4_SUS4__sucrose synthase 4
2.47	12.52	1.31	11.86	0.0107702	AT1G19020	unknown protein; BEST Arabidopsis thaliana protein match is: unknown protein (TAIR:AT3G48180.1); Has 88 Blast hits to 88 proteins in 15 species: Archae - 0; Bacteria - 0; Metazoa - 0; Fungi - 0; Plants - 88; Viruses - 0; Other Eukaryotes - 0 (source: NCBI BLink).
2.47	12.3	1.3	11.64	0.02001256	AT2G39330	JAL23__jacalin-related lectin 23
2.46	14.53	1.3	13.88	0.00494369	AT1G25400	unknown protein; FUNCTIONS IN: molecular_function unknown; INVOLVED IN: biological_process unknown; LOCATED IN: cellular_component unknown; EXPRESSED IN: 20 plant structures; EXPRESSED DURING: 10 growth stages; BEST Arabidopsis thaliana protein match is: unknown protein (TAIR:AT1G68440.1).
2.46	12.25	1.3	11.6	0.00811381	AT3G15240	Serine/threonine-protein kinase WNK (With No Lysine)-related
2.46	12.71	1.3	12.06	0.01863454	AT5G13080	ATWRKY75_WRKY75__WRKY DNA-binding protein 75
2.45	6.82	1.29	6.17	0.01273993	AT1G30135	JAZ8_TIFY5A__jasmonate-zim-domain protein 8
2.45	12.38	1.29	11.73	0.02622766	AT1G52030	F-ATMBP_MBP1.2_MBP2__myrosinase-binding protein 2
2.45	13.61	1.29	12.96	0.00601931	AT1G72416	Chaperone DnaJ-domain superfamily protein Chaperone DnaJ-domain superfamily protein Chaperone DnaJ-domain superfamily protein Chaperone DnaJ-domain superfamily protein
2.45	12.51	1.29	11.87	0.02551503	AT4G06746	DEAR5_RAP2.9__related to AP2 9
2.44	13.35	1.29	12.71	0.00506342	AT1G28190	unknown protein; BEST Arabidopsis thaliana protein match is: unknown protein (TAIR:AT5G12340.1); Has 166 Blast hits to 162 proteins in 36 species: Archae - 0; Bacteria - 2; Metazoa - 15; Fungi - 5; Plants - 124; Viruses - 0; Other Eukaryotes - 20 (source: NCBI BLink).
2.44	13.86	1.28	13.22	0.00315341	AT3G61850	DAG1__Dof-type zinc finger DNA-binding family protein
2.44	10.26	1.29	9.62	0.00356968	AT4G06714	
2.44	11.24	1.29	10.59	0.00753143	AT4G30090	
2.44	10.87	1.29	10.22	0.0031719	AT5G29465	
2.43	12.2	1.28	11.55	0.003464	AT1G72060	serine-type endopeptidase inhibitors
2.42	11.29	1.28	10.65	0.01133233	AT1G30550	S-adenosyl-L-methionine-dependent methyltransferases superfamily protein
2.42	12.58	1.27	11.94	0.00568994	AT5G47960	ATRABA4C_RABA4C_SMG1__RAB GTPase homolog A4C
2.41	11.79	1.27	11.15	0.01324128	AT1G33750	Terpenoid cyclases/Protein prenyltransferases superfamily protein
2.41	13.37	1.27	12.74	0.02350016	AT1G49860	ATGSTF14_GSTF14__glutathione S-transferase (class phi) 14

2.41	7.25	1.27	6.62	0.04331841	AT2G03660	
2.41	10.78	1.27	10.14	0.00433482	AT5G32125	
2.4	12.34	1.26	11.71	0.01832168	AT1G10550	XET_XTH33__xyloglucan:xyloglucosyl transferase 33
2.4	12.19	1.26	11.56	0.00907007	AT3G22740	HMT3__homocysteine S-methyltransferase 3
2.4	11.85	1.27	11.21	0.00764764	AT4G23470	PLAC8 family protein
2.4	9.67	1.26	9.04	0.00746565	AT4G37290	unknown protein; FUNCTIONS IN: molecular_function unknown; INVOLVED IN: response to karrikin; LOCATED IN: endomembrane system; EXPRESSED IN: cotyledon, hypocotyl, leaf; EXPRESSED DURING: LP.04 four leaves visible; BEST Arabidopsis thaliana protein match is: unknown protein (TAIR:AT2G23270.1); Has 30201 Blast hits to 17322 proteins in 780 species: Archae - 12; Bacteria - 1396; Metazoa - 17338; Fungi - 3422; Plants - 5037; Viruses - 0; Other Eukaryotes - 2996 (source: NCBI BLink).
2.4	13.21	1.26	12.58	0.01166694	AT5G19520	ATMSL9_MSL9__mechanosensitive channel of small conductance-like 9
2.39	9.44	1.26	8.81	0.00528978	AT1G12610	DDF1__Integrase-type DNA-binding superfamily protein
2.39	12.37	1.26	11.74	0.00585398	AT1G67110	CYP735A2__cytochrome P450, family 735, subfamily A, polypeptide 2
2.39	13.03	1.25	12.4	0.00266801	AT5G59820	RHL41_ZAT12__C2H2-type zinc finger family protein
2.39	12.79	1.25	12.16	0.00420934	AT5G64660	ATCMPG2_CMPG2__CYS, MET, PRO, and GLY protein 2
2.38	9.64	1.25	9.02	0.00426996	AT1G38410	
2.38	14.35	1.25	13.72	0.00442816	ATCG00410	
2.37	11.94	1.24	11.32	0.00479295	AT2G06250	
2.37	10.59	1.25	9.97	0.00485976	AT3G19200	unknown protein; BEST Arabidopsis thaliana protein match is: unknown protein (TAIR:AT4G34419.1); Has 51 Blast hits to 51 proteins in 10 species: Archae - 0; Bacteria - 0; Metazoa - 0; Fungi - 0; Plants - 51; Viruses - 0; Other Eukaryotes - 0 (source: NCBI BLink).
2.37	9.76	1.25	9.13	0.01176783	AT3G24310	ATMYB71_MYB305__myb domain protein 305
2.37	9.92	1.24	9.3	0.00309366	AT5G33389	
2.37	9.07	1.24	8.45	0.01404349	AT5G41550	Disease resistance protein (TIR-NBS-LRR class) family
2.36	13.41	1.24	12.79	0.00833976	AT1G67810	SUFE2__sulfur E2
2.36	9.73	1.24	9.11	0.00897246	AT5G09980	PROPEP4__elicitor peptide 4 precursor
2.35	13.89	1.23	13.28	0.00294186	AT1G72360	AtERF73_ERF73_HRE1__Integrase-type DNA-binding superfamily protein AtERF73_ERF73_HRE1__Integrase-type DNA-binding superfamily protein
2.35	12.31	1.23	11.69	0.00634237	AT3G28340	GATL10_GolS8__galacturonosyltransferase-like 10
2.35	9.36	1.23	8.74	0.00463762	AT3G43157	
2.35	11.7	1.23	11.09	0.00429467	AT3G61190	BAP1__BON association protein 1
2.35	11.5	1.24	10.88	0.00660299	AT4G38340	Plant regulator RWP-RK family protein
2.34	13.68	1.23	13.07	0.04348436	AT2G41100	ATCAL4_TCH3__Calcium-binding EF hand family protein
2.34	11.51	1.23	10.9	0.00375986	AT3G30695	
2.34	11.81	1.23	11.2	0.00398842	AT3G33067	

2.34	12.51	1.23	11.9	0.00413198	AT3G51910	AT-HSFA7A_HSFA7A__heat shock transcription factor A7A
2.34	13.49	1.23	12.88	0.00495965	AT4G37390	AUR3_BRU6_GH3-2_GH3.2_YDK1__Auxin-responsive GH3 family protein
2.33	12.41	1.22	11.8	0.00686073	AT5G12340	unknown protein; BEST Arabidopsis thaliana protein match is: unknown protein (TAIR:AT1G28190.1); Has 30201 Blast hits to 17322 proteins in 780 species: Archae - 12; Bacteria - 1396; Metazoa - 17338; Fungi - 3422; Plants - 5037; Viruses - 0; Other Eukaryotes - 2996 (source: NCBI BLink).
2.33	9.15	1.22	8.54	0.00356968	AT5G44350	ethylene-responsive nuclear protein -related
2.33	13.08	1.22	12.47	0.03348692	AT5G65690	PCK2_PEPCK__phosphoenolpyruvate carboxykinase 2
2.32	14.4	1.22	13.79	0.00229082	AT1G24280	G6PD3__glucose-6-phosphate dehydrogenase 3
2.32	10.73	1.22	10.12	0.00309366	AT1G40072	
2.32	13.81	1.21	13.2	0.00517823	AT1G72360	AtERF73_ERF73_HRE1__Integrase-type DNA-binding superfamily protein AtERF73_ERF73_HRE1__Integrase-type DNA-binding superfamily protein
2.32	10.6	1.21	9.99	0.0061602	AT2G07789	
2.32	11.61	1.22	11	0.0046141	AT3G32880	
2.32	13.49	1.21	12.88	0.003464	AT3G47980	AtNITR2;2Integral membrane HPP family protein
2.32	14.64	1.21	14.04	0.00696469	AT4G11280	ACS6_ATACS6__1-aminocyclopropane-1-carboxylic acid (acc) synthase 6
2.32	12.88	1.21	12.27	0.00429467	AT4G24015	RING/U-box superfamily protein
2.32	7.92	1.21	7.31	0.00508871	AT5G33384	
2.31	10.83	1.21	10.22	0.00356968	AT1G40095	
2.31	10.46	1.21	9.85	0.00605413	AT1G67150	Plant protein of unknown function (DUF247) Plant protein of unknown function (DUF247) Plant protein of unknown function (DUF247)
2.31	11.12	1.21	10.52	0.00804254	AT2G36120	DOT1__Glycine-rich protein family
2.31	11.99	1.21	11.39	0.00507483	AT3G55090	ABCG16__ABC-2 type transporter family protein
2.31	12.3	1.21	11.69	0.01013972	AT5G52050	MATE efflux family protein
2.3	13.45	1.2	12.85	0.0047211	AT1G09460	Carbohydrate-binding X8 domain superfamily protein
2.3	10.28	1.2	9.68	0.00394474	AT1G43750	
2.3	11.15	1.2	10.55	0.00404201	AT4G06720	
2.29	14.27	1.2	13.67	0.00326047	AT1G32920	unknown protein; FUNCTIONS IN: molecular_function unknown; INVOLVED IN: response to wounding; LOCATED IN: endomembrane system; EXPRESSED IN: 23 plant structures; EXPRESSED DURING: 13 growth stages; BEST Arabidopsis thaliana protein match is: unknown protein (TAIR:AT1G32928.1); Has 42 Blast hits to 42 proteins in 8 species: Archae - 0; Bacteria - 0; Metazoa - 0; Fungi - 0; Plants - 42; Viruses - 0; Other Eukaryotes - 0 (source: NCBI BLink).
2.29	11.32	1.19	10.72	0.00356968	AT1G40093	
2.29	10.23	1.2	9.63	0.00453585	AT4G06666	
2.29	11.64	1.2	11.05	0.00394838	AT4G06712	
2.29	12.22	1.19	11.62	0.00309366	AT5G13700	APAO_ATPAO1_PAO1__polyamine oxidase 1

2.28	12.45	1.19	11.86	0.00801286	AT1G30040	ATGA2OX2_GA2OX2_GA2OX2__gibberellin 2-oxidase
2.28	10.99	1.19	10.4	0.00309366	AT1G40150	
2.28	10.5	1.19	9.91	0.00221781	AT2G34000	RING/U-box superfamily protein
2.28	13.08	1.19	12.49	0.00470122	AT3G02800	AtPFA-DSP3_PFA-DSP3__Tyrosine phosphatase family protein
2.28	8.02	1.19	7.43	0.00373629	AT4G04170	
2.28	11.6	1.19	11.01	0.00341128	AT4G06573	
2.28	8.93	1.19	8.34	0.00962979	AT5G29295	
2.28	12.62	1.19	12.02	0.00568994	AT5G57090	AGR_AGR1_ATPIN2_EIR1_MM31_PIN2_WAV6__Auxin efflux carrier family protein
2.28	10.07	1.19	9.47	0.04272545	AT5G66640	DAR3__DA1-related protein 3
2.27	13.26	1.18	12.66	0.00417125	AT1G14540	Peroxidase superfamily protein
2.27	13.05	1.18	12.46	0.0134514	AT1G32928	unknown protein; BEST Arabidopsis thaliana protein match is: unknown protein (TAIR:AT1G32920.1); Has 35333 Blast hits to 34131 proteins in 2444 species: Archae - 798; Bacteria - 22429; Metazoa - 974; Fungi - 991; Plants - 531; Viruses - 0; Other Eukaryotes - 9610 (source: NCBI BLink).
2.27	11.41	1.18	10.82	0.00811178	AT1G52560	HSP20-like chaperones superfamily protein HSP20-like chaperones superfamily protein
2.27	13.49	1.18	12.9	0.00309366	AT1G61820	BGLU46__beta glucosidase 46 BGLU46__beta glucosidase 46
2.27	10.1	1.18	9.51	0.01079271	AT2G04500	Cysteine/Histidine-rich C1 domain family protein
2.27	12.78	1.18	12.19	0.01420608	AT2G07743	
2.27	9.28	1.18	8.69	0.00928874	AT2G18200	unknown protein; FUNCTIONS IN: molecular_function unknown; INVOLVED IN: biological_process unknown; LOCATED IN: mitochondrion; BEST Arabidopsis thaliana protein match is: unknown protein (TAIR:AT2G18210.1); Has 19 Blast hits to 19 proteins in 2 species: Archae - 0; Bacteria - 0; Metazoa - 0; Fungi - 0; Plants - 19; Viruses - 0; Other Eukaryotes - 0 (source: NCBI BLink).
2.27	11.14	1.18	10.55	0.01615124	AT4G32800	Integrase-type DNA-binding superfamily protein
2.27	10.55	1.18	9.95	0.00601931	AT5G29550	
2.27	11.73	1.18	11.14	0.00426996	AT5G32107	
2.27	13.47	1.18	12.88	0.01998812	AT5G63660	LCR74_PDF2.5__Scorpion toxin-like knottin superfamily protein
2.27	12.23	1.18	11.64	0.02355063	AT5G66940	Dof-type zinc finger DNA-binding family protein
2.26	12.56	1.18	11.97	0.00939186	AT1G02810	Plant invertase/pectin methylesterase inhibitor superfamily
2.26	9.83	1.17	9.24	0.01156554	AT1G39270	
2.26	11.44	1.18	10.86	0.02108292	AT2G18480	Major facilitator superfamily protein
2.26	11.46	1.17	10.87	0.00714664	AT3G43303	
2.26	12.7	1.18	12.12	0.00508871	AT4G00940	Dof-type zinc finger DNA-binding family protein
2.26	12.45	1.18	11.86	0.01398274	AT5G48940	Leucine-rich repeat transmembrane protein kinase family protein
2.26	10.8	1.18	10.21	0.02583341	AT5G62330	BEST Arabidopsis thaliana protein match is: Plant invertase/pectin methylesterase inhibitor superfamily protein (TAIR:AT5G62340.1); Has 9 Blast hits to 9 proteins in 2 species: Archae - 0;

						Bacteria - 0; Metazoa - 0; Fungi - 0; Plants - 9; Viruses - 0; Other Eukaryotes - 0 (source: NCBI BLink).
2.25	10.36	1.17	9.78	0.00756439	AT1G69920	ATGSTU12_GSTU12__glutathione S-transferase TAU 12
2.25	14.12	1.17	13.53	0.00294186	AT1G71400	AtRLP12_RLP12__receptor like protein 12
2.25	13.23	1.17	12.65	0.00438308	AT2G07825	unknown protein; Has 30201 Blast hits to 17322 proteins in 780 species: Archae - 12; Bacteria - 1396; Metazoa - 17338; Fungi - 3422; Plants - 5037; Viruses - 0; Other Eukaryotes - 2996 (source: NCBI BLink).
2.24	12.74	1.16	12.16	0.00610609	AT1G33790	jacalin lectin family protein jacalin lectin family protein
2.24	10.84	1.16	10.26	0.00694229	AT1G67150	Plant protein of unknown function (DUF247) Plant protein of unknown function (DUF247) Plant protein of unknown function (DUF247)
2.24	10.65	1.17	10.07	0.01391174	AT2G23630	sks16__SKU5 similar 16
2.24	8.47	1.16	7.89	0.0097881	AT2G31920	Plant protein of unknown function (DUF936)
2.24	10.57	1.16	9.99	0.02443705	AT3G30340	nodulin MtN21 /EamA-like transporter family protein
2.24	8.97	1.16	8.39	0.00323071	AT4G08104	
2.24	9.03	1.16	8.45	0.00593185	AT4G08114	
2.24	12.38	1.17	11.79	0.02573479	AT5G13900	Bifunctional inhibitor/lipid-transfer protein/seed storage 2S albumin superfamily protein
2.24	7.68	1.16	7.1	0.01448673	AT5G29436	
2.24	9.81	1.17	9.22	0.00514085	AT5G31719	
2.24	11.18	1.16	10.6	0.00525197	AT5G31855	
2.24	13.18	1.16	12.6	0.01645617	ATCG00230	
2.23	9.76	1.16	9.18	0.00479295	AT1G37537	
2.23	10.77	1.16	10.19	0.00540858	AT1G39990	
2.23	10.68	1.16	10.1	0.00750343	AT1G67150	Plant protein of unknown function (DUF247) Plant protein of unknown function (DUF247) Plant protein of unknown function (DUF247)
2.23	9.5	1.16	8.92	0.02742724	AT3G30350	RGF4__Encodes a root meristem growth factor (RGF). Belongs to a family of functionally redundant homologous peptides that are secreted, tyrosine-sulfated, and expressed mainly in the stem cell area and the innermost layer of central columella cells. RGFs are required for maintenance of the root stem cell niche and transit amplifying cell proliferation. Members of this family include: At5g60810 (RGF1), At1g13620 (RGF2), At2g04025 (RGF3), At3g30350 (RGF4), At5g51451 (RGF5), At4g16515 (RGF6), At3g02240 (RGF7), At2g03830 (RGF8) and At5g64770 (RGF9).
2.23	10.39	1.16	9.81	0.00306866	AT3G30767	
2.23	9.29	1.16	8.71	0.00358222	AT4G06578	
2.23	12.42	1.15	11.84	0.0047933	AT4G06656	
2.23	10.01	1.16	9.43	0.0032853	AT4G07456	
2.23	12.43	1.16	11.85	0.00608255	AT4G15120	VQ motif-containing protein

2.23	12.92	1.16	12.34	0.00760062	AT5G08350	GRAM domain-containing protein / ABA-responsive protein-related
2.23	12.45	1.16	11.87	0.00902402	AT5G35580	Protein kinase superfamily protein
2.22	10.96	1.15	10.38	0.01048515	AT1G52560	HSP20-like chaperones superfamily protein HSP20-like chaperones superfamily protein
2.22	8.43	1.15	7.85	0.00422741	AT2G09865	
2.22	11.59	1.15	11.01	0.00429467	AT2G09930	
2.22	10.67	1.15	10.09	0.00507084	AT2G10880	
2.22	11.23	1.15	10.66	0.02026545	AT2G23050	MEL4_NPY4__Phototropic-responsive NPH3 family protein
2.22	9.4	1.15	8.82	0.00696556	AT2G45930	unknown protein; FUNCTIONS IN: molecular_function unknown; INVOLVED IN: biological_process unknown; LOCATED IN: mitochondrion; BEST Arabidopsis thaliana protein match is: unknown protein (TAIR:AT4G16090.1); Has 161 Blast hits to 161 proteins in 2 species: Archae - 0; Bacteria - 0; Metazoa - 0; Fungi - 0; Plants - 161; Viruses - 0; Other Eukaryotes - 0 (source: NCBI BLink).
2.22	11.27	1.15	10.69	0.00309366	AT3G42253	
2.22	15.04	1.15	14.47	0.00294186	AT4G24570	DIC2__dicarboxylate carrier 2
2.22	8.67	1.15	8.09	0.00356968	AT5G17960	Cysteine/Histidine-rich C1 domain family protein
2.22	10.82	1.15	10.24	0.00876594	AT5G31087	
2.22	10.02	1.15	9.45	0.00592108	AT5G31980	
2.22	9.44	1.15	8.86	0.02146312	AT5G31989	
2.22	7.47	1.15	6.89	0.01804554	AT5G32580	
2.21	13.42	1.14	12.85	0.02114033	AT1G33055	unknown protein; FUNCTIONS IN: molecular_function unknown; INVOLVED IN: anaerobic respiration; LOCATED IN: endomembrane system; EXPRESSED IN: 13 plant structures; EXPRESSED DURING: 6 growth stages; Has 20 Blast hits to 20 proteins in 8 species: Archae - 0; Bacteria - 0; Metazoa - 0; Fungi - 0; Plants - 20; Viruses - 0; Other Eukaryotes - 0 (source: NCBI BLink).
2.21	9.99	1.14	9.42	0.00294186	AT1G40101	
2.21	11.78	1.15	11.21	0.004055	AT2G12832	
2.21	12.72	1.15	12.14	0.00356968	AT3G15990	SULTR3;4__sulfate transporter 3;4
2.21	10.37	1.15	9.8	0.00410995	AT3G32000	
2.21	10.89	1.15	10.31	0.00744705	AT3G33066	
2.21	10.9	1.15	10.33	0.00294186	AT4G06517	
2.21	11.75	1.14	11.18	0.00382427	AT4G14370	Disease resistance protein (TIR-NBS-LRR class) family
2.21	8.23	1.14	7.66	0.00730819	AT5G51480	SKS2__SKU5 similar 2
2.2	12.06	1.14	11.49	0.00470122	AT1G19540	NmrA-like negative transcriptional regulator family protein
2.2	10.6	1.14	10.03	0.00411612	AT3G29730	
2.2	10.22	1.14	9.66	0.00286412	AT3G33127	
2.2	11.68	1.14	11.11	0.00449194	AT5G32179	

2.2	14.36	1.14	13.79	0.003464	AT5G61600	ERF104__ethylene response factor 104
2.19	6.99	1.13	6.42	0.00488479	AT1G35610	Cysteine/Histidine-rich C1 domain family protein
2.19	7.94	1.13	7.37	0.00432857	AT2G13270	
2.19	10.8	1.13	10.24	0.01962777	AT2G45760	BAL_BAP2__BON association protein 2
2.19	9.9	1.13	9.34	0.00395842	AT3G33077	
2.19	12.39	1.13	11.83	0.0112008	AT4G37160	sks15__SKU5 similar 15
2.18	9.27	1.12	8.71	0.00442816	AT1G40074	
2.18	13.29	1.12	12.73	0.00429467	AT3G20362	unknown protein; FUNCTIONS IN: molecular_function unknown; INVOLVED IN: biological_process unknown; LOCATED IN: endomembrane system; Has 4 Blast hits to 4 proteins in 1 species: Archae - 0; Bacteria - 0; Metazoa - 0; Fungi - 0; Plants - 4; Viruses - 0; Other Eukaryotes - 0 (source: NCBI BLink).
2.18	12.99	1.12	12.43	0.00455724	AT3G26770	NAD(P)-binding Rossmann-fold superfamily protein
2.18	11.01	1.13	10.44	0.00508871	AT4G06735	
2.18	10.68	1.12	10.12	0.00761033	AT4G07355	
2.18	11.5	1.13	10.94	0.00426996	AT5G34970	
2.18	12.81	1.13	12.25	0.01281651	ATMG01070	
2.17	11.65	1.12	11.09	0.02424045	AT1G12950	RSH2__root hair specific 2
2.17	9.42	1.12	8.86	0.02042632	AT1G19180	JAZ1_TIFY10A__jasmonate-zim-domain protein 1
2.17	13.96	1.12	13.4	0.03453974	AT1G27020	unknown protein; BEST Arabidopsis thaliana protein match is: unknown protein (TAIR:AT1G27030.1); Has 514 Blast hits to 514 proteins in 175 species: Archae - 0; Bacteria - 311; Metazoa - 0; Fungi - 0; Plants - 65; Viruses - 0; Other Eukaryotes - 138 (source: NCBI BLink).
2.17	13.23	1.12	12.67	0.01773007	AT2G35770	scpl28__serine carboxypeptidase-like 28
2.17	12.07	1.12	11.51	0.00540585	AT4G06485	
2.17	8.9	1.12	8.34	0.00543099	AT4G07688	
2.17	11.09	1.12	10.53	0.0039427	AT5G32053	
2.16	11.87	1.11	11.31	0.00603875	AT1G33790	jacalin lectin family protein jacalin lectin family protein
2.16	12.96	1.11	12.4	0.00284669	AT2G39650	Protein of unknown function (DUF506)
2.16	11.86	1.11	11.31	0.00309366	AT4G06730	
2.16	10.53	1.11	9.97	0.00885817	AT5G31572	
2.16	10.62	1.11	10.06	0.00514085	AT5G32516	
2.16	7.51	1.11	6.96	0.00481576	AT5G40370	GRXC2__Glutaredoxin family protein
2.16	13.03	1.11	12.47	0.00480388	AT5G51780	basic helix-loop-helix (bHLH) DNA-binding superfamily protein
2.16	14.42	1.11	13.87	0.00371237	ATCG00460	
2.15	10.44	1.1	9.89	0.02340024	AT1G13480	Protein of unknown function (DUF1262)
2.15	13.68	1.11	13.13	0.03639728	AT1G15125	S-adenosyl-L-methionine-dependent methyltransferases superfamily protein

2.15	11.45	1.1	10.9	0.00751295	AT1G22470	unknown protein; BEST Arabidopsis thaliana protein match is: unknown protein (TAIR:AT1G72240.1); Has 1693 Blast hits to 236 proteins in 54 species: Archae - 0; Bacteria - 8; Metazoa - 451; Fungi - 116; Plants - 94; Viruses - 2; Other Eukaryotes - 1022 (source: NCBI BLink).
2.15	8.69	1.1	8.14	0.00294186	AT1G38280	
2.15	10.65	1.1	10.1	0.00286412	AT1G40100	
2.15	11.69	1.11	11.14	0.0067964	AT2G01818	PLATZ transcription factor family protein
2.15	11.72	1.1	11.17	0.00413041	AT2G10280	
2.15	12.35	1.1	11.8	0.03245312	AT3G12145	FLOR1_FLR1__Leucine-rich repeat (LRR) family protein
2.15	9.14	1.1	8.59	0.01226707	AT3G19615	unknown protein; FUNCTIONS IN: molecular_function unknown; INVOLVED IN: biological_process unknown; LOCATED IN: cellular_component unknown; Has 10 Blast hits to 10 proteins in 5 species: Archae - 0; Bacteria - 0; Metazoa - 0; Fungi - 0; Plants - 10; Viruses - 0; Other Eukaryotes - 0 (source: NCBI BLink).
2.15	10.92	1.11	10.36	0.00407275	AT3G37820	
2.15	11.25	1.1	10.7	0.00540858	AT4G06606	
2.15	8.82	1.11	8.26	0.00625636	AT4G36820	Protein of unknown function (DUF607)
2.15	11.37	1.11	10.82	0.00581265	AT5G29975	
2.15	11.04	1.11	10.49	0.00434991	AT5G31736	
2.15	11.01	1.1	10.45	0.00426996	AT5G32071	
2.15	9.51	1.1	8.96	0.00603002	AT5G35010	
2.14	10.38	1.1	9.83	0.0070466	AT1G37867	
2.14	10.87	1.09	10.32	0.00468774	AT2G10670	
2.14	10.03	1.1	9.48	0.01269878	AT2G14820	MEL3_NPY2__Phototropic-responsive NPH3 family protein
2.14	10.26	1.09	9.71	0.01226263	AT2G29710	UDP-Glycosyltransferase superfamily protein
2.14	9.67	1.1	9.12	0.00489929	AT2G45080	cycp3;1__cyclin p3;1
2.14	13.1	1.1	12.55	0.00356968	AT3G04640	glycine-rich protein
2.14	12.08	1.1	11.54	0.00542693	AT3G42252	
2.14	11.93	1.1	11.38	0.00356968	AT3G47210	Plant protein of unknown function (DUF247)
2.14	14.26	1.1	13.71	0.01714177	AT3G49110	ATPCA_ATPRX33_PRX33_PRXCA__peroxidase CA
2.14	13.89	1.1	13.34	0.00434623	AT4G05390	ATRFNR1_RFNR1__root FNR 1
2.14	9.99	1.1	9.44	0.00294186	AT4G06625	
2.14	8.67	1.1	8.12	0.00318627	AT4G07620	
2.14	10.73	1.1	10.19	0.00470122	AT4G37295	unknown protein; FUNCTIONS IN: molecular_function unknown; INVOLVED IN: biological_process unknown; LOCATED IN: endomembrane system; EXPRESSED IN: stem, root, leaf; EXPRESSED DURING: LP.06 six leaves visible, LP.04 four leaves visible; Has 30201 Blast hits to 17322 proteins in 780 species: Archae - 12; Bacteria - 1396; Metazoa - 17338; Fungi -

						3422; Plants - 5037; Viruses - 0; Other Eukaryotes - 2996 (source: NCBI BLink).
2.14	14.04	1.1	13.49	0.0047211	AT5G10830	S-adenosyl-L-methionine-dependent methyltransferases superfamily protein
2.14	10.24	1.1	9.69	0.00356968	AT5G30673	
2.14	12.61	1.1	12.06	0.00438643	AT5G31787	
2.14	11.66	1.1	11.12	0.00528978	AT5G32405	
2.14	10.66	1.1	10.11	0.005834	AT5G35142	
2.14	8.67	1.1	8.12	0.02979537	AT5G57980	RPB5C__RNA polymerase II fifth largest subunit, C
2.14	14.3	1.09	13.75	0.00266801	ATCG00940	
2.13	10.17	1.09	9.62	0.02464648	AT1G14550	Peroxidase superfamily protein
2.13	14.09	1.09	13.54	0.00394838	AT1G20510	OPCL1__OPC-8:0 CoA ligase1 OPCL1__OPC-8:0 CoA ligase1
2.13	9.32	1.09	8.77	0.01027468	AT1G41930	
2.13	11.61	1.09	11.06	0.0040014	AT3G32220	
2.13	11	1.09	10.45	0.00617912	AT3G42256	
2.13	10.69	1.09	10.15	0.00294186	AT3G43864	
2.13	11.53	1.09	10.99	0.00428228	AT4G07458	
2.13	9.43	1.09	8.88	0.00638451	AT4G14250	structural constituent of ribosome
2.13	11.61	1.09	11.07	0.00568994	AT4G31020	alpha/beta-Hydrolases superfamily protein
2.13	10.13	1.09	9.59	0.01315501	AT5G07040	RING/U-box superfamily protein
2.13	12.94	1.09	12.39	0.00662165	ATMG00450	ORF106B__hypothetical protein
2.12	10.76	1.08	10.22	0.00286412	AT1G32720	Cytochrome C oxidase polypeptide VIB family protein
2.12	10.42	1.09	9.87	0.00666122	AT1G39750	
2.12	10.05	1.08	9.51	0.03670394	AT1G48510	Surfeit locus 1 cytochrome c oxidase biogenesis protein
2.12	12.75	1.08	12.21	0.00614252	AT2G07749	Mitovirus RNA-dependent RNA polymerase
2.12	13.48	1.08	12.94	0.00356968	AT2G28780	unknown protein; INVOLVED IN: biological_process unknown; LOCATED IN: mitochondrion; EXPRESSED IN: inflorescence meristem, root, flower; EXPRESSED DURING: petal differentiation and expansion stage; CONTAINS InterPro DOMAIN/s: Protein of unknown function DUF939, bacterial (InterPro:IPR010343); BEST Arabidopsis thaliana protein match is: unknown protein (TAIR:AT3G09450.1); Has 671 Blast hits to 667 proteins in 305 species: Archae - 0; Bacteria - 588; Metazoa - 0; Fungi - 2; Plants - 66; Viruses - 0; Other Eukaryotes - 15 (source: NCBI BLink).
2.12	14.89	1.08	14.34	0.00374452	AT2G38470	ATWRKY33_WRKY33__WRKY DNA-binding protein 33
2.12	14.06	1.08	13.51	0.00296119	AT3G16720	ATL2_TL2__TOXICOS EN LEVADURA 2
2.12	12.33	1.08	11.79	0.01003984	AT4G01525	
2.12	12.66	1.09	12.11	0.00627621	AT5G10510	AIL6_PLT3__AINTEGUMENTA-like 6 AIL6_PLT3__AINTEGUMENTA-like 6
2.12	12.82	1.08	12.28	0.00546333	AT5G32312	
2.12	10.77	1.09	10.22	0.00617387	AT5G32750	

2.12	10.52	1.08	9.98	0.00669899	AT5G57500	Galactosyltransferase family protein
2.11	13.05	1.08	12.51	0.0099573	AT1G02400	ATGA2OX4_ATGA2OX6_DTA1_GA2OX6__gibberellin 2-oxidase 6
2.11	10.04	1.08	9.5	0.00518233	AT1G38350	
2.11	10.42	1.08	9.88	0.00452251	AT1G42370	
2.11	12.73	1.08	12.19	0.00576543	AT2G07830	unknown protein; LOCATED IN: endomembrane system; Has 30201 Blast hits to 17322 proteins in 780 species: Archae - 12; Bacteria - 1396; Metazoa - 17338; Fungi - 3422; Plants - 5037; Viruses - 0; Other Eukaryotes - 2996 (source: NCBI BLink).
2.11	12.45	1.08	11.91	0.00999489	AT3G04320	Kunitz family trypsin and protease inhibitor protein
2.11	13.98	1.08	13.44	0.00356968	AT3G18690	MKS1__MAP kinase substrate 1
2.11	9.56	1.08	9.02	0.00229082	AT3G33100	
2.11	11.27	1.08	10.73	0.00422741	AT4G07942	
2.11	11.35	1.07	10.81	0.00459015	AT5G08360	Protein of unknown function (DUF789)
2.11	12.67	1.08	12.13	0.0165731	AT5G11920	AtcwINV6_cwINV6__6-&1-fructan exohydrolase
2.11	10.99	1.07	10.45	0.00605413	AT5G32197	
2.11	10.54	1.08	10.01	0.00554683	AT5G32345	
2.11	8.62	1.08	8.08	0.00640968	AT5G33237	
2.11	9.37	1.08	8.83	0.00760062	AT5G33427	
2.11	9.71	1.08	9.17	0.04650419	AT5G44620	CYP706A3__cytochrome P450, family 706, subfamily A, polypeptide 3
2.11	14.98	1.08	14.44	0.00593185	ATCG00850	
2.11	12.62	1.08	12.08	0.00483365	ATMG00513	NAD5_NAD5.1_NAD5A__NADH dehydrogenase 5A
2.1	9.07	1.07	8.53	0.00784132	AT1G32970	Subtilisin-like serine endopeptidase family protein
2.1	10.22	1.07	9.69	0.0100502	AT1G38450	
2.1	10.84	1.07	10.3	0.00490771	AT1G42375	
2.1	13.94	1.07	13.4	0.02665442	AT1G53885	Protein of unknown function (DUF581)
2.1	10.41	1.07	9.88	0.00565467	AT2G14960	GH3.1__Auxin-responsive GH3 family protein
2.1	11.27	1.07	10.74	0.00309366	AT3G03280	unknown protein; FUNCTIONS IN: molecular_function unknown; INVOLVED IN: N-terminal protein myristoylation; LOCATED IN: cellular_component unknown; BEST Arabidopsis thaliana protein match is: unknown protein (TAIR:AT5G17350.1); Has 137 Blast hits to 137 proteins in 11 species: Archae - 0; Bacteria - 0; Metazoa - 0; Fungi - 0; Plants - 137; Viruses - 0; Other Eukaryotes - 0 (source: NCBI BLink).
2.1	14.13	1.07	13.59	0.00459015	AT3G15630	unknown protein; FUNCTIONS IN: molecular_function unknown; INVOLVED IN: biological_process unknown; LOCATED IN: chloroplast; EXPRESSED IN: 24 plant structures; EXPRESSED DURING: 15 growth stages; BEST Arabidopsis thaliana protein match is: unknown protein (TAIR:AT1G52720.1); Has 61 Blast hits to 61 proteins in 13 species: Archae - 0; Bacteria - 0; Metazoa - 0; Fungi - 0; Plants - 61; Viruses - 0; Other Eukaryotes - 0 (source: NCBI BLink).
2.1	10.19	1.07	9.65	0.00309366	AT3G30645	

2.1	11.3	1.07	10.77	0.0048453	AT3G30700	
2.1	10.28	1.07	9.74	0.00731852	AT3G42445	
2.1	11.43	1.07	10.9	0.00650093	AT3G43862	
2.1	11.75	1.07	11.21	0.00420934	AT4G07485	
2.1	12.97	1.07	12.44	0.00894842	AT4G19880	Glutathione S-transferase family protein
2.1	11.64	1.07	11.1	0.01467773	AT4G33985	Protein of unknown function (DUF1685)
2.1	14.67	1.07	14.13	0.00356968	AT4G36500	unknown protein; FUNCTIONS IN: molecular_function unknown; INVOLVED IN: biological_process unknown; LOCATED IN: mitochondrion; EXPRESSED IN: 22 plant structures; EXPRESSED DURING: 13 growth stages; BEST Arabidopsis thaliana protein match is: unknown protein (TAIR:AT2G18210.1); Has 50 Blast hits to 50 proteins in 7 species: Archae - 0; Bacteria - 0; Metazoa - 0; Fungi - 0; Plants - 50; Viruses - 0; Other Eukaryotes - 0 (source: NCBI BLink).
2.1	13.76	1.07	13.22	0.00886775	AT5G12940	Leucine-rich repeat (LRR) family protein
2.1	10.73	1.07	10.19	0.01033221	AT5G38700	unknown protein; FUNCTIONS IN: molecular_function unknown; INVOLVED IN: biological_process unknown; LOCATED IN: mitochondrion; EXPRESSED IN: 10 plant structures; EXPRESSED DURING: LP.04 four leaves visible, LP.10 ten leaves visible, petal differentiation and expansion stage, LP.08 eight leaves visible, LP.12 twelve leaves visible; BEST Arabidopsis thaliana protein match is: unknown protein (TAIR:AT4G02170.1); Has 64 Blast hits to 64 proteins in 13 species: Archae - 0; Bacteria - 0; Metazoa - 0; Fungi - 0; Plants - 64; Viruses - 0; Other Eukaryotes - 0 (source: NCBI BLink).
2.1	10.59	1.07	10.06	0.02917437	AT5G44417	
2.1	8.15	1.07	7.61	0.00394474	AT5G57126	
2.09	11.04	1.06	10.51	0.00946772	AT1G42365	
2.09	11.21	1.07	10.68	0.0052529	AT1G72210	basic helix-loop-helix (bHLH) DNA-binding superfamily protein
2.09	12.27	1.06	11.74	0.0385492	AT1G74500	ATBS1_BS1_TMO7__activation-tagged BRI1(brassinosteroid-insensitive 1)-suppressor 1
2.09	14.02	1.06	13.49	0.01024343	AT2G22500	ATPUMP5_DIC1_UCP5__uncoupling protein 5
2.09	14.34	1.06	13.81	0.00434623	AT2G26530	AR781__Protein of unknown function (DUF1645)
2.09	13.36	1.06	12.83	0.00611309	AT2G39350	ABCG1__ABC-2 type transporter family protein
2.09	9.55	1.07	9.02	0.01213203	AT3G42990	unknown protein; Has 1 Blast hits to 1 proteins in 1 species: Archae - 0; Bacteria - 0; Metazoa - 0; Fungi - 0; Plants - 1; Viruses - 0; Other Eukaryotes - 0 (source: NCBI BLink).
2.09	12.76	1.06	12.23	0.00904592	AT3G44326	F-box family protein
2.09	11.1	1.06	10.57	0.01388666	AT3G62210	EDA32__Putative endonuclease or glycosyl hydrolase
2.09	11.41	1.06	10.88	0.00563172	AT4G07941	
2.09	9.81	1.07	9.28	0.00855569	AT4G39320	microtubule-associated protein-related
2.09	8.36	1.06	7.83	0.00294186	AT5G28165	
2.09	9.3	1.06	8.77	0.00420934	AT5G31668	

2.09	12.89	1.06	12.36	0.00475865	AT5G32161	
2.08	10.85	1.06	10.32	0.01297327	AT1G20510	OPCL1__OPC-8:0 CoA ligase1 OPCL1__OPC-8:0 CoA ligase1
2.08	8.31	1.05	7.78	0.00512277	AT1G37405	
2.08	10.22	1.06	9.69	0.00479295	AT1G42360	
2.08	9.91	1.06	9.38	0.00514085	AT2G06150	
2.08	13.68	1.05	13.15	0.00581482	AT2G07733	
2.08	10.68	1.06	10.15	0.00459015	AT2G13010	
2.08	10.85	1.06	10.32	0.02184334	AT3G14540	Terpenoid cyclases/Protein prenyltransferases superfamily protein
2.08	8.42	1.06	7.89	0.01417286	AT3G31390	
2.08	9.46	1.05	8.93	0.00788305	AT4G03770	
2.08	10.78	1.06	10.25	0.01259768	AT4G05591	
2.08	8.45	1.06	7.92	0.01297327	AT4G06628	
2.08	11.73	1.06	11.21	0.00646462	AT4G38400	ATEXLA2_ATEXPL2_ATHEXP BETA 2.2_EXLA2_EXPL2__expansin-like A2
2.08	9.32	1.06	8.79	0.00356968	AT5G05365	Heavy metal transport/detoxification superfamily protein
2.08	11.5	1.06	10.97	0.0064896	AT5G32306	
2.08	11.39	1.06	10.86	0.00924191	AT5G32475	
2.08	11.85	1.06	11.33	0.00660299	AT5G32486	
2.08	10.77	1.06	10.24	0.00834013	AT5G33383	
2.08	11.38	1.06	10.86	0.00981356	AT5G53290	CRF3__cytokinin response factor 3
2.07	10.79	1.05	10.27	0.00518233	AT1G08090	ACH1_ATNRT2.1_ATNRT2:1_LIN1_NRT2_NRT2.1_NRT2:1_NRT2;1AT__nitrate transporter 2:1
2.07	11.13	1.05	10.61	0.00548055	AT1G36520	
2.07	9.55	1.05	9.03	0.00755929	AT1G39910	
2.07	9.14	1.05	8.62	0.00649867	AT2G06965	
2.07	11	1.05	10.48	0.0039413	AT2G09187	
2.07	8.95	1.05	8.43	0.00738846	AT2G13020	
2.07	10.53	1.05	10.01	0.01476349	AT2G32140	transmembrane receptors
2.07	9.81	1.05	9.29	0.0047211	AT3G42750	
2.07	12.98	1.05	12.45	0.02633943	AT3G60140	BGLU30_DIN2_SRG2__Glycosyl hydrolase superfamily protein
2.07	9.57	1.05	9.05	0.03677134	AT4G11480	CRK32__cysteine-rich RLK (RECEPTOR-like protein kinase) 32
2.07	11.56	1.05	11.04	0.03069242	AT4G22235	Arabidopsis defensin-like protein
2.07	7.69	1.05	7.16	0.00447862	AT4G23515	Toll-Interleukin-Resistance (TIR) domain family protein
2.07	13.83	1.05	13.31	0.00850967	AT4G30280	ATXTH18_XTH18__xyloglucan endotransglucosylase/hydrolase 18
2.07	13.44	1.05	12.92	0.01311534	AT5G18470	Curculin-like (mannose-binding) lectin family protein
2.07	13.53	1.05	13	0.01693252	AT5G19410	ABCG23__ABC-2 type transporter family protein
2.07	8.31	1.05	7.79	0.00229082	AT5G29053	

2.07	8.8	1.05	8.28	0.02163718	AT5G55650	unknown protein; Has 1807 Blast hits to 1807 proteins in 277 species: Archae - 0; Bacteria - 0; Metazoa - 736; Fungi - 347; Plants - 385; Viruses - 0; Other Eukaryotes - 339 (source: NCBI BLink).
2.07	14.99	1.05	14.46	0.00288203	AT5G59550	zinc finger (C3HC4-type RING finger) family protein
2.07	10.62	1.05	10.09	0.00610609	AT5G60310	Concanavalin A-like lectin protein kinase family protein
2.07	14.86	1.05	14.34	0.00528978	ATCG00330	RPS14__chloroplast ribosomal protein S14
2.06	13.7	1.05	13.18	0.00306866	AT1G06620	2-oxoglutarate (2OG) and Fe(II)-dependent oxygenase superfamily protein
2.06	10.31	1.05	9.79	0.00753143	AT1G36795	
2.06	13.66	1.04	13.14	0.00981356	AT1G80240	Protein of unknown function, DUF642
2.06	10.44	1.05	9.91	0.00489929	AT2G23270	unknown protein; FUNCTIONS IN: molecular_function unknown; INVOLVED IN: biological_process unknown; LOCATED IN: endomembrane system; EXPRESSED IN: stem, sperm cell, root, stamen; EXPRESSED DURING: 4 anthesis; BEST Arabidopsis thaliana protein match is: unknown protein (TAIR:AT4G37290.1); Has 36 Blast hits to 35 proteins in 6 species: Archae - 0; Bacteria - 0; Metazoa - 0; Fungi - 0; Plants - 36; Viruses - 0; Other Eukaryotes - 0 (source: NCBI BLink).
2.06	9.46	1.04	8.94	0.00294186	AT3G32116	
2.06	13.75	1.04	13.23	0.01246168	AT3G45970	ATEXLA1_ATEXPL1_ATHEXP BETA 2.1_EXLA1_EXPL1__expansin-like A1
2.06	13.4	1.04	12.88	0.00751067	AT3G51450	Calcium-dependent phosphotriesterase superfamily protein
2.06	10.92	1.04	10.4	0.00373973	AT4G06626	
2.06	11.36	1.04	10.83	0.00744327	AT4G06664	
2.06	11.88	1.04	11.35	0.00417546	AT4G06704	
2.06	11.83	1.04	11.31	0.01349985	AT5G14020	Endosomal targeting BRO1-like domain-containing protein
2.06	11.57	1.04	11.05	0.00473458	AT5G33150	
2.06	9.88	1.04	9.36	0.00787903	AT5G36070	
2.06	12.3	1.04	11.78	0.02021372	AT5G47530	Auxin-responsive family protein
2.06	13.73	1.04	13.21	0.00592108	AT5G48070	ATXTH20_XTH20__xyloglucan endotransglucosylase/hydrolase 20
2.06	12.55	1.05	12.03	0.02183083	AT5G52320	CYP96A4__cytochrome P450, family 96, subfamily A, polypeptide 4
2.06	14.93	1.04	14.41	0.00487538	ATCG00890	NDHB.1__NADH-Ubiquinone/plastoquinone (complex I) protein
2.05	10.63	1.03	10.11	0.00630748	AT1G22280	PAPP2C__phytochrome-associated protein phosphatase type 2C
2.05	10.01	1.03	9.49	0.00480388	AT1G36460	
2.05	10.89	1.03	10.37	0.00453585	AT1G40117	
2.05	9.47	1.04	8.95	0.00315341	AT1G41630	
2.05	11.37	1.04	10.86	0.00794236	AT1G42050	
2.05	12.35	1.03	11.83	0.00788652	AT1G59860	HSP20-like chaperones superfamily protein
2.05	10.48	1.04	9.96	0.00540858	AT2G09950	
2.05	10.93	1.04	10.42	0.01254852	AT2G10140	

2.05	10.16	1.04	9.64	0.00911009	AT2G48080	oxidoreductase, 2OG-Fe(II) oxygenase family protein
2.05	11.91	1.04	11.39	0.00457941	AT3G30690	
2.05	12.1	1.03	11.59	0.00459015	AT3G32210	
2.05	7.84	1.03	7.33	0.01829146	AT4G07498	
2.05	12.66	1.03	12.14	0.00563172	AT4G08138	
2.05	12.98	1.04	12.46	0.00646462	AT5G10510	AIL6_PLT3__AINTEGUMENTA-like 6 AIL6_PLT3__AINTEGUMENTA-like 6
2.05	11.26	1.03	10.74	0.01004503	AT5G12880	proline-rich family protein
2.05	10.58	1.03	10.06	0.00266801	AT5G32215	
2.05	11.17	1.04	10.65	0.00756439	AT5G32420	
2.05	10.28	1.04	9.76	0.00791298	AT5G33252	
2.05	9.55	1.04	9.03	0.00677003	AT5G35000	
2.05	14.92	1.04	14.4	0.00461339	ATCG00870	ORF77.1__Protein precursor Ycf15, putative, chloroplast
2.04	14.36	1.03	13.84	0.00426996	AT1G13300	HRS1__myb-like transcription factor family protein
2.04	11.44	1.03	10.93	0.0049944	AT1G17345	SAUR-like auxin-responsive protein family
2.04	10.69	1.03	10.18	0.00546655	AT1G50850	
2.04	13.29	1.03	12.78	0.00422741	AT2G07703	
2.04	10.72	1.03	10.21	0.01112779	AT2G12300	
2.04	10.34	1.03	9.83	0.00458514	AT2G14180	
2.04	12.31	1.03	11.8	0.00479923	AT2G14650	
2.04	13.75	1.03	13.23	0.00309366	AT2G28080	UDP-Glycosyltransferase superfamily protein
2.04	11.76	1.03	11.24	0.01834038	AT2G34020	Calcium-binding EF-hand family protein
2.04	12.65	1.03	12.14	0.00548785	AT4G06507	
2.04	9.73	1.03	9.21	0.00664438	AT4G06543	
2.04	14.38	1.03	13.87	0.00742793	AT4G09030	AGP10_ATAGP10__arabinogalactan protein 10
2.04	11.62	1.03	11.11	0.00508871	AT5G10300	AtHNL_ATMES5_HNL_MES5__methyl esterase 5
2.04	7.48	1.03	6.96	0.0287518	AT5G13220	JAS1_JAZ10_TIFY9__jasmonate-zim-domain protein 10 JAS1_JAZ10_TIFY9__jasmonate-zim-domain protein 10 JAS1_JAZ10_TIFY9__jasmonate-zim-domain protein 10 JAS1_JAZ10_TIFY9__jasmonate-zim-domain protein 10
2.04	12.43	1.03	11.91	0.00494369	AT5G32143	
2.04	11.63	1.03	11.12	0.00341207	AT5G32726	
2.04	11.48	1.03	10.96	0.006315	AT5G35940	Mannose-binding lectin superfamily protein
2.04	9.68	1.03	9.16	0.003464	AT5G45082	
2.04	9.39	1.03	8.88	0.00470122	AT5G66050	Wound-responsive family protein
2.03	10.33	1.02	9.82	0.00830701	AT1G42300	
2.03	14.31	1.02	13.8	0.00709457	AT1G52070	Mannose-binding lectin superfamily protein
2.03	8.34	1.02	7.83	0.01706333	AT1G75020	LPAT4__lysophosphatidyl acyltransferase 4

2.03	10.55	1.02	10.04	0.00467998	AT2G01024	
2.03	10.12	1.02	9.6	0.00372592	AT2G01037	
2.03	12.05	1.02	11.54	0.00597811	AT2G09953	
2.03	12.78	1.02	12.27	0.00449194	AT2G12750	
2.03	10.44	1.02	9.92	0.00508871	AT2G13390	
2.03	13.55	1.02	13.04	0.02585389	AT2G36690	2-oxoglutarate (2OG) and Fe(II)-dependent oxygenase superfamily protein
2.03	13.5	1.02	12.99	0.00603002	AT3G49570	LSU3__response to low sulfur 3
2.03	10.92	1.02	10.41	0.00868459	AT4G06484	
2.03	14.09	1.02	13.58	0.01907535	AT4G08770	Prx37__Peroxidase superfamily protein
2.03	8.49	1.02	7.98	0.00294186	AT4G18360	Aldolase-type TIM barrel family protein
2.03	14.2	1.02	13.68	0.0063438	AT4G21410	CRK29__cysteine-rich RLK (RECEPTOR-like protein kinase) 29
2.03	10.96	1.02	10.45	0.00397758	AT5G31804	
2.03	10.45	1.02	9.94	0.00470122	AT5G32228	
2.03	10.23	1.02	9.72	0.00601931	AT5G32423	
2.03	8.28	1.02	7.76	0.00944571	AT5G32598	
2.03	8.54	1.02	8.03	0.00356968	AT5G34845	
2.03	10.4	1.02	9.89	0.02767043	AT5G58750	NAD(P)-binding Rossmann-fold superfamily protein
2.03	12.89	1.02	12.38	0.01509063	AT5G64870	SPFH/Band 7/PHB domain-containing membrane-associated protein family
2.03	13.03	1.02	12.52	0.01239597	ATMG00680	ORF122C__hypothetical protein
2.02	10.21	1.02	9.7	0.01091381	AT1G06930	unknown protein; Has 4478 Blast hits to 355 proteins in 82 species: Archae - 0; Bacteria - 29; Metazoa - 379; Fungi - 125; Plants - 120; Viruses - 25; Other Eukaryotes - 3800 (source: NCBI BLink).
2.02	13.05	1.01	12.54	0.00356968	AT1G37015	
2.02	11.45	1.01	10.94	0.00962279	AT1G42140	
2.02	12.52	1.01	12.02	0.00922151	AT1G55940	CYP708A1__cytochrome P450, family 708, subfamily A, polypeptide 1
2.02	14.85	1.01	14.34	0.00294186	AT2G06050	AtOPR3_DDE1_OPR3__oxophytodienoate-reductase 3
2.02	10.98	1.01	10.47	0.00508871	AT2G09870	
2.02	10.68	1.02	10.17	0.00528978	AT2G10780	
2.02	11.75	1.01	11.24	0.01156551	AT2G30340	LBD13__LOB domain-containing protein 13
2.02	7.76	1.01	7.26	0.00581265	AT3G23725	
2.02	10.44	1.02	9.94	0.00678457	AT3G24370	
2.02	10.24	1.02	9.74	0.00887205	AT3G32010	
2.02	9.93	1.01	9.43	0.00635402	AT3G32899	
2.02	11.35	1.02	10.84	0.00893397	AT3G33085	
2.02	10.58	1.01	10.07	0.00449194	AT3G42624	
2.02	10.19	1.01	9.69	0.0049944	AT4G06562	

2.02	10.09	1.02	9.58	0.00709862	AT4G06736	
2.02	11.06	1.02	10.55	0.00554683	AT4G08050	
2.02	9.38	1.01	8.88	0.0059467	AT4G26380	Cysteine/Histidine-rich C1 domain family protein
2.02	10.86	1.01	10.35	0.02896469	AT4G28460	unknown protein; FUNCTIONS IN: molecular_function unknown; INVOLVED IN: biological_process unknown; LOCATED IN: endomembrane system; EXPRESSED IN: 10 plant structures; EXPRESSED DURING: LP.04 four leaves visible, 4 anthesis, petal differentiation and expansion stage; Has 8 Blast hits to 8 proteins in 3 species: Archae - 0; Bacteria - 0; Metazoa - 0; Fungi - 0; Plants - 8; Viruses - 0; Other Eukaryotes - 0 (source: NCBI BLink).
2.02	10.3	1.01	9.8	0.0029534	AT4G34460	AGB1_ATAGB1_ELK4__GTP binding protein beta 1
2.02	9.43	1.01	8.92	0.00953179	AT5G34865	
2.02	12.18	1.01	11.67	0.00480388	AT5G38280	PR5K__PR5-like receptor kinase
2.02	12.97	1.01	12.47	0.03517777	AT5G52750	Heavy metal transport/detoxification superfamily protein
2.02	14.23	1.01	13.72	0.00486881	ATCG00320	
2.01	10.01	1.01	9.51	0.00640693	AT1G35600	
2.01	11.39	1.01	10.89	0.00763771	AT1G36540	
2.01	12.52	1.01	12.01	0.00763565	AT1G42605	
2.01	9.7	1.01	9.19	0.03657021	AT1G47990	ATGA2OX4_GA2OX4__gibberellin 2-oxidase 4
2.01	9.31	1	8.81	0.00306866	AT2G06720	
2.01	12.59	1.01	12.09	0.00873067	AT2G07729	
2.01	12.68	1.01	12.18	0.00730315	AT2G07747	
2.01	11.14	1.01	10.63	0.00755929	AT2G12240	
2.01	10.66	1.01	10.15	0.00487538	AT3G09032	unknown protein; BEST Arabidopsis thaliana protein match is: unknown protein (TAIR:AT5G01225.1); Has 35333 Blast hits to 34131 proteins in 2444 species: Archae - 798; Bacteria - 22429; Metazoa - 974; Fungi - 991; Plants - 531; Viruses - 0; Other Eukaryotes - 9610 (source: NCBI BLink).
2.01	9.45	1.01	8.94	0.00636507	AT3G32890	
2.01	10.73	1.01	10.22	0.00483444	AT3G33058	
2.01	12.87	1.01	12.37	0.00508871	AT3G47570	Leucine-rich repeat protein kinase family protein
2.01	12.59	1.01	12.08	0.00717498	AT4G06586	
2.01	11.09	1	10.59	0.00485723	AT4G27460	Cystathionine beta-synthase (CBS) family protein
2.01	12.2	1.01	11.7	0.01159209	AT5G10720	AHK5_CKI2_HK5__histidine kinase 5
2.01	10.84	1.01	10.33	0.00526334	AT5G34985	
-2.01	8.22	-1.01	8.72	0.02208575	AT1G07885	unknown protein; FUNCTIONS IN: molecular_function unknown; INVOLVED IN: biological_process unknown; LOCATED IN: cellular_component unknown; Has 0 Blast hits to 0 proteins in 0 species (source: NCBI BLink).
-2.01	7.96	-1.01	8.46	0.00394838	AT1G22590	AGL87__AGAMOUS-like 87 AGL87__AGAMOUS-like 87

-2.01	8.78	-1.01	9.29	0.01992898	AT1G56165	
-2.01	7.56	-1.01	8.06	0.0436797	AT1G61097	Expressed protein
-2.01	8.26	-1.01	8.76	0.00649867	AT1G67265	DVL3_RTFL21__ROTUNDIFOLIA like 21
-2.01	11.73	-1.01	12.23	0.00470122	AT1G75180	Erythronate-4-phosphate dehydrogenase family protein
-2.01	9.94	-1.01	10.45	0.00433482	AT2G17470	ALMT6_AtALMT6__Aluminium activated malate transporter family protein
-2.01	8.1	-1.01	8.6	0.01457695	AT2G30690	Protein of unknown function, DUF593
-2.01	10.12	-1.01	10.62	0.00829215	AT3G08840	D-alanine--D-alanine ligase family
-2.01	12.53	-1.01	13.03	0.00479295	AT3G11470	4'-phosphopantetheinyl transferase superfamily
-2.01	13.92	-1.01	14.43	0.00435453	AT3G11670	DGD1__UDP-Glycosyltransferase superfamily protein
-2.01	9.21	-1.01	9.71	0.00636507	AT3G13680	F-box and associated interaction domains-containing protein
-2.01	10.85	-1.01	11.35	0.04812464	AT3G30720	QQS__qua-quine starch
-2.01	13.09	-1	13.6	0.00486881	AT4G13493	
-2.01	11.04	-1.01	11.54	0.00470122	AT4G16470	Tetratricopeptide repeat (TPR)-like superfamily protein
-2.01	9.74	-1.01	10.25	0.00660299	AT4G37022	unknown protein; FUNCTIONS IN: molecular_function unknown; INVOLVED IN: biological_process unknown; LOCATED IN: cellular_component unknown; Has 0 Blast hits to 0 proteins in 0 species (source: NCBI BLink).
-2.01	9.85	-1.01	10.36	0.00329522	AT5G10140	AGL25_FLC_FLC_FLF__K-box region and MADS-box transcription factor family protein
-2.01	10	-1.01	10.51	0.0120122	AT5G17780	alpha/beta-Hydrolases superfamily protein
-2.01	7.12	-1.01	7.63	0.00478732	AT5G26345	
-2.01	12.88	-1.01	13.38	0.00625393	AT5G47900	Protein of unknown function (DUF1624) Protein of unknown function (DUF1624) Protein of unknown function (DUF1624)
-2.01	7.21	-1.01	7.71	0.0165446	AT5G64520	ATXRCC2_XRCC2__homolog of X-ray repair cross complementing 2 (XRCC2)
-2.02	8.45	-1.01	8.96	0.01021897	AT1G05330	unknown protein; Has 6 Blast hits to 6 proteins in 2 species: Archae - 0; Bacteria - 0; Metazoa - 0; Fungi - 0; Plants - 6; Viruses - 0; Other Eukaryotes - 0 (source: NCBI BLink).
-2.02	10.59	-1.02	11.1	0.0093984	AT1G32385	
-2.02	13.36	-1.01	13.86	0.0029534	AT1G63360	Disease resistance protein (CC-NBS-LRR class) family
-2.02	14.6	-1.02	15.1	0.00737142	AT1G64900	CYP89_CYP89A2__cytochrome P450, family 89, subfamily A, polypeptide 2
-2.02	12.51	-1.02	13.02	0.00611309	AT1G66920	Protein kinase superfamily protein
-2.02	11.36	-1.02	11.87	0.00480388	AT2G23470	RUS4__Protein of unknown function, DUF647
-2.02	11.56	-1.02	12.07	0.01373422	AT3G07730	unknown protein; BEST Arabidopsis thaliana protein match is: unknown protein (TAIR:AT4G01170.1); Has 1299 Blast hits to 1149 proteins in 206 species: Archae - 3; Bacteria - 100; Metazoa - 453; Fungi - 114; Plants - 108; Viruses - 2; Other Eukaryotes - 519 (source: NCBI BLink).
-2.02	8.21	-1.01	8.72	0.00433482	AT3G48300	CYP71A23__cytochrome P450, family 71, subfamily A, polypeptide 23
-2.02	10.43	-1.02	10.94	0.03148877	AT4G13680	Protein of unknown function (DUF295)
-2.02	11.74	-1.02	12.25	0.00356968	AT4G29400	Protein of unknown function (DUF3531)

-2.02	8.8	-1.01	9.3	0.00309366	AT5G04800	Ribosomal S17 family protein
-2.02	14.73	-1.01	15.23	0.0069202	AT5G24150	SQE5_SQP1__FAD/NAD(P)-binding oxidoreductase family protein SQE5_SQP1__FAD/NAD(P)-binding oxidoreductase family protein
-2.02	13.72	-1.01	14.22	0.00764436	AT5G25130	CYP71B12__cytochrome P450, family 71, subfamily B, polypeptide 12
-2.02	9.39	-1.02	9.9	0.006151	AT5G64250	Aldolase-type TIM barrel family protein
-2.02	8.31	-1.01	8.82	0.0106626	AT5G65130	Integrase-type DNA-binding superfamily protein
-2.03	8.83	-1.02	9.34	0.01590268	AT1G33730	CYP76C5__cytochrome P450, family 76, subfamily C, polypeptide 5
-2.03	13.64	-1.02	14.15	0.00537072	AT1G52590	Putative thiol-disulphide oxidoreductase DCC
-2.03	9.53	-1.02	10.04	0.00526334	AT1G64990	GTG1__GPCR-type G protein 1
-2.03	10.69	-1.02	11.2	0.01984609	AT1G75770	unknown protein; Has 6 Blast hits to 5 proteins in 2 species: Archae - 0; Bacteria - 0; Metazoa - 0; Fungi - 0; Plants - 6; Viruses - 0; Other Eukaryotes - 0 (source: NCBI BLink).
-2.03	12.17	-1.02	12.68	0.02238487	AT2G18120	SRS4__SHI-related sequence 4
-2.03	11.32	-1.02	11.83	0.01375277	AT2G22800	HAT9__Homeobox-leucine zipper protein family
-2.03	9.25	-1.02	9.76	0.01044382	AT2G29680	ATCDC6_CDC6__cell division control 6
-2.03	10.68	-1.02	11.19	0.00542693	AT5G53030	unknown protein; CONTAINS InterPro DOMAIN/s: Protein of unknown function DUF688 (InterPro:IPR007789); BEST Arabidopsis thaliana protein match is: unknown protein (TAIR:AT4G27810.1); Has 1807 Blast hits to 1807 proteins in 277 species: Archae - 0; Bacteria - 0; Metazoa - 736; Fungi - 347; Plants - 385; Viruses - 0; Other Eukaryotes - 339 (source: NCBI BLink). unknown protein; CONTAINS InterPro DOMAIN/s: Protein of unknown function DUF688 (InterPro:IPR007789); BEST Arabidopsis thaliana protein match is: unknown protein (TAIR:AT4G27810.1); Has 1807 Blast hits to 1807 proteins in 277 species: Archae - 0; Bacteria - 0; Metazoa - 736; Fungi - 347; Plants - 385; Viruses - 0; Other Eukaryotes - 339 (source: NCBI BLink).
-2.03	11.16	-1.02	11.67	0.00429467	AT5G59630	
-2.04	8.6	-1.03	9.11	0.02155292	AT1G17780	unknown protein; BEST Arabidopsis thaliana protein match is: unknown protein (TAIR:AT2G16575.1); Has 13 Blast hits to 13 proteins in 2 species: Archae - 0; Bacteria - 0; Metazoa - 0; Fungi - 0; Plants - 13; Viruses - 0; Other Eukaryotes - 0 (source: NCBI BLink).
-2.04	12.04	-1.03	12.56	0.01665892	AT1G20350	ATTIM17-1_TIM17-1__translocase inner membrane subunit 17-1
-2.04	9.2	-1.03	9.71	0.0198452	AT1G48400	F-box/RNI-like/FBD-like domains-containing protein
-2.04	12.79	-1.03	13.3	0.02876339	AT1G65870	Disease resistance-responsive (dirigent-like protein) family protein
-2.04	12.16	-1.03	12.68	0.00294186	AT2G04230	FBD, F-box and Leucine Rich Repeat domains containing protein
-2.04	9.52	-1.03	10.04	0.00449194	AT2G21187	
-2.04	13.03	-1.03	13.54	0.00886809	AT3G06145	unknown protein; Has 15 Blast hits to 15 proteins in 7 species: Archae - 0; Bacteria - 0; Metazoa - 0; Fungi - 0; Plants - 15; Viruses - 0; Other Eukaryotes - 0 (source: NCBI BLink).
-2.04	13.42	-1.03	13.94	0.00547874	AT3G14770	AtSWEET2_SWEET2__Nodulin MtN3 family protein
-2.04	11.94	-1.03	12.45	0.00539445	AT3G15115	unknown protein; BEST Arabidopsis thaliana protein match is: unknown protein

						(TAIR:AT1G53180.1); Has 47 Blast hits to 47 proteins in 15 species: Archae - 0; Bacteria - 0; Metazoa - 13; Fungi - 0; Plants - 30; Viruses - 0; Other Eukaryotes - 4 (source: NCBI BLink).
-2.04	13.84	-1.03	14.35	0.00597898	AT3G22420	ATWNK2_WNK2_ZIK3__with no lysine (K) kinase 2
-2.04	11.93	-1.03	12.44	0.00814975	AT3G60160	ABCC9_ATMRP9_MRP9__multidrug resistance-associated protein 9
-2.04	8.81	-1.03	9.32	0.04595734	AT4G01490	
-2.04	14.03	-1.03	14.54	0.02464648	AT4G26150	CGA1_GATA22_GNL__cytokinin-responsive gata factor 1
-2.04	13.03	-1.03	13.54	0.00356968	AT4G35290	ATGLR3.2_ATGLUR2_GLR3.2_GLUR2__glutamate receptor 2
-2.04	11.88	-1.03	12.4	0.01962777	AT5G16080	AtCXE17_CXE17__carboxyesterase 17
-2.04	7.84	-1.03	8.35	0.01273993	AT5G28360	
-2.04	13.3	-1.03	13.81	0.00627482	AT5G47900	Protein of unknown function (DUF1624) Protein of unknown function (DUF1624) Protein of unknown function (DUF1624)
-2.04	13.61	-1.03	14.12	0.00561713	AT5G54630	zinc finger protein-related
-2.04	14.16	-1.03	14.68	0.00455949	AT5G56850	unknown protein; EXPRESSED IN: 21 plant structures; EXPRESSED DURING: 13 growth stages; Has 35333 Blast hits to 34131 proteins in 2444 species: Archae - 798; Bacteria - 22429; Metazoa - 974; Fungi - 991; Plants - 531; Viruses - 0; Other Eukaryotes - 9610 (source: NCBI BLink). unknown protein; EXPRESSED IN: 21 plant structures; EXPRESSED DURING: 13 growth stages; Has 35333 Blast hits to 34131 proteins in 2444 species: Archae - 798; Bacteria - 22429; Metazoa - 974; Fungi - 991; Plants - 531; Viruses - 0; Other Eukaryotes - 9610 (source: NCBI BLink).
-2.05	9.06	-1.04	9.58	0.01123603	AT1G29490	SAUR-like auxin-responsive protein family
-2.05	9.16	-1.04	9.68	0.01227536	AT1G55545	
-2.05	10.41	-1.04	10.93	0.01671458	AT1G78750	F-box/RNI-like superfamily protein
-2.05	12.18	-1.03	12.7	0.00518233	AT2G01918	PQL3__PsbQ-like 3
-2.05	7.48	-1.04	7.99	0.01850719	AT2G26692	
-2.05	11.14	-1.03	11.66	0.00489929	AT2G29910	F-box/RNI-like superfamily protein
-2.05	8.81	-1.03	9.33	0.04552858	AT3G02515	
-2.05	12.64	-1.04	13.16	0.01887253	AT4G24700	unknown protein; Has 20 Blast hits to 20 proteins in 7 species: Archae - 0; Bacteria - 0; Metazoa - 0; Fungi - 0; Plants - 20; Viruses - 0; Other Eukaryotes - 0 (source: NCBI BLink).
-2.05	11.55	-1.04	12.07	0.00784132	AT5G01260	Carbohydrate-binding-like fold Carbohydrate-binding-like fold
-2.05	11.53	-1.03	12.05	0.00490678	AT5G10946	unknown protein; FUNCTIONS IN: molecular_function unknown; INVOLVED IN: biological_process unknown; LOCATED IN: endomembrane system; Has 30201 Blast hits to 17322 proteins in 780 species: Archae - 12; Bacteria - 1396; Metazoa - 17338; Fungi - 3422; Plants - 5037; Viruses - 0; Other Eukaryotes - 2996 (source: NCBI BLink).
-2.05	13.56	-1.03	14.08	0.04014525	AT5G15948	CPuORF10__conserved peptide upstream open reading frame 10
-2.05	12.46	-1.04	12.98	0.00433482	AT5G46390	Peptidase S41 family protein
-2.05	9.81	-1.04	10.32	0.00766115	AT5G49380	CONTAINS InterPro DOMAIN/s: Protein of unknown function DUF625 (InterPro:IPR006887);

-2.05	10.6	-1.03	11.12	0.00950083	AT5G61670	BEST Arabidopsis thaliana protein match is: binding (TAIR:AT3G06670.1); Has 1807 Blast hits to 1807 proteins in 277 species: Archae - 0; Bacteria - 0; Metazoa - 736; Fungi - 347; Plants - 385; Viruses - 0; Other Eukaryotes - 339 (source: NCBI BLink). Encodes a close homolog of the Cauliflower OR (Orange) protein. The function of OR is to induce the differentiation of proplastids or other noncolored plastids into chromoplasts for carotenoid accumulation. Both proteins contain a Cysteine-rich zinc finger domain that is highly specific to DnaJ-like molecular chaperons.
-2.06	13.01	-1.04	13.53	0.00714664	AT1G03400	2-oxoglutarate (2OG) and Fe(II)-dependent oxygenase superfamily protein
-2.06	11.93	-1.04	12.45	0.01649691	AT1G48100	Pectin lyase-like superfamily protein
-2.06	13.42	-1.04	13.94	0.00514085	AT1G71810	Protein kinase superfamily protein
-2.06	10.03	-1.04	10.55	0.00426996	AT2G12460	
-2.06	11.07	-1.05	11.6	0.03067696	AT2G20880	AtERF53_ERF53__Integrase-type DNA-binding superfamily protein
-2.06	13.2	-1.04	13.72	0.0033501	AT2G26215	
-2.06	13.49	-1.04	14.01	0.00447426	AT2G32860	BGLU33__beta glucosidase 33
-2.06	8.38	-1.04	8.9	0.04273327	AT3G21380	Mannose-binding lectin superfamily protein
-2.06	12.11	-1.04	12.63	0.00446653	AT4G13880	AtRLP48_RLP48__receptor like protein 48
-2.06	9.05	-1.04	9.57	0.00788752	AT5G07380	unknown protein; FUNCTIONS IN: molecular_function unknown; INVOLVED IN: biological_process unknown; LOCATED IN: cellular_component unknown; EXPRESSED IN: egg cell; Has 84 Blast hits to 81 proteins in 31 species: Archae - 0; Bacteria - 0; Metazoa - 42; Fungi - 0; Plants - 41; Viruses - 0; Other Eukaryotes - 1 (source: NCBI BLink).
-2.06	12.57	-1.04	13.09	0.00508663	AT5G63060	Sec14p-like phosphatidylinositol transfer family protein
-2.07	10.81	-1.05	11.33	0.00873067	AT1G08810	AtMYB60_MYB60__myb domain protein 60 AtMYB60_MYB60__myb domain protein 60
-2.07	13.67	-1.05	14.19	0.0182935	AT1G27480	alpha/beta-Hydrolases superfamily protein
-2.07	11.51	-1.05	12.04	0.0052996	AT2G27770	Plant protein of unknown function (DUF868)
-2.07	10.7	-1.05	11.22	0.04980088	AT2G38210	PDX1L4__putative PDX1-like protein 4
-2.07	10.34	-1.05	10.86	0.01920193	AT2G40670	ARR16_RR16__response regulator 16
-2.07	8.39	-1.05	8.92	0.00459015	AT3G05620	Plant invertase/pectin methylesterase inhibitor superfamily
-2.07	10.34	-1.05	10.87	0.00655859	AT3G26085	CAAX amino terminal protease family protein
-2.07	10.82	-1.05	11.34	0.00508871	AT3G51075	
-2.07	8.19	-1.05	8.71	0.00508663	AT4G23250	CRK17_DUF26-21_EMB1290_RKC1__kinases;protein kinases
-2.07	11.52	-1.05	12.05	0.00731488	AT4G25300	2-oxoglutarate (2OG) and Fe(II)-dependent oxygenase superfamily protein
-2.07	9.91	-1.05	10.43	0.03662507	AT5G45630	Protein of unknown function, DUF584
-2.07	10.95	-1.05	11.47	0.02078417	AT5G53030	unknown protein; CONTAINS InterPro DOMAIN/s: Protein of unknown function DUF688 (InterPro:IPR007789); BEST Arabidopsis thaliana protein match is: unknown protein (TAIR:AT4G27810.1); Has 1807 Blast hits to 1807 proteins in 277 species: Archae - 0; Bacteria - 0; Metazoa - 736; Fungi - 347; Plants - 385; Viruses - 0; Other Eukaryotes - 339 (source: NCBI

						BLink). unknown protein; CONTAINS InterPro DOMAIN/s: Protein of unknown function DUF688 (InterPro:IPR007789); BEST Arabidopsis thaliana protein match is: unknown protein (TAIR:AT4G27810.1); Has 1807 Blast hits to 1807 proteins in 277 species: Archae - 0; Bacteria - 0; Metazoa - 736; Fungi - 347; Plants - 385; Viruses - 0; Other Eukaryotes - 339 (source: NCBI BLink).
-2.08	9.43	-1.06	9.95	0.01672072	AT1G02470	Polyketide cyclase/dehydrase and lipid transport superfamily protein
-2.08	13.79	-1.05	14.32	0.00424195	AT1G43790	TED6__tracheary element differentiation-related 6
-2.08	8.74	-1.06	9.26	0.03713676	AT1G65960	GAD2__glutamate decarboxylase 2
-2.08	8.39	-1.06	8.92	0.0067964	AT1G69540	AGL94__AGAMOUS-like 94
-2.08	12.11	-1.05	12.64	0.00869468	AT1G70985	hydroxyproline-rich glycoprotein family protein
-2.08	11.68	-1.06	12.21	0.00253321	AT2G15080	AtRLP19_RLP19__receptor like protein 19
-2.08	9.45	-1.05	9.97	0.04753624	AT3G02670	Glycine-rich protein family
-2.08	15.02	-1.06	15.55	0.00518233	AT4G19170	CCD4_NCED4__nine-cis-epoxycarotenoid dioxygenase 4
-2.08	9.04	-1.06	9.56	0.01048515	AT4G31398	
-2.08	11.21	-1.05	11.73	0.00413041	AT4G34060	DML3__demeter-like protein 3 DML3__demeter-like protein 3
-2.08	13.84	-1.05	14.37	0.00601693	AT5G21100	Plant L-ascorbate oxidase
-2.08	11.46	-1.06	11.99	0.00760062	AT5G45830	ATDOG1_DOG1_GSQ5__delay of germination 1
-2.08	10.55	-1.06	11.08	0.01083634	AT5G52740	Copper transport protein family
-2.08	10.59	-1.05	11.11	0.00512535	AT5G59200	OTP80__Tetratricopeptide repeat (TPR)-like superfamily protein
-2.09	9.01	-1.07	9.55	0.00951091	AT1G73445	
-2.09	13.61	-1.07	14.14	0.00608255	AT1G76570	Chlorophyll A-B binding family protein
-2.09	12.88	-1.07	13.41	0.00413041	AT2G04790	unknown protein; FUNCTIONS IN: molecular_function unknown; INVOLVED IN: biological_process unknown; EXPRESSED IN: 17 plant structures; EXPRESSED DURING: 11 growth stages; Has 33 Blast hits to 33 proteins in 10 species: Archae - 0; Bacteria - 0; Metazoa - 0; Fungi - 0; Plants - 33; Viruses - 0; Other Eukaryotes - 0 (source: NCBI BLink). unknown protein; FUNCTIONS IN: molecular_function unknown; INVOLVED IN: biological_process unknown; EXPRESSED IN: 17 plant structures; EXPRESSED DURING: 11 growth stages; Has 33 Blast hits to 33 proteins in 10 species: Archae - 0; Bacteria - 0; Metazoa - 0; Fungi - 0; Plants - 33; Viruses - 0; Other Eukaryotes - 0 (source: NCBI BLink). unknown protein; FUNCTIONS IN: molecular_function unknown; INVOLVED IN: biological_process unknown; EXPRESSED IN: 17 plant structures; EXPRESSED DURING: 11 growth stages; Has 33 Blast hits to 33 proteins in 10 species: Archae - 0; Bacteria - 0; Metazoa - 0; Fungi - 0; Plants - 33; Viruses - 0; Other Eukaryotes - 0 (source: NCBI BLink). unknown protein; FUNCTIONS IN: molecular_function unknown; INVOLVED IN: biological_process unknown; EXPRESSED IN: 17 plant structures; EXPRESSED DURING: 11 growth stages; Has 33 Blast hits to 33 proteins in 10 species: Archae - 0; Bacteria - 0; Metazoa - 0; Fungi - 0; Plants - 33; Viruses - 0; Other Eukaryotes - 0 (source:

						NCBI BLink).
-2.09	13.24	-1.06	13.77	0.00288784	AT2G16380	Sec14p-like phosphatidylinositol transfer family protein
-2.09	10.88	-1.06	11.41	0.00619816	AT2G42920	Pentatricopeptide repeat (PPR-like) superfamily protein
-2.09	10.08	-1.06	10.61	0.00896923	AT3G24518	
-2.09	8.64	-1.07	9.17	0.04564272	AT3G25180	CYP82G1__cytochrome P450, family 82, subfamily G, polypeptide 1
-2.09	13.7	-1.06	14.23	0.01186197	AT3G48720	DCF__HXXXD-type acyl-transferase family protein
-2.09	9.61	-1.07	10.14	0.00323071	AT4G05130	ATENT4_ENT4__equilibrative nucleoside transporter 4
-2.09	8.38	-1.07	8.91	0.00459015	AT4G18375	RNA-binding KH domain-containing protein
-2.09	10.11	-1.06	10.64	0.003464	AT4G29670	ACHT2__atypical CYS HIS rich thioredoxin 2
						SQE5_SQP1__FAD/NAD(P)-binding oxidoreductase family protein
-2.09	14.71	-1.06	15.24	0.00801286	AT5G24150	SQE5_SQP1__FAD/NAD(P)-binding oxidoreductase family protein
-2.09	9.39	-1.06	9.92	0.02498187	AT5G46830	ATNIG1_NIG1__NACL-inducible gene 1
-2.09	8.42	-1.06	8.95	0.00891472	AT5G52000	IMPA-8__importin alpha isoform 8
-2.1	12.03	-1.07	12.56	0.00617912	AT1G09490	NAD(P)-binding Rossmann-fold superfamily protein
-2.1	11.65	-1.07	12.18	0.0050256	AT2G04270	RNEE/G__RNAse E/G-like
-2.1	14.57	-1.07	15.11	0.00353373	AT2G36630	Sulfite exporter TauE/SafE family protein
-2.1	9.23	-1.07	9.76	0.02867752	AT3G18540	
-2.1	14.15	-1.07	14.69	0.00356968	AT3G50270	HXXXD-type acyl-transferase family protein
-2.1	13.49	-1.07	14.03	0.00356968	AT3G53720	ATCHX20_CHX20__cation/H+ exchanger 20
-2.1	9.75	-1.07	10.29	0.00671183	AT5G04830	Nuclear transport factor 2 (NTF2) family protein
-2.1	11.9	-1.07	12.43	0.01394525	AT5G10930	CIPK5_SnRK3.24__CBL-interacting protein kinase 5
-2.1	11.78	-1.07	12.31	0.00638451	AT5G40060	Disease resistance protein (NBS-LRR class) family
-2.1	9.77	-1.07	10.31	0.02266246	AT5G40140	RING/U-box superfamily protein with ARM repeat domain
-2.11	11.17	-1.07	11.71	0.00519318	AT1G04650	unknown protein; Has 30201 Blast hits to 17322 proteins in 780 species: Archae - 12; Bacteria - 1396; Metazoa - 17338; Fungi - 3422; Plants - 5037; Viruses - 0; Other Eukaryotes - 2996 (source: NCBI BLink).
-2.11	12.47	-1.08	13.01	0.00525619	AT1G05900	ATNTH2_NTH2__endonuclease III 2 ATNTH2_NTH2__endonuclease III 2
-2.11	12.98	-1.08	13.52	0.00873067	AT1G76110	HMG (high mobility group) box protein with ARID/BRIGHT DNA-binding domain
-2.11	9.72	-1.08	10.26	0.0085162	AT1G76520	Auxin efflux carrier family protein
-2.11	9.71	-1.08	10.25	0.00717498	AT3G04960	Domain of unknown function (DUF3444)
-2.11	10.5	-1.08	11.03	0.00356968	AT3G28130	nodulin MtN21 /EamA-like transporter family protein
-2.11	10.5	-1.08	11.04	0.02679699	AT3G53140	O-methyltransferase family protein
-2.11	14.5	-1.08	15.04	0.003464	AT4G00750	S-adenosyl-L-methionine-dependent methyltransferases superfamily protein
-2.11	13.44	-1.08	13.97	0.00514085	AT4G13800	Protein of unknown function (DUF803)
-2.11	11.55	-1.08	12.09	0.02613032	AT5G02190	ATASP38_EMB24_PCS1__Eukaryotic aspartyl protease family protein
-2.11	8.06	-1.08	8.6	0.00476616	AT5G26970	unknown protein; Has 1807 Blast hits to 1807 proteins in 277 species: Archae - 0; Bacteria - 0;

						Metazoa - 736; Fungi - 347; Plants - 385; Viruses - 0; Other Eukaryotes - 339 (source: NCBI BLink).
-2.11	9.13	-1.08	9.67	0.00479701	AT5G34790	
-2.12	14.01	-1.08	14.55	0.00753361	AT1G13650	BEST Arabidopsis thaliana protein match is: 18S pre-ribosomal assembly protein gar2-related (TAIR:AT2G03810.4); Has 3688 Blast hits to 1629 proteins in 255 species: Archae - 22; Bacteria - 222; Metazoa - 684; Fungi - 292; Plants - 62; Viruses - 14; Other Eukaryotes - 2392 (source: NCBI BLink). BEST Arabidopsis thaliana protein match is: 18S pre-ribosomal assembly protein gar2-related (TAIR:AT2G03810.4); Has 3688 Blast hits to 1629 proteins in 255 species: Archae - 22; Bacteria - 222; Metazoa - 684; Fungi - 292; Plants - 62; Viruses - 14; Other Eukaryotes - 2392 (source: NCBI BLink).
-2.12	12.43	-1.08	12.97	0.01025493	AT1G15510	ATECB2_ECB2_VAC1__Tetratricopeptide repeat (TPR)-like superfamily protein
-2.12	10.96	-1.09	11.5	0.01229243	AT1G43590	
-2.12	11.14	-1.09	11.68	0.01499456	AT1G77230	Tetratricopeptide repeat (TPR)-like superfamily protein
-2.12	13.14	-1.09	13.68	0.00439438	AT2G22980	SCPL13__serine carboxypeptidase-like 13 SCPL13__serine carboxypeptidase-like 13
-2.12	11.31	-1.08	11.85	0.02266799	AT2G45350	CRR4__Pentatricopeptide repeat (PPR) superfamily protein
-2.12	13.25	-1.08	13.79	0.00532052	AT4G02420	Concanavalin A-like lectin protein kinase family protein
-2.12	12.09	-1.08	12.63	0.00494369	AT4G12917	
-2.12	9.88	-1.09	10.43	0.03482313	AT4G34740	ASE2_ATASE2_ATPURF2_CIA1__GLN phosphoribosyl pyrophosphate amidotransferase 2
-2.12	8.75	-1.08	9.29	0.01922232	AT5G14260	Rubisco methyltransferase family protein
-2.12	8.86	-1.08	9.4	0.0106426	AT5G44980	F-box/RNI-like/FBD-like domains-containing protein
-2.12	13.05	-1.08	13.59	0.00339076	AT5G46500	BEST Arabidopsis thaliana protein match is: disease resistance protein (TIR-NBS-LRR class) family (TAIR:AT5G46260.1); Has 1807 Blast hits to 1807 proteins in 277 species: Archae - 0; Bacteria - 0; Metazoa - 736; Fungi - 347; Plants - 385; Viruses - 0; Other Eukaryotes - 339 (source: NCBI BLink).
-2.13	6.91	-1.09	7.45	0.00422741	AT1G62935	unknown protein; FUNCTIONS IN: molecular_function unknown; INVOLVED IN: biological_process unknown; LOCATED IN: endomembrane system; Has 45 Blast hits to 45 proteins in 13 species: Archae - 0; Bacteria - 2; Metazoa - 6; Fungi - 0; Plants - 8; Viruses - 0; Other Eukaryotes - 29 (source: NCBI BLink).
-2.13	12.4	-1.09	12.95	0.00488717	AT1G68530	CER6_CUT1_G2_KCS6_POP1__3-ketoacyl-CoA synthase 6
-2.13	8.6	-1.09	9.15	0.01160115	AT1G68580	agenet domain-containing protein / bromo-adjacent homology (BAH) domain-containing protein
-2.13	7.51	-1.09	8.05	0.01011114	AT3G04717	
-2.13	13.19	-1.09	13.74	0.00610922	AT3G24480	Leucine-rich repeat (LRR) family protein
-2.13	12.95	-1.09	13.49	0.00592108	AT3G27540	beta-1,4-N-acetylglucosaminyltransferase family protein
-2.13	7.12	-1.09	7.67	0.03252261	AT3G28890	AtRLP43_RLP43__receptor like protein 43
-2.13	14.18	-1.09	14.72	0.00508871	AT4G25290	DNA photolyases;DNA photolyases
-2.13	12.87	-1.09	13.42	0.00571104	AT5G46240	KAT1__potassium channel in Arabidopsis thaliana 1

-2.13	9.81	-1.09	10.35	0.03492906	AT5G60910	AGL8_FUL__AGAMOUS-like 8
-2.14	8.59	-1.1	9.14	0.01595663	AT1G55800	Domain of unknown function (DUF2431)
-2.14	7.37	-1.09	7.92	0.00796998	AT1G78710	TBL42__TRICHOME BIREFRINGENCE-LIKE 42
-2.14	11.63	-1.1	12.18	0.04799243	AT3G05936	unknown protein; FUNCTIONS IN: molecular_function unknown; INVOLVED IN: biological_process unknown; LOCATED IN: cellular_component unknown; Has 30201 Blast hits to 17322 proteins in 780 species: Archae - 12; Bacteria - 1396; Metazoa - 17338; Fungi - 3422; Plants - 5037; Viruses - 0; Other Eukaryotes - 2996 (source: NCBI BLink).
-2.14	9.11	-1.1	9.66	0.0070566	AT3G13404	unknown protein; FUNCTIONS IN: molecular_function unknown; INVOLVED IN: biological_process unknown; LOCATED IN: cellular_component unknown; Has 0 Blast hits to 0 proteins in 0 species (source: NCBI BLink).
-2.14	13.72	-1.1	14.27	0.00302324	AT3G17640	Leucine-rich repeat (LRR) family protein
-2.14	13.69	-1.1	14.24	0.01093028	AT3G47430	PEX11B__peroxin 11B
-2.14	9.52	-1.1	10.07	0.00769902	AT3G51070	S-adenosyl-L-methionine-dependent methyltransferases superfamily protein
-2.14	12.91	-1.1	13.46	0.00637266	AT5G47900	Protein of unknown function (DUF1624) Protein of unknown function (DUF1624) Protein of unknown function (DUF1624)
-2.14	9.05	-1.1	9.6	0.00603083	AT5G51910	TCP family transcription factor
-2.14	13.35	-1.1	13.89	0.0069202	AT5G56860	GATA21_GNC__GATA type zinc finger transcription factor family protein
-2.15	8.38	-1.1	8.93	0.01711614	AT1G30250	unknown protein; Has 5 Blast hits to 5 proteins in 2 species: Archae - 0; Bacteria - 0; Metazoa - 0; Fungi - 0; Plants - 5; Viruses - 0; Other Eukaryotes - 0 (source: NCBI BLink).
-2.15	14.21	-1.11	14.76	0.00459015	AT1G70410	ATBCA4_BCA4_CA4__beta carbonic anhydrase 4 ATBCA4_BCA4_CA4__beta carbonic anhydrase 4
-2.15	12.15	-1.11	12.7	0.00438643	AT2G41210	PIP5K5__phosphatidylinositol- 4-phosphate 5-kinase 5
-2.15	11.08	-1.11	11.63	0.00294186	AT3G28080	nodulin MtN21 /EamA-like transporter family protein nodulin MtN21 /EamA-like transporter family protein
-2.15	10.49	-1.11	11.05	0.00356968	AT3G53170	Tetratricopeptide repeat (TPR)-like superfamily protein
-2.15	11.2	-1.1	11.75	0.00655859	AT3G58070	GIS__C2H2 and C2HC zinc fingers superfamily protein
-2.16	9.09	-1.11	9.65	0.01144885	AT1G18990	Protein of unknown function, DUF593
-2.16	12.79	-1.11	13.35	0.0078464	AT1G21060	Protein of unknown function, DUF547
-2.16	12.3	-1.11	12.86	0.00614592	AT1G66230	AtMYB20_MYB20__myb domain protein 20
-2.16	8.72	-1.11	9.27	0.00927499	AT2G03520	ATUPS4_UPS4__ureide permease 4
-2.16	11.41	-1.11	11.97	0.01608511	AT2G32490	
-2.16	13.89	-1.11	14.45	0.003464	AT2G32880	TRAF-like family protein
-2.16	9.64	-1.11	10.2	0.00787677	AT2G38185	RING/U-box superfamily protein
-2.16	13.71	-1.11	14.26	0.00781333	AT2G41040	S-adenosyl-L-methionine-dependent methyltransferases superfamily protein
-2.16	9.49	-1.11	10.04	0.00394474	AT2G45800	PLIM2a__GATA type zinc finger transcription factor family protein PLIM2a__GATA type zinc finger transcription factor family protein

-2.16	10.62	-1.11	11.17	0.00978611	AT3G27860	Tudor/PWWP/MBT superfamily protein
-2.16	13.27	-1.11	13.83	0.00882166	AT4G32980	ATH1__homeobox gene 1
-2.16	12.44	-1.11	12.99	0.00974823	AT4G36870	BLH2_SAW1__BEL1-like homeodomain 2
-2.16	12.67	-1.11	13.23	0.00309366	AT5G03260	LAC11__laccase 11
-2.16	8.48	-1.11	9.04	0.00565467	AT5G59760	Protein of unknown function (DUF1635)
-2.16	13.24	-1.11	13.79	0.00417125	AT5G61270	PIF7__phytochrome-interacting factor7 PIF7__phytochrome-interacting factor7
-2.17	9.67	-1.12	10.23	0.00428727	AT1G10155	ATPP2-A10_PP2-A10__phloem protein 2-A10
-2.17	10.54	-1.12	11.1	0.00760062	AT1G15165	RING/FYVE/PHD zinc finger superfamily protein
-2.17	12.4	-1.12	12.96	0.00873067	AT1G28230	ATPUP1_PUP1__purine permease 1
-2.17	8.94	-1.12	9.5	0.01371693	AT1G49370	
-2.17	12.15	-1.12	12.71	0.0029534	AT1G75380	ATBBD1_BBD1__bifunctional nuclease in basal defense response 1
-2.17	12.94	-1.12	13.5	0.00680864	AT3G14810	MSL5__mechanosensitive channel of small conductance-like 5
-2.17	12.66	-1.12	13.22	0.02140313	AT3G19850	Phototropic-responsive NPH3 family protein
-2.17	11.07	-1.12	11.63	0.00664061	AT3G20200	Protein kinase protein with adenine nucleotide alpha hydrolases-like domain
-2.17	11.63	-1.12	12.19	0.02536149	AT3G29670	PMAT2__HXXXD-type acyl-transferase family protein
-2.17	13.89	-1.12	14.45	0.00581265	AT3G48320	CYP71A21__cytochrome P450, family 71, subfamily A, polypeptide 21
-2.17	8.9	-1.11	9.45	0.036036	AT3G49480	Protein with RNI-like/FBD-like domains
-2.17	8.81	-1.12	9.37	0.00473458	AT4G09920	FBD, F-box and Leucine Rich Repeat domains containing protein
-2.17	7.98	-1.12	8.53	0.00478534	AT5G02650	BEST Arabidopsis thaliana protein match is: Telomerase activating protein Est1 (TAIR:AT1G28260.2); Has 20 Blast hits to 20 proteins in 2 species: Archae - 0; Bacteria - 0; Metazoa - 0; Fungi - 0; Plants - 20; Viruses - 0; Other Eukaryotes - 0 (source: NCBI BLink).
-2.17	7.71	-1.12	8.26	0.01635632	AT5G07600	Oleosin family protein
-2.17	12.67	-1.11	13.23	0.00438828	AT5G41050	Pollen Ole e 1 allergen and extensin family protein
-2.17	12.56	-1.12	13.12	0.00411275	AT5G43870	Plant protein of unknown function (DUF828) with plant pleckstrin homology-like region
-2.18	9.26	-1.12	9.82	0.04126741	AT1G01680	ATPUB54_PUB54__plant U-box 54
-2.18	10.64	-1.12	11.2	0.00422741	AT1G31790	Tetratricopeptide repeat (TPR)-like superfamily protein
-2.18	9.21	-1.12	9.77	0.00610609	AT1G55370	NDF5__NDH-dependent cyclic electron flow 5
-2.18	13.7	-1.13	14.26	0.00266801	AT1G62630	Disease resistance protein (CC-NBS-LRR class) family
-2.18	12.18	-1.13	12.74	0.01879782	AT2G24160	
-2.18	11.57	-1.12	12.13	0.00251625	AT4G01650	Polyketide cyclase / dehydrase and lipid transport protein
-2.18	14.12	-1.13	14.69	0.003464	AT4G09350	CRRJ_NdhT__Chaperone DnaJ-domain superfamily protein
-2.18	12.39	-1.12	12.95	0.03515165	AT5G47330	alpha/beta-Hydrolases superfamily protein
-2.18	13.37	-1.12	13.94	0.00549226	AT5G64380	Inositol monophosphatase family protein
-2.19	9.54	-1.13	10.11	0.00511666	AT1G77992	
-2.19	13.25	-1.13	13.81	0.01267872	AT2G40520	Nucleotidyltransferase family protein
-2.19	8.27	-1.13	8.84	0.00973078	AT2G47670	Plant invertase/pectin methylesterase inhibitor superfamily protein

-2.19	9.21	-1.13	9.78	0.00219513	AT3G20865	AGP40__arabinogalactan protein 40
-2.19	13.41	-1.13	13.98	0.01666891	AT3G46670	UGT76E11__UDP-glucosyl transferase 76E11
-2.19	10.62	-1.13	11.19	0.003464	AT4G05631	unknown protein; Has 30201 Blast hits to 17322 proteins in 780 species: Archae - 12; Bacteria - 1396; Metazoa - 17338; Fungi - 3422; Plants - 5037; Viruses - 0; Other Eukaryotes - 2996 (source: NCBI BLink).
-2.19	10.61	-1.13	11.17	0.00833976	AT5G19140	AILP1_ATAILP1__Aluminium induced protein with YGL and LRDR motifs
-2.19	10.07	-1.13	10.63	0.01671447	AT5G28960	unknown protein; FUNCTIONS IN: molecular_function unknown; INVOLVED IN: biological_process unknown; LOCATED IN: endomembrane system; BEST Arabidopsis thaliana protein match is: unknown protein (TAIR:AT5G28910.2); Has 30201 Blast hits to 17322 proteins in 780 species: Archae - 12; Bacteria - 1396; Metazoa - 17338; Fungi - 3422; Plants - 5037; Viruses - 0; Other Eukaryotes - 2996 (source: NCBI BLink).
-2.2	12.29	-1.14	12.85	0.00449194	AT1G24147	unknown protein; FUNCTIONS IN: molecular_function unknown; INVOLVED IN: biological_process unknown; LOCATED IN: endomembrane system; BEST Arabidopsis thaliana protein match is: unknown protein (TAIR:AT1G24148.1); Has 4 Blast hits to 4 proteins in 2 species: Archae - 0; Bacteria - 0; Metazoa - 0; Fungi - 0; Plants - 4; Viruses - 0; Other Eukaryotes - 0 (source: NCBI BLink).
-2.2	8.93	-1.14	9.5	0.00309366	AT1G29780	Haloacid dehalogenase-like hydrolase (HAD) superfamily protein
-2.2	7.68	-1.14	8.25	0.00507483	AT1G51680	4CL.1_4CL1_AT4CL1__4-coumarate:CoA ligase 1
-2.2	13.1	-1.14	13.66	0.02139563	AT1G75460	ATP-dependent protease La (LON) domain protein
-2.2	11.23	-1.14	11.8	0.00664061	AT3G28150	AXY4L_TBL22__TRICHOME BIREFRINGENCE-LIKE 22
-2.2	11.24	-1.13	11.81	0.0177665	AT5G15845	
-2.2	13.48	-1.14	14.05	0.01239929	AT5G19850	alpha/beta-Hydrolases superfamily protein
-2.2	7.6	-1.13	8.17	0.0201658	AT5G27080	AtCDC20.3_CDC20.3__Transducin family protein / WD-40 repeat family protein
-2.2	11.47	-1.14	12.04	0.01786402	AT5G51410	LUC7 N_terminus domain-containing protein
-2.21	11.64	-1.14	12.21	0.00663139	AT1G01420	UGT72B3__UDP-glucosyl transferase 72B3
-2.21	10.32	-1.14	10.89	0.0126018	AT1G29110	Cysteine proteinases superfamily protein
-2.21	10.34	-1.15	10.91	0.01488948	AT1G67890	PAS domain-containing protein tyrosine kinase family protein
-2.21	14.66	-1.15	15.23	0.00442816	AT2G32540	ATCSLB04_ATCSLB4_CSLB04_CSLB04__cellulose synthase-like B4
-2.21	12.33	-1.15	12.9	0.00306866	AT2G34960	CAT5__cationic amino acid transporter 5
-2.21	11.32	-1.14	11.89	0.0039427	AT3G58160	ATMYOS3_ATXIJ_MYA3_XI-16_XIJ__P-loop containing nucleoside triphosphate hydrolases superfamily protein
-2.21	7.73	-1.14	8.3	0.00514085	AT4G10695	CDC68-related
-2.21	11.54	-1.14	12.11	0.00873067	AT4G16680	P-loop containing nucleoside triphosphate hydrolases superfamily protein
-2.21	10.25	-1.15	10.82	0.00356968	AT4G19560	CYCT1;2__Cyclin family protein
-2.21	9.85	-1.14	10.42	0.0204015	AT4G23310	CRK23__cysteine-rich RLK (RECEPTOR-like protein kinase) 23
-2.21	10	-1.14	10.57	0.00886775	AT5G17580	Phototropic-responsive NPH3 family protein

-2.21	12.66	-1.15	13.23	0.01177152	AT5G65890	ACR1__ACT domain repeat 1 ACR1__ACT domain repeat 1
-2.22	9.56	-1.15	10.13	0.00294186	AT1G13220	CRWN2_LINC2__nuclear matrix constituent protein-related
-2.22	11.99	-1.15	12.56	0.00309366	AT1G21580	Zinc finger C-x8-C-x5-C-x3-H type family protein
-2.22	12.9	-1.15	13.48	0.00322354	AT1G26230	Cpn60beta4__TCP-1/cpn60 chaperonin family protein
-2.22	11.34	-1.15	11.91	0.00426996	AT1G31300	TRAM, LAG1 and CLN8 (TLC) lipid-sensing domain containing protein
-2.22	13.24	-1.15	13.82	0.01571536	AT1G53870	Protein of unknown function (DUF567) Protein of unknown function (DUF567)
-2.22	11.91	-1.15	12.48	0.00535046	AT1G73700	MATE efflux family protein
-2.22	12.32	-1.15	12.89	0.00709926	AT2G18560	UDP-Glycosyltransferase superfamily protein
-2.22	12.02	-1.15	12.6	0.00499347	AT2G38460	ATIREG1_FPN1_IREG1__iron regulated 1
-2.22	7.75	-1.15	8.32	0.01559265	AT3G05930	GLP8__germin-like protein 8
-2.22	9.46	-1.15	10.04	0.04646494	AT3G06125	
-2.22	11.73	-1.15	12.3	0.00309366	AT3G28080	nodulin MtN21 /EamA-like transporter family protein nodulin MtN21 /EamA-like transporter family protein
-2.22	12.24	-1.15	12.82	0.019243	AT3G50560	NAD(P)-binding Rossmann-fold superfamily protein
-2.22	9.08	-1.15	9.66	0.0062393	AT4G05000	VPS28-1_VPS28-2__Vacuolar protein sorting-associated protein VPS28 family protein
-2.22	12.7	-1.15	13.27	0.00403113	AT4G24810	Protein kinase superfamily protein
-2.22	8.64	-1.15	9.22	0.04778423	AT4G28280	LLG3__LORELEI-LIKE-GPI ANCHORED PROTEIN 3
-2.22	10.31	-1.15	10.89	0.01171532	AT5G15254	unknown protein; Has 30201 Blast hits to 17322 proteins in 780 species: Archae - 12; Bacteria - 1396; Metazoa - 17338; Fungi - 3422; Plants - 5037; Viruses - 0; Other Eukaryotes - 2996 (source: NCBI BLink).
-2.22	10.65	-1.15	11.23	0.01673621	AT5G26742	emb1138__DEAD box RNA helicase (RH3)
-2.23	13.27	-1.16	13.85	0.00329522	AT1G66910	Protein kinase superfamily protein
-2.23	13.53	-1.16	14.1	0.00486057	AT1G73870	BBX16_B-box type zinc finger protein with CCT domain
-2.23	8.2	-1.16	8.77	0.03762207	AT3G21500	DXPS1__1-deoxy-D-xylulose 5-phosphate synthase 1 DXPS1__1-deoxy-D-xylulose 5-phosphate synthase 1
-2.23	11.68	-1.16	12.26	0.00836735	AT3G63370	OTP86__Tetratricopeptide repeat (TPR)-like superfamily protein
-2.24	12.83	-1.16	13.41	0.01391174	AT1G16730	UP6__unknown protein 6
-2.24	8.2	-1.16	8.78	0.01001532	AT1G51490	BGLU36__beta glucosidase 36
-2.24	11.26	-1.16	11.85	0.01740623	AT1G52942	
-2.24	10.72	-1.16	11.3	0.00778833	AT1G75360	unknown protein; Has 74 Blast hits to 71 proteins in 15 species: Archae - 0; Bacteria - 4; Metazoa - 4; Fungi - 0; Plants - 54; Viruses - 0; Other Eukaryotes - 12 (source: NCBI BLink).
-2.24	7.95	-1.16	8.54	0.00294186	AT2G05518	
-2.24	12.64	-1.16	13.22	0.00294186	AT2G17695	FUNCTIONS IN: molecular_function unknown; INVOLVED IN: biological_process unknown; CONTAINS InterPro DOMAIN/s: Domain of unknown function DUF1990 (InterPro:IPR018960).
-2.24	12.18	-1.17	12.77	0.0103816	AT2G32640	Lycopene beta/epsilon cyclase protein
-2.24	12.73	-1.16	13.31	0.01048515	AT3G47580	Leucine-rich repeat protein kinase family protein

-2.24	12.99	-1.17	13.57	0.00961332	AT3G53310	AP2/B3-like transcriptional factor family protein
-2.24	11.21	-1.16	11.79	0.00518233	AT4G24040	ATTRE1_TRE1__trehalase 1
-2.24	8.88	-1.16	9.46	0.03172961	AT5G04210	CCCH-type zinc fingerfamily protein with RNA-binding domain
-2.24	12.2	-1.16	12.78	0.00497286	AT5G39030	Protein kinase superfamily protein
-2.25	8.64	-1.17	9.22	0.00543099	AT1G32583	FUNCTIONS IN: molecular_function unknown; INVOLVED IN: biological_process unknown; LOCATED IN: endomembrane system; BEST Arabidopsis thaliana protein match is: tapetum determinant 1 (TAIR:AT4G24972.1); Has 35333 Blast hits to 34131 proteins in 2444 species: Archae - 798; Bacteria - 22429; Metazoa - 974; Fungi - 991; Plants - 531; Viruses - 0; Other Eukaryotes - 9610 (source: NCBI BLink).
-2.25	8.08	-1.17	8.66	0.00516629	AT1G73965	CLE13__CLAVATA3/ESR-RELATED 13
-2.25	8.64	-1.17	9.23	0.00507786	AT2G03410	Mo25 family protein
-2.25	9.53	-1.17	10.11	0.02998937	AT2G17780	MCA2__PLAC8 family protein
-2.25	12.86	-1.17	13.44	0.00435164	AT3G56630	CYP94D2__cytochrome P450, family 94, subfamily D, polypeptide 2
-2.25	12.83	-1.17	13.42	0.02357463	AT4G00050	UNE10__basic helix-loop-helix (bHLH) DNA-binding superfamily protein
-2.25	10.47	-1.17	11.06	0.00518233	AT5G44490	FBD, F-box, Skp2-like and Leucine Rich Repeat domains containing protein
-2.26	13.37	-1.18	13.96	0.00309366	AT1G31920	Tetratricopeptide repeat (TPR)-like superfamily protein
-2.26	11.19	-1.18	11.78	0.00294186	AT1G45545	Plant protein of unknown function (DUF827)
-2.26	12.5	-1.18	13.09	0.01363319	AT3G21760	HYR1__UDP-Glycosyltransferase superfamily protein
-2.26	9.91	-1.18	10.5	0.01927749	AT3G47030	F-box and associated interaction domains-containing protein
-2.26	12.36	-1.17	12.95	0.00413041	AT4G25280	P-loop containing nucleoside triphosphate hydrolases superfamily protein
-2.26	9.25	-1.18	9.84	0.00963031	AT5G23480	SWIB/MDM2 domain;Plus-3;GYF
-2.26	11.44	-1.17	12.03	0.04967263	AT5G24200	alpha/beta-Hydrolases superfamily protein
-2.26	14.67	-1.17	15.26	0.00251625	AT5G49740	ATFRO7_FRO7__ferric reduction oxidase 7
-2.26	9.77	-1.18	10.36	0.00394838	AT5G57180	CIA2__chloroplast import apparatus 2
-2.27	10.91	-1.18	11.5	0.00911009	AT1G29610	
-2.27	12.55	-1.18	13.14	0.00338136	AT1G29720	Leucine-rich repeat transmembrane protein kinase Leucine-rich repeat transmembrane protein kinase
-2.27	11.77	-1.18	12.36	0.011023	AT2G23180	CYP96A1__cytochrome P450, family 96, subfamily A, polypeptide 1
-2.27	10.74	-1.18	11.33	0.00394474	AT2G31751	
-2.27	13.32	-1.18	13.91	0.00633393	AT3G09650	CRM3_HCF152__Tetratricopeptide repeat (TPR)-like superfamily protein
-2.27	10.66	-1.18	11.25	0.00635475	AT3G10420	SPD1__P-loop containing nucleoside triphosphate hydrolases superfamily protein
-2.27	8.82	-1.18	9.41	0.00561713	AT4G05497	RNI-like superfamily protein
-2.27	13.04	-1.18	13.63	0.00663139	AT4G25780	CAP (Cysteine-rich secretory proteins, Antigen 5, and Pathogenesis-related 1 protein) superfamily protein
-2.27	8.67	-1.19	9.26	0.0039802	AT4G39420	unknown protein; INVOLVED IN: biological_process unknown; LOCATED IN: cellular_component unknown; EXPRESSED IN: leaf; EXPRESSED DURING: LP.04 four leaves

						visible, LP.02 two leaves visible; Has 20 Blast hits to 19 proteins in 8 species: Archae - 0; Bacteria - 0; Metazoa - 0; Fungi - 0; Plants - 20; Viruses - 0; Other Eukaryotes - 0 (source: NCBI BLink).
-2.27	9.01	-1.18	9.6	0.02406437	AT5G38240	Protein kinase family protein
-2.28	8.55	-1.19	9.14	0.01258171	AT1G34580	Major facilitator superfamily protein
-2.28	13.7	-1.19	14.3	0.00221781	AT1G61300	LRR and NB-ARC domains-containing disease resistance protein
-2.28	8.1	-1.19	8.69	0.01040772	AT3G02310	AGL4_SEP2__K-box region and MADS-box transcription factor family protein
-2.28	12.09	-1.19	12.69	0.01093028	AT4G01030	pentatricopeptide (PPR) repeat-containing protein
-2.28	10.56	-1.19	11.16	0.0052996	AT4G21940	CPK15__calcium-dependent protein kinase 15
-2.28	13.82	-1.19	14.41	0.00404197	AT4G23260	CRK18__cysteine-rich RLK (RECEPTOR-like protein kinase) 18
-2.28	10.23	-1.19	10.82	0.03348677	AT5G03620	Subtilisin-like serine endopeptidase family protein
-2.28	7.43	-1.19	8.03	0.03874697	AT5G35798	
-2.28	11.77	-1.19	12.36	0.00356968	AT5G45840	Leucine-rich repeat protein kinase family protein
-2.28	9.04	-1.19	9.63	0.00442816	AT5G64490	ARM repeat superfamily protein
-2.28	13.57	-1.19	14.16	0.0178121	AT5G67370	Protein of unknown function (DUF1230)
-2.29	11.83	-1.19	12.43	0.02424045	AT1G21240	WAK3__wall associated kinase 3
-2.29	10.59	-1.2	11.18	0.00459015	AT1G29715	
-2.29	11.27	-1.2	11.87	0.00738846	AT2G18260	ATSYP112_SYP112__syntaxin of plants 112
-2.29	11.51	-1.19	12.11	0.01637674	AT4G38960	BBX19_B-box type zinc finger family protein B-box type zinc finger family protein
-2.29	12.4	-1.2	13	0.00470122	AT5G08070	TCP17__TCP domain protein 17
-2.29	8.94	-1.2	9.53	0.00266801	AT5G34800	
-2.3	10.25	-1.2	10.85	0.00520191	AT1G26600	CLE9__CLAVATA3/ESR-RELATED 9
-2.3	7.91	-1.2	8.51	0.03903368	AT1G69570	Dof-type zinc finger DNA-binding family protein
-2.3	12.47	-1.2	13.08	0.00514085	AT2G22980	SCPL13__serine carboxypeptidase-like 13 SCPL13__serine carboxypeptidase-like 13
-2.3	10.31	-1.2	10.92	0.00461339	AT2G24010	scpl23__serine carboxypeptidase-like 23
-2.3	8.95	-1.2	9.55	0.01263033	AT3G14710	RNI-like superfamily protein
-2.3	7.94	-1.2	8.54	0.00728048	AT3G21500	DXPS1__1-deoxy-D-xylulose 5-phosphate synthase 1 DXPS1__1-deoxy-D-xylulose 5-phosphate synthase 1
-2.3	14.64	-1.2	15.24	0.00398975	AT3G26280	CYP71B4__cytochrome P450, family 71, subfamily B, polypeptide 4
-2.3	12.46	-1.2	13.06	0.00693255	AT4G21380	ARK3_RK3__receptor kinase 3
-2.3	12.9	-1.2	13.5	0.01821024	AT5G19580	glyoxal oxidase-related protein
-2.3	10.61	-1.2	11.21	0.00905135	AT5G37410	Family of unknown function (DUF577)
-2.3	12.62	-1.2	13.22	0.00999044	AT5G60970	TCP5__TEOSINTE BRANCHED 1, cycloidea and PCF transcription factor 5
-2.31	11.99	-1.21	12.59	0.00999489	AT1G31290	AGO3__ARGONAUTE 3
-2.31	11.97	-1.21	12.57	0.00323071	AT1G54790	GDSL-like Lipase/Acylhydrolase superfamily protein GDSL-like Lipase/Acylhydrolase superfamily protein
-2.31	8.76	-1.21	9.37	0.00568994	AT1G70270	unknown protein; BEST Arabidopsis thaliana protein match is: unknown protein

						(TAIR:AT1G23965.1); Has 20 Blast hits to 20 proteins in 7 species: Archae - 0; Bacteria - 0; Metazoa - 0; Fungi - 0; Plants - 20; Viruses - 0; Other Eukaryotes - 0 (source: NCBI BLink).
-2.31	9.3	-1.21	9.91	0.00470122	AT1G77570	Winged helix-turn-helix transcription repressor DNA-binding
-2.31	13.15	-1.21	13.76	0.003464	AT2G19650	Cysteine/Histidine-rich C1 domain family protein
-2.31	8.9	-1.21	9.5	0.01895664	AT2G35990	LOG2__Putative lysine decarboxylase family protein
-2.31	11.95	-1.21	12.55	0.02621334	AT3G22100	basic helix-loop-helix (bHLH) DNA-binding superfamily protein
-2.31	12.22	-1.21	12.83	0.00309366	AT3G61870	unknown protein; FUNCTIONS IN: molecular_function unknown; INVOLVED IN: biological_process unknown; LOCATED IN: chloroplast, chloroplast inner membrane, chloroplast envelope; EXPRESSED IN: 23 plant structures; EXPRESSED DURING: 14 growth stages; Has 200 Blast hits to 200 proteins in 73 species: Archae - 0; Bacteria - 112; Metazoa - 0; Fungi - 0; Plants - 53; Viruses - 0; Other Eukaryotes - 35 (source: NCBI BLink).
-2.31	9.81	-1.21	10.41	0.00229082	AT4G02005	
-2.31	12.37	-1.21	12.98	0.00367141	AT4G15810	P-loop containing nucleoside triphosphate hydrolases superfamily protein
-2.31	11.04	-1.21	11.64	0.00567193	AT5G64780	Uncharacterised conserved protein UCP009193
-2.32	11.03	-1.21	11.64	0.003464	AT1G54730	Major facilitator superfamily protein
-2.32	12.71	-1.21	13.32	0.01273557	AT1G59720	CRR28__Tetratricopeptide repeat (TPR)-like superfamily protein
-2.32	10.51	-1.21	11.12	0.0358738	AT3G50570	hydroxyproline-rich glycoprotein family protein
-2.32	9.83	-1.21	10.43	0.00593427	AT3G60970	ABCC15_ATMRP15_MRP15__multidrug resistance-associated protein 15
-2.32	10.26	-1.22	10.87	0.00314069	AT5G48220	Aldolase-type TIM barrel family protein
-2.32	7.43	-1.22	8.04	0.01217614	AT5G48550	F-box associated ubiquitination effector family protein
-2.33	9.15	-1.22	9.76	0.00315341	AT3G52820	ATPAP22_PAP22__purple acid phosphatase 22
-2.33	8.8	-1.22	9.41	0.00494369	AT4G13494	
-2.33	12.21	-1.22	12.82	0.02378688	AT5G20670	Protein of unknown function (DUF1677)
-2.33	7.06	-1.22	7.68	0.00610609	AT5G64800	CLE21__CLAVATA3/ESR-RELATED 21
-2.34	8.63	-1.22	9.24	0.00294186	AT1G70410	ATBCA4_BCA4_CA4__beta carbonic anhydrase 4 ATBCA4_BCA4_CA4__beta carbonic anhydrase 4
-2.34	10.54	-1.22	11.15	0.00732204	AT2G13570	NF-YB7__nuclear factor Y, subunit B7
-2.34	7.69	-1.23	8.31	0.0112008	AT2G23672	
-2.34	12.35	-1.23	12.96	0.02060718	AT2G25680	MOT1__molybdate transporter 1
-2.34	9.96	-1.23	10.58	0.00671183	AT2G30540	Thioredoxin superfamily protein
-2.34	12.74	-1.23	13.35	0.00508871	AT2G32550	Cell differentiation, Rcd1-like protein
-2.34	11.74	-1.23	12.35	0.00686655	AT3G09162	unknown protein; FUNCTIONS IN: molecular_function unknown; INVOLVED IN: biological_process unknown; LOCATED IN: endomembrane system; EXPRESSED IN: stem, root, inflorescence, cultured cell, leaf; Has 35333 Blast hits to 34131 proteins in 2444 species: Archae - 798; Bacteria - 22429; Metazoa - 974; Fungi - 991; Plants - 531; Viruses - 0; Other Eukaryotes - 9610 (source: NCBI BLink).

-2.34	9.25	-1.22	9.86	0.00508663	AT3G09550	Ankyrin repeat family protein
-2.34	9.27	-1.23	9.88	0.00458514	AT3G20160	Terpenoid synthases superfamily protein
-2.34	12.68	-1.23	13.3	0.02266246	AT3G25010	AtRLP41_RLP41__receptor like protein 41
-2.34	10.14	-1.23	10.75	0.00603875	AT4G38760	Protein of unknown function (DUF3414)
-2.34	9.13	-1.23	9.74	0.004055	AT5G20450	FUNCTIONS IN: molecular_function unknown; INVOLVED IN: biological_process unknown; LOCATED IN: cellular_component unknown; EXPRESSED IN: 17 plant structures; EXPRESSED DURING: 9 growth stages; CONTAINS InterPro DOMAIN/s: Dilute (InterPro:IPR002710); BEST Arabidopsis thaliana protein match is: myosin, putative (TAIR:AT5G20470.1).
-2.34	11.85	-1.23	12.46	0.00569479	AT5G61950	Ubiquitin carboxyl-terminal hydrolase-related protein
-2.35	11.46	-1.23	12.08	0.00565467	AT1G11190	BFN1_ENDO1__bifunctional nuclease i
-2.35	10.25	-1.23	10.86	0.01700016	AT1G50180	NB-ARC domain-containing disease resistance protein
-2.35	11.51	-1.23	12.13	0.0043694	AT3G28960	Transmembrane amino acid transporter family protein
-2.35	8.63	-1.24	9.25	0.00738846	AT5G24520	ATTTG1_TTG_TTG1_URM23__Transducin/WD40 repeat-like superfamily protein
-2.36	10.23	-1.24	10.85	0.00300845	AT1G54680	unknown protein; FUNCTIONS IN: molecular_function unknown; INVOLVED IN: biological_process unknown; LOCATED IN: endomembrane system; EXPRESSED IN: 22 plant structures; EXPRESSED DURING: 13 growth stages; BEST Arabidopsis thaliana protein match is: unknown protein (TAIR:AT5G27290.1).
-2.36	9.54	-1.24	10.16	0.01141224	AT1G56170	ATHAP5B_HAP5B_NF-YC2__nuclear factor Y, subunit C2
-2.36	9.34	-1.24	9.96	0.01001317	AT2G27500	Glycosyl hydrolase superfamily protein
-2.36	9.15	-1.24	9.77	0.00546572	AT2G46572	
-2.36	10.01	-1.24	10.63	0.00326511	AT3G04485	
-2.36	14.37	-1.24	15	0.00459015	AT4G12830	alpha/beta-Hydrolases superfamily protein
-2.36	9.66	-1.24	10.28	0.00877158	AT4G39440	
-2.37	13.93	-1.24	14.55	0.00298853	AT1G65190	Protein kinase superfamily protein
-2.37	12.42	-1.24	13.05	0.00417125	AT2G32160	S-adenosyl-L-methionine-dependent methyltransferases superfamily protein S-adenosyl-L-methionine-dependent methyltransferases superfamily protein
-2.37	13.21	-1.24	13.83	0.003464	AT3G18500	DNAse I-like superfamily protein
-2.37	11.21	-1.25	11.83	0.01332569	AT3G49668	
-2.37	8.54	-1.25	9.16	0.0489117	AT4G16855	unknown protein; LOCATED IN: endomembrane system; Has 30201 Blast hits to 17322 proteins in 780 species: Archae - 12; Bacteria - 1396; Metazoa - 17338; Fungi - 3422; Plants - 5037; Viruses - 0; Other Eukaryotes - 2996 (source: NCBI BLink).
-2.38	13.44	-1.25	14.07	0.01512606	AT1G35710	Protein kinase family protein with leucine-rich repeat domain
-2.38	9.1	-1.25	9.72	0.00467998	AT2G35075	unknown protein; Has 30201 Blast hits to 17322 proteins in 780 species: Archae - 12; Bacteria - 1396; Metazoa - 17338; Fungi - 3422; Plants - 5037; Viruses - 0; Other Eukaryotes - 2996 (source: NCBI BLink).

-2.38	10.58	-1.25	11.2	0.00479295	AT4G15660	Thioredoxin superfamily protein
-2.38	9.76	-1.25	10.39	0.00394838	AT5G22240	ATOFP10_OFP10__Ovate family protein
-2.39	10.3	-1.26	10.93	0.04674606	AT1G10586	basic helix-loop-helix (bHLH) DNA-binding superfamily protein basic helix-loop-helix (bHLH) DNA-binding superfamily protein
-2.39	14.13	-1.26	14.76	0.02227861	AT1G64500	Glutaredoxin family protein
-2.39	12.37	-1.26	13	0.00420969	AT3G05932	
-2.39	10.32	-1.25	10.95	0.00824002	AT3G24900	AtRLP39_RLP39__receptor like protein 39
-2.39	8.24	-1.26	8.87	0.003464	AT3G28120	unknown protein; FUNCTIONS IN: molecular_function unknown; INVOLVED IN: biological_process unknown; LOCATED IN: endomembrane system; Has 0 Blast hits to 0 proteins in 0 species (source: NCBI BLink).
-2.39	12.14	-1.26	12.77	0.0052996	AT3G57190	PrfB3__peptide chain release factor, putative
-2.4	8.92	-1.26	9.55	0.00294186	AT1G11735	
-2.4	10.99	-1.27	11.62	0.00486881	AT1G17610	Disease resistance protein (TIR-NBS class)
-2.4	9.54	-1.26	10.17	0.00733931	AT1G58227	
-2.4	8.63	-1.26	9.26	0.00778937	AT2G22810	ACC4_ACS4_ATACS4__1-aminocyclopropane-1-carboxylate synthase 4
-2.4	9.37	-1.27	10	0.00528978	AT2G33280	Major facilitator superfamily protein
-2.4	10.25	-1.27	10.89	0.00756439	AT3G23050	AXR2_IAA7__indole-3-acetic acid 7
-2.4	11.33	-1.27	11.96	0.01159209	AT3G57680	Peptidase S41 family protein
-2.4	12.84	-1.26	13.47	0.00601931	AT4G29070	Phospholipase A2 family protein
-2.4	13.14	-1.26	13.77	0.00486881	AT5G25120	CYP71B11__ytochrome p450, family 71, subfamily B, polypeptide 11
-2.41	9.26	-1.27	9.9	0.00518233	AT3G06985	LCR44__low-molecular-weight cysteine-rich 44
-2.41	10.72	-1.27	11.35	0.00514085	AT3G28140	RNA ligase/cyclic nucleotide phosphodiesterase family protein RNA ligase/cyclic nucleotide phosphodiesterase family protein
-2.42	11.27	-1.28	11.9	0.0067932	AT1G33720	CYP76C6__cytochrome P450, family 76, subfamily C, polypeptide 6
-2.42	11.49	-1.27	12.12	0.00309366	AT1G62400	HT1__Protein kinase superfamily protein
-2.42	10.94	-1.27	11.58	0.00494369	AT1G68935	unknown protein; Has 0 Blast hits to 0 proteins in 0 species (source: NCBI BLink).
-2.42	8.56	-1.27	9.19	0.00753143	AT3G13270	
-2.42	12.29	-1.27	12.93	0.01608511	AT3G19620	Glycosyl hydrolase family protein
-2.42	14.47	-1.28	15.11	0.00836735	AT4G12320	CYP706A6__cytochrome P450, family 706, subfamily A, polypeptide 6
-2.42	13.71	-1.28	14.35	0.00627604	AT4G22200	AKT2_AKT2/3_AKT3_KT2/3__potassium transport 2/3
-2.42	10.02	-1.27	10.66	0.00686073	AT5G01260	Carbohydrate-binding-like fold Carbohydrate-binding-like fold
-2.42	12.82	-1.27	13.46	0.00382427	AT5G46490	Disease resistance protein (TIR-NBS-LRR class) family Disease resistance protein (TIR-NBS-LRR class) family
-2.43	12.9	-1.28	13.53	0.00718811	AT2G29940	ABCG31_ATPDR3_PDR3__pleiotropic drug resistance 3
-2.43	9.68	-1.28	10.32	0.00638451	AT3G06550	RWA2__O-acetyltransferase family protein
-2.43	10.5	-1.28	11.14	0.02461507	AT3G62950	Thioredoxin superfamily protein

-2.43	8.77	-1.28	9.41	0.01442197	AT5G53900	Serine/threonine-protein kinase WNK (With No Lysine)-related
-2.44	10.66	-1.29	11.3	0.00309366	AT1G18750	AGL65__AGAMOUS-like 65
-2.44	11.76	-1.28	12.4	0.006151	AT2G03530	ATUPS2_UPS2__ureide permease 2 ATUPS2_UPS2__ureide permease 2
-2.44	12.28	-1.29	12.93	0.00424195	AT2G32160	S-adenosyl-L-methionine-dependent methyltransferases superfamily protein S-adenosyl-L-methionine-dependent methyltransferases superfamily protein
-2.44	11.76	-1.29	12.4	0.00459015	AT4G38850	ATSAUR15_SAUR-AC1_SAUR15_SAUR_AC1__SAUR-like auxin-responsive protein family
-2.44	9.62	-1.29	10.26	0.01944073	AT5G62040	BFT__PEBP (phosphatidylethanolamine-binding protein) family protein
-2.45	12.27	-1.29	12.92	0.00749234	AT1G05690	BT3__BTB and TAZ domain protein 3
-2.45	12.5	-1.29	13.15	0.00817702	AT2G24850	TAT_TAT3__tyrosine aminotransferase 3
-2.45	7.63	-1.29	8.27	0.011188	AT2G39460	ATRPL23A_RPL23A_RPL23A1_RPL23AA__ribosomal protein L23AA
-2.46	10.21	-1.3	10.86	0.0473296	AT1G30220	ATINT2_INT2__inositol transporter 2
-2.46	12.05	-1.3	12.7	0.00514085	AT1G34060	Pyridoxal phosphate (PLP)-dependent transferases superfamily protein
-2.46	11.95	-1.3	12.6	0.0029534	AT1G54790	GDSL-like Lipase/Acylhydrolase superfamily protein GDSL-like Lipase/Acylhydrolase superfamily protein
-2.46	7.41	-1.3	8.06	0.01004503	AT2G30630	P-loop containing nucleoside triphosphate hydrolases superfamily protein
-2.46	13.69	-1.3	14.34	0.00351857	AT3G48200	unknown protein; FUNCTIONS IN: molecular_function unknown; INVOLVED IN: biological_process unknown; LOCATED IN: endomembrane system; EXPRESSED IN: 21 plant structures; EXPRESSED DURING: 13 growth stages; Has 210 Blast hits to 148 proteins in 42 species: Archae - 0; Bacteria - 118; Metazoa - 0; Fungi - 0; Plants - 48; Viruses - 0; Other Eukaryotes - 44 (source: NCBI BLink).
-2.46	8.36	-1.3	9.01	0.00294186	AT4G20440	smB__small nuclear ribonucleoprotein associated protein B
-2.46	12.16	-1.3	12.81	0.01007972	AT4G37980	ATCAD7_CAD7_ELI3_ELI3-1__elicitor-activated gene 3-1
-2.46	11.55	-1.3	12.2	0.00580337	AT5G50800	AtSWEET13_SWEET13__Nodulin MtN3 family protein
-2.47	13.37	-1.31	14.02	0.02369912	AT1G20070	unknown protein; FUNCTIONS IN: molecular_function unknown; INVOLVED IN: biological_process unknown; LOCATED IN: chloroplast; EXPRESSED IN: 20 plant structures; EXPRESSED DURING: 11 growth stages; Has 26 Blast hits to 26 proteins in 9 species: Archae - 0; Bacteria - 0; Metazoa - 0; Fungi - 2; Plants - 24; Viruses - 0; Other Eukaryotes - 0 (source: NCBI BLink).
-2.47	9.35	-1.31	10	0.02130288	AT1G34760	GF14 OMICRON_GRF11_RHS5__general regulatory factor 11
-2.48	9.43	-1.31	10.09	0.00929952	AT1G02770	Protein of unknown function (DUF626)
-2.48	12.24	-1.31	12.89	0.00479295	AT1G11340	S-locus lectin protein kinase family protein
-2.48	9.89	-1.31	10.54	0.00576204	AT1G53160	SPL4__squamosa promoter binding protein-like 4 SPL4__squamosa promoter binding protein-like 4
-2.48	8.77	-1.31	9.43	0.00409662	AT2G20100	basic helix-loop-helix (bHLH) DNA-binding superfamily protein
-2.48	11.92	-1.31	12.57	0.01067907	AT2G32870	TRAF-like family protein
-2.48	11.42	-1.31	12.08	0.00462463	AT2G47750	GH3.9__putative indole-3-acetic acid-amido synthetase GH3.9

-2.48	9.81	-1.31	10.46	0.00470122	AT3G26160	CYP71B17__cytochrome P450, family 71, subfamily B, polypeptide 17
-2.48	10.04	-1.31	10.7	0.01471306	AT5G09820	Plastid-lipid associated protein PAP / fibrillin family protein
-2.49	9.02	-1.31	9.68	0.00394474	AT1G17030	unknown protein; FUNCTIONS IN: molecular_function unknown; INVOLVED IN: biological_process unknown; LOCATED IN: endomembrane system; BEST Arabidopsis thaliana protein match is: unknown protein (TAIR:AT2G47010.2); Has 70 Blast hits to 70 proteins in 13 species: Archae - 0; Bacteria - 0; Metazoa - 0; Fungi - 0; Plants - 69; Viruses - 0; Other Eukaryotes - 1 (source: NCBI BLink).
-2.49	9.19	-1.32	9.85	0.04381863	AT2G18180	Sec14p-like phosphatidylinositol transfer family protein
-2.49	13.32	-1.31	13.98	0.00528978	AT2G32530	ATCSLB03_ATCSLB3_CSLB03__cellulose synthase-like B3
-2.49	13.4	-1.32	14.06	0.003464	AT2G35130	Tetratricopeptide repeat (TPR)-like superfamily protein
-2.49	9.22	-1.32	9.88	0.04563834	AT3G14630	CYP72A9__cytochrome P450, family 72, subfamily A, polypeptide 9
-2.49	11.29	-1.31	11.94	0.00536471	AT3G62500	BEST Arabidopsis thaliana protein match is: SET domain protein 14 (TAIR:AT3G61740.1); Has 66 Blast hits to 66 proteins in 11 species: Archae - 0; Bacteria - 0; Metazoa - 0; Fungi - 0; Plants - 66; Viruses - 0; Other Eukaryotes - 0 (source: NCBI BLink).
-2.49	8.85	-1.32	9.51	0.01691591	AT5G14980	alpha/beta-Hydrolases superfamily protein
-2.5	9.65	-1.32	10.31	0.00640968	AT1G15990	ATCNGC7_CNGC7__cyclic nucleotide gated channel 7
-2.5	10.53	-1.32	11.19	0.00367141	AT1G23080	ATPIN7_PIN7__Auxin efflux carrier family protein
-2.5	9.67	-1.32	10.33	0.00664061	AT1G73325	Kunitz family trypsin and protease inhibitor protein
-2.51	10.53	-1.33	11.2	0.0029534	AT1G08810	AtMYB60_MYB60__myb domain protein 60 AtMYB60_MYB60__myb domain protein 60
-2.51	7.95	-1.33	8.61	0.04683806	AT1G61320	FBD / Leucine Rich Repeat domains containing protein
-2.51	11.7	-1.33	12.37	0.00315341	AT3G26320	CYP71B36__cytochrome P450, family 71, subfamily B, polypeptide 36
-2.51	14.59	-1.33	15.26	0.00294186	AT4G10120	ATSPS4F__Sucrose-phosphate synthase family protein
-2.51	13.15	-1.33	13.81	0.00565467	AT5G04200	AtMC9_AtMCP2f_MC9_MCP2f__metacaspase 9
-2.51	12.72	-1.32	13.38	0.01290416	AT5G06790	unknown protein; FUNCTIONS IN: molecular_function unknown; INVOLVED IN: biological_process unknown; LOCATED IN: chloroplast; EXPRESSED IN: 9 plant structures; EXPRESSED DURING: LP.04 four leaves visible, LP.02 two leaves visible, petal differentiation and expansion stage, D bilateral stage; BEST Arabidopsis thaliana protein match is: unknown protein (TAIR:AT3G57950.1); Has 1807 Blast hits to 1807 proteins in 277 species: Archae - 0; Bacteria - 0; Metazoa - 736; Fungi - 347; Plants - 385; Viruses - 0; Other Eukaryotes - 339 (source: NCBI BLink).
-2.52	12.2	-1.33	12.86	0.0044091	AT1G29720	Leucine-rich repeat transmembrane protein kinase Leucine-rich repeat transmembrane protein kinase
-2.52	11.79	-1.34	12.46	0.00787353	AT1G47600	BGLU34_TGG4__beta glucosidase 34
-2.52	11.84	-1.33	12.51	0.00326047	AT1G68600	Aluminium activated malate transporter family protein
-2.52	12.85	-1.33	13.52	0.00938576	AT2G36970	UDP-Glycosyltransferase superfamily protein
-2.52	8.78	-1.33	9.45	0.01909056	AT3G22436	unknown protein; Has 30201 Blast hits to 17322 proteins in 780 species: Archae - 12; Bacteria -

						1396; Metazoa - 17338; Fungi - 3422; Plants - 5037; Viruses - 0; Other Eukaryotes - 2996 (source: NCBI BLink).
-2.52	8.89	-1.34	9.56	0.0134514	AT3G27831	Gamma-thionin family protein
-2.52	10.57	-1.33	11.24	0.00507786	AT3G32410	BEST Arabidopsis thaliana protein match is: Actin-binding FH2 (Formin Homology) protein (TAIR:AT2G25050.2); Has 30201 Blast hits to 17322 proteins in 780 species: Archae - 12; Bacteria - 1396; Metazoa - 17338; Fungi - 3422; Plants - 5037; Viruses - 0; Other Eukaryotes - 2996 (source: NCBI BLink).
-2.52	13.24	-1.34	13.91	0.00424195	AT3G42628	phosphoenolpyruvate carboxylase-related / PEP carboxylase-related
-2.52	8.82	-1.34	9.49	0.03826368	AT5G20370	serine-rich protein-related
-2.53	12.71	-1.34	13.38	0.00973078	AT1G18265	Protein of unknown function, DUF593
-2.53	8.88	-1.34	9.55	0.01488948	AT1G20015	
-2.53	8.44	-1.34	9.11	0.00973549	AT1G64820	MATE efflux family protein
-2.53	10.49	-1.34	11.16	0.01004503	AT2G04790	unknown protein; FUNCTIONS IN: molecular_function unknown; INVOLVED IN: biological_process unknown; EXPRESSED IN: 17 plant structures; EXPRESSED DURING: 11 growth stages; Has 33 Blast hits to 33 proteins in 10 species: Archae - 0; Bacteria - 0; Metazoa - 0; Fungi - 0; Plants - 33; Viruses - 0; Other Eukaryotes - 0 (source: NCBI BLink). unknown protein; FUNCTIONS IN: molecular_function unknown; INVOLVED IN: biological_process unknown; EXPRESSED IN: 17 plant structures; EXPRESSED DURING: 11 growth stages; Has 33 Blast hits to 33 proteins in 10 species: Archae - 0; Bacteria - 0; Metazoa - 0; Fungi - 0; Plants - 33; Viruses - 0; Other Eukaryotes - 0 (source: NCBI BLink). unknown protein; FUNCTIONS IN: molecular_function unknown; INVOLVED IN: biological_process unknown; EXPRESSED IN: 17 plant structures; EXPRESSED DURING: 11 growth stages; Has 33 Blast hits to 33 proteins in 10 species: Archae - 0; Bacteria - 0; Metazoa - 0; Fungi - 0; Plants - 33; Viruses - 0; Other Eukaryotes - 0 (source: NCBI BLink). unknown protein; FUNCTIONS IN: molecular_function unknown; INVOLVED IN: biological_process unknown; EXPRESSED IN: 17 plant structures; EXPRESSED DURING: 11 growth stages; Has 33 Blast hits to 33 proteins in 10 species: Archae - 0; Bacteria - 0; Metazoa - 0; Fungi - 0; Plants - 33; Viruses - 0; Other Eukaryotes - 0 (source: NCBI BLink).
-2.53	7.84	-1.34	8.51	0.0070479	AT3G54070	Ankyrin repeat family protein
-2.53	10.62	-1.34	11.29	0.03008118	AT5G59340	WOX2__WUSCHEL related homeobox 2
-2.54	8.39	-1.34	9.06	0.028157	AT1G05065	CLE20__CLAVATA3/ESR-RELATED 20
-2.54	11.85	-1.35	12.52	0.0081946	AT1G07450	NAD(P)-binding Rossmann-fold superfamily protein
-2.54	12.2	-1.34	12.87	0.01036777	AT1G67070	DIN9_PMI2__Mannose-6-phosphate isomerase, type I
-2.54	11.01	-1.34	11.68	0.0151742	AT3G14450	CID9__CTC-interacting domain 9
-2.54	11.83	-1.34	12.5	0.01201455	AT5G09930	ABCF2__ABC transporter family protein
-2.55	12.74	-1.35	13.41	0.03542162	AT1G09240	ATNAS3_NAS3__nicotianamine synthase 3

-2.55	9.46	-1.35	10.14	0.00394474	AT1G09890	Rhamnogalacturonate lyase family protein
-2.55	12.29	-1.35	12.97	0.00430342	AT1G28610	GDSL-like Lipase/Acylhydrolase superfamily protein GDSL-like Lipase/Acylhydrolase superfamily protein
-2.55	10.66	-1.35	11.33	0.00294186	AT1G58602	LRR and NB-ARC domains-containing disease resistance protein
-2.55	8.92	-1.35	9.59	0.0052996	AT3G14700	SART-1 family
-2.55	9.67	-1.35	10.35	0.00554683	AT3G30840	unknown protein; Has 1 Blast hits to 1 proteins in 1 species: Archae - 0; Bacteria - 0; Metazoa - 0; Fungi - 0; Plants - 1; Viruses - 0; Other Eukaryotes - 0 (source: NCBI BLink).
-2.56	10.58	-1.36	11.26	0.01233326	AT3G14760	unknown protein; FUNCTIONS IN: molecular_function unknown; INVOLVED IN: biological_process unknown; LOCATED IN: endomembrane system; EXPRESSED IN: 6 plant structures; EXPRESSED DURING: LP.04 four leaves visible, LP.02 two leaves visible; Has 63 Blast hits to 63 proteins in 13 species: Archae - 0; Bacteria - 0; Metazoa - 0; Fungi - 0; Plants - 63; Viruses - 0; Other Eukaryotes - 0 (source: NCBI BLink).
-2.56	13.71	-1.35	14.39	0.00472051	AT4G31390	Protein kinase superfamily protein
-2.56	14.18	-1.35	14.86	0.00417125	AT5G02180	Transmembrane amino acid transporter family protein
-2.57	11.15	-1.36	11.83	0.01304803	AT1G15690	ATAVP3_AtVHP1;1_AVP-3_AVP1_FUGU5__Inorganic H pyrophosphatase family protein
-2.57	10.96	-1.36	11.65	0.00251625	AT1G75163	
-2.57	12.74	-1.36	13.42	0.0052996	AT3G04140	Ankyrin repeat family protein
-2.57	11.65	-1.36	12.33	0.01013972	AT3G25670	Leucine-rich repeat (LRR) family protein
-2.57	13.19	-1.36	13.87	0.00356968	AT4G23300	CRK22__cysteine-rich RLK (RECEPTOR-like protein kinase) 22
-2.57	13.92	-1.36	14.6	0.00514085	AT5G58770	Undecaprenyl pyrophosphate synthetase family protein
-2.58	12.51	-1.37	13.19	0.00760062	AT1G51470	BGLU35_TGG5__beta glucosidase 35
-2.58	11.82	-1.37	12.5	0.00229082	AT4G34060	DML3__demeter-like protein 3 DML3__demeter-like protein 3
-2.58	13.59	-1.37	14.28	0.01453086	AT5G15950	Adenosylmethionine decarboxylase family protein
-2.58	9.4	-1.37	10.08	0.00510371	AT5G51970	GroES-like zinc-binding alcohol dehydrogenase family protein
-2.59	10.67	-1.37	11.35	0.00420934	AT1G05540	Protein of unknown function (DUF295)
-2.59	8.12	-1.37	8.81	0.00417125	AT3G17185	
-2.6	12.62	-1.38	13.3	0.00472051	AT1G03055	unknown protein; BEST Arabidopsis thaliana protein match is: unknown protein (TAIR:AT1G64680.1); Has 35333 Blast hits to 34131 proteins in 2444 species: Archae - 798; Bacteria - 22429; Metazoa - 974; Fungi - 991; Plants - 531; Viruses - 0; Other Eukaryotes - 9610 (source: NCBI BLink). unknown protein; BEST Arabidopsis thaliana protein match is: unknown protein (TAIR:AT1G64680.1); Has 35333 Blast hits to 34131 proteins in 2444 species: Archae - 798; Bacteria - 22429; Metazoa - 974; Fungi - 991; Plants - 531; Viruses - 0; Other Eukaryotes - 9610 (source: NCBI BLink).
-2.6	12.67	-1.38	13.36	0.00767206	AT1G26820	RNS3__ribonuclease 3
-2.6	12.28	-1.38	12.97	0.00470122	AT1G28610	GDSL-like Lipase/Acylhydrolase superfamily protein GDSL-like Lipase/Acylhydrolase superfamily protein

-2.6	9.17	-1.38	9.86	0.02414463	AT1G55950	DNA-binding storekeeper protein-related transcriptional regulator
-2.6	10.65	-1.38	11.34	0.00442816	AT2G04790	unknown protein; FUNCTIONS IN: molecular_function unknown; INVOLVED IN: biological_process unknown; EXPRESSED IN: 17 plant structures; EXPRESSED DURING: 11 growth stages; Has 33 Blast hits to 33 proteins in 10 species: Archae - 0; Bacteria - 0; Metazoa - 0; Fungi - 0; Plants - 33; Viruses - 0; Other Eukaryotes - 0 (source: NCBI BLink). unknown protein; FUNCTIONS IN: molecular_function unknown; INVOLVED IN: biological_process unknown; EXPRESSED IN: 17 plant structures; EXPRESSED DURING: 11 growth stages; Has 33 Blast hits to 33 proteins in 10 species: Archae - 0; Bacteria - 0; Metazoa - 0; Fungi - 0; Plants - 33; Viruses - 0; Other Eukaryotes - 0 (source: NCBI BLink). unknown protein; FUNCTIONS IN: molecular_function unknown; INVOLVED IN: biological_process unknown; EXPRESSED IN: 17 plant structures; EXPRESSED DURING: 11 growth stages; Has 33 Blast hits to 33 proteins in 10 species: Archae - 0; Bacteria - 0; Metazoa - 0; Fungi - 0; Plants - 33; Viruses - 0; Other Eukaryotes - 0 (source: NCBI BLink). unknown protein; FUNCTIONS IN: molecular_function unknown; INVOLVED IN: biological_process unknown; EXPRESSED IN: 17 plant structures; EXPRESSED DURING: 11 growth stages; Has 33 Blast hits to 33 proteins in 10 species: Archae - 0; Bacteria - 0; Metazoa - 0; Fungi - 0; Plants - 33; Viruses - 0; Other Eukaryotes - 0 (source: NCBI BLink).
-2.6	11.39	-1.38	12.08	0.01681687	AT3G06520	agenet domain-containing protein
-2.6	11.26	-1.38	11.95	0.00518233	AT3G21360	2-oxoglutarate (2OG) and Fe(II)-dependent oxygenase superfamily protein
-2.6	12.65	-1.38	13.34	0.00900027	AT4G01080	TBL26__TRICHOME BIREFRINGENCE-LIKE 26
-2.6	9.02	-1.38	9.71	0.00413041	AT5G51470	Auxin-responsive GH3 family protein
-2.61	10.48	-1.39	11.18	0.00600392	AT1G05900	ATNTH2_NTH2__endonuclease III 2 ATNTH2_NTH2__endonuclease III 2
-2.61	12.65	-1.38	13.34	0.00356968	AT4G11900	S-locus lectin protein kinase family protein
-2.61	13.6	-1.38	14.29	0.01959754	AT4G31870	ATGPX7_GPX7__glutathione peroxidase 7
-2.61	12.51	-1.39	13.2	0.00833848	AT5G15850	BBX2_ATCOL1_COL1__CONSTANS-like 1
-2.61	12.98	-1.39	13.67	0.00918393	AT5G43860	ATCLH2_CLH2__chlorophyllase 2
-2.62	12.24	-1.39	12.93	0.04152948	AT1G09500	NAD(P)-binding Rossmann-fold superfamily protein
-2.62	10.04	-1.39	10.74	0.00410995	AT3G05327	Cyclin family protein
-2.62	9.72	-1.39	10.41	0.02097806	AT3G16360	AHP4__HPT phosphotransmitter 4
-2.62	10.95	-1.39	11.65	0.00229082	AT5G46490	Disease resistance protein (TIR-NBS-LRR class) family Disease resistance protein (TIR-NBS-LRR class) family
-2.62	9.38	-1.39	10.07	0.01287277	AT5G51030	NAD(P)-binding Rossmann-fold superfamily protein
-2.62	11.68	-1.39	12.37	0.0029534	AT5G61350	Protein kinase superfamily protein
-2.63	9.66	-1.4	10.36	0.00610922	AT1G12440	A20/AN1-like zinc finger family protein
-2.63	11.11	-1.4	11.81	0.00508871	AT1G29600	Zinc finger C-x8-C-x5-C-x3-H type family protein
-2.64	10.06	-1.4	10.76	0.00398842	AT1G04550	BDL_IAA12__AUX/IAA transcriptional regulator family protein

-2.64	12.42	-1.4	13.12	0.00674365	AT3G01660	S-adenosyl-L-methionine-dependent methyltransferases superfamily protein
-2.64	8.36	-1.4	9.06	0.02786959	AT3G56891	Heavy metal transport/detoxification superfamily protein
-2.64	11.23	-1.4	11.93	0.00358217	AT4G13830	J20__DNAJ-like 20 J20__DNAJ-like 20
-2.64	11.35	-1.4	12.05	0.00417546	AT5G14350	Pentatricopeptide repeat (PPR) superfamily protein
-2.65	12.06	-1.41	12.76	0.00394474	AT1G52880	ANAC018_ATNAM_NAM_NARS2__NAC (No Apical Meristem) domain transcriptional regulator superfamily protein
-2.65	9.63	-1.41	10.34	0.00879784	AT1G56710	Pectin lyase-like superfamily protein
-2.65	10.51	-1.4	11.22	0.00313253	AT5G02330	Cysteine/Histidine-rich C1 domain family protein
-2.65	11.49	-1.41	12.2	0.00982602	AT5G24140	SQP2__squalene monooxygenase 2
-2.65	13.2	-1.4	13.9	0.00417125	AT5G55570	unknown protein; LOCATED IN: chloroplast; Has 35333 Blast hits to 34131 proteins in 2444 species: Archae - 798; Bacteria - 22429; Metazoa - 974; Fungi - 991; Plants - 531; Viruses - 0; Other Eukaryotes - 9610 (source: NCBI BLink).
-2.66	12	-1.41	12.7	0.00644964	AT2G03710	AGL3_SEP4__K-box region and MADS-box transcription factor family protein AGL3_SEP4__K-box region and MADS-box transcription factor family protein AGL3_SEP4__K-box region and MADS-box transcription factor family protein
-2.66	9.25	-1.41	9.96	0.0046281	AT3G63375	
-2.66	9.58	-1.41	10.28	0.01649762	AT5G35777	
-2.66	12.25	-1.41	12.95	0.00356968	AT5G61270	PIF7__phytochrome-interacting factor7 PIF7__phytochrome-interacting factor7
-2.67	7.07	-1.42	7.78	0.00810235	AT1G66540	Cytochrome P450 superfamily protein Cytochrome P450 superfamily protein
-2.67	11.88	-1.42	12.58	0.00796998	AT3G18485	ILR2__iaa-leucine resistant 2
-2.67	9.78	-1.42	10.49	0.00367141	AT5G02600	NAKR1_NPCC6__Heavy metal transport/detoxification superfamily protein
-2.67	10.87	-1.42	11.58	0.00833976	AT5G02840	LCL1__LHY/CCA1-like 1
-2.67	10.24	-1.41	10.94	0.02010764	AT5G20225	
-2.67	9.82	-1.42	10.52	0.00704462	AT5G46220	Protein of unknown function (DUF616)
-2.67	10.62	-1.41	11.33	0.0047211	AT5G63470	NF-YC4__nuclear factor Y, subunit C4
-2.69	8.94	-1.43	9.65	0.01039189	AT1G69720	HO3__heme oxygenase 3 HO3__heme oxygenase 3
-2.69	10.78	-1.43	11.5	0.00968394	AT1G71490	Tetratricopeptide repeat (TPR)-like superfamily protein
-2.7	11.35	-1.43	12.07	0.00584196	AT1G29460	SAUR-like auxin-responsive protein family
-2.7	12.3	-1.43	13.01	0.00583609	AT3G06510	ATSFR2_SFR2__Glycosyl hydrolase superfamily protein
-2.7	10.51	-1.43	11.23	0.00394474	AT3G08870	Concanavalin A-like lectin protein kinase family protein
-2.7	8.84	-1.43	9.56	0.00666122	AT3G59510	Leucine-rich repeat (LRR) family protein
-2.7	9.27	-1.44	9.98	0.0056423	AT5G45850	Protein of unknown function (DUF688)
-2.71	9.92	-1.44	10.64	0.01222973	AT2G15060	
-2.71	13.26	-1.44	13.98	0.00445289	AT4G12310	CYP706A5__cytochrome P450, family 706, subfamily A, polypeptide 5
-2.71	9.01	-1.44	9.72	0.00479295	AT4G33820	Glycosyl hydrolase superfamily protein
-2.72	12.49	-1.44	13.22	0.00286412	AT1G05310	Pectin lyase-like superfamily protein

-2.72	11.63	-1.44	12.35	0.00429467	AT1G30860	RING/U-box superfamily protein
-2.73	11.03	-1.45	11.75	0.01526214	AT3G54510	Early-responsive to dehydration stress protein (ERD4)
-2.73	8.59	-1.45	9.31	0.00796998	AT5G60290	unknown protein; Has 1807 Blast hits to 1807 proteins in 277 species: Archae - 0; Bacteria - 0; Metazoa - 736; Fungi - 347; Plants - 385; Viruses - 0; Other Eukaryotes - 339 (source: NCBI BLink).
-2.74	9.78	-1.46	10.51	0.00973549	AT1G49130	BBX17_B-box type zinc finger protein with CCT domain
-2.74	11.23	-1.45	11.95	0.01490902	AT1G51440	alpha/beta-Hydrolases superfamily protein
-2.74	13.26	-1.45	13.98	0.00534835	AT1G65800	ARK2_RK2__receptor kinase 2
-2.74	11.73	-1.46	12.46	0.00784132	AT1G69610	Protein of unknown function (DUF1666)
-2.74	14.34	-1.45	15.06	0.00195989	AT3G54830	Transmembrane amino acid transporter family protein
-2.74	11.56	-1.45	12.28	0.00447426	AT4G10600	RING/FYVE/PHD zinc finger superfamily protein
-2.74	10.8	-1.45	11.52	0.00201069	AT4G24430	Rhamnogalacturonate lyase family protein
-2.75	10.02	-1.46	10.75	0.00712636	AT2G30900	TBL43__TRICHOME BIREFRINGENCE-LIKE 43
-2.75	10.53	-1.46	11.26	0.00294186	AT2G32620	ATCSLB02_ATCSLB2_CSLB02_CSLB02__cellulose synthase-like B
-2.75	9.72	-1.46	10.45	0.00643968	AT3G14452	unknown protein; LOCATED IN: endomembrane system; Has 30201 Blast hits to 17322 proteins in 780 species: Archae - 12; Bacteria - 1396; Metazoa - 17338; Fungi - 3422; Plants - 5037; Viruses - 0; Other Eukaryotes - 2996 (source: NCBI BLink).
-2.75	9.37	-1.46	10.1	0.00640693	AT3G28770	Protein of unknown function (DUF1216)
-2.76	8.85	-1.47	9.58	0.00643035	AT2G14095	unknown protein; FUNCTIONS IN: molecular_function unknown; INVOLVED IN: biological_process unknown; LOCATED IN: mitochondrion; EXPRESSED IN: 7 plant structures; EXPRESSED DURING: 4 anthesis, C globular stage, petal differentiation and expansion stage; Has 106 Blast hits to 103 proteins in 21 species: Archae - 0; Bacteria - 5; Metazoa - 0; Fungi - 4; Plants - 87; Viruses - 0; Other Eukaryotes - 10 (source: NCBI BLink).
-2.76	7.54	-1.46	8.27	0.00392545	AT3G51760	Protein of unknown function (DUF688) Protein of unknown function (DUF688)
-2.76	9.84	-1.47	10.58	0.00433482	AT5G44930	ARAD2__Exostosin family protein
-2.77	7.81	-1.47	8.55	0.00356968	AT2G04240	XERICO__RING/U-box superfamily protein
-2.77	8.66	-1.47	9.39	0.00434991	AT2G30695	FUNCTIONS IN: molecular_function unknown; INVOLVED IN: protein folding, protein transport; LOCATED IN: chloroplast; EXPRESSED IN: 23 plant structures; EXPRESSED DURING: 13 growth stages; CONTAINS InterPro DOMAIN/s: Trigger factor, ribosome-binding, bacterial (InterPro:IPR008881); Has 35333 Blast hits to 34131 proteins in 2444 species: Archae - 798; Bacteria - 22429; Metazoa - 974; Fungi - 991; Plants - 531; Viruses - 0; Other Eukaryotes - 9610 (source: NCBI BLink).
-2.77	13.83	-1.47	14.56	0.00162136	AT4G23290	CRK21__cysteine-rich RLK (RECEPTOR-like protein kinase) 21
-2.78	14.09	-1.48	14.83	0.00758398	AT1G18810	phytochrome kinase substrate-related
-2.78	13.56	-1.48	14.29	0.00219513	AT3G59778	
-2.8	10.12	-1.49	10.87	0.00508871	AT3G19230	Leucine-rich repeat (LRR) family protein

-2.8	9.17	-1.48	9.92	0.00472792	AT4G34650	SQS2__squalene synthase 2
-2.81	10.08	-1.49	10.83	0.01121422	AT4G14548	
-2.82	9.31	-1.49	10.05	0.00695748	AT1G51270	structural molecules;transmembrane receptors;structural molecules
-2.83	14.27	-1.5	15.02	0.00539566	AT3G24460	Serinc-domain containing serine and sphingolipid biosynthesis protein
-2.84	11.39	-1.51	12.15	0.003464	AT2G29370	NAD(P)-binding Rossmann-fold superfamily protein
-2.84	8.89	-1.51	9.64	0.00229082	AT3G10590	Duplicated homeodomain-like superfamily protein
-2.84	9.02	-1.51	9.77	0.00388122	AT3G14420	Aldolase-type TIM barrel family protein Aldolase-type TIM barrel family protein
-2.84	11.01	-1.5	11.76	0.03509508	AT3G22640	PAP85__cupin family protein
-2.84	11.2	-1.51	11.96	0.02399778	AT4G27360	Dynein light chain type 1 family protein
-2.85	11.08	-1.51	11.83	0.00539566	AT1G62240	unknown protein; FUNCTIONS IN: molecular_function unknown; INVOLVED IN: biological_process unknown; LOCATED IN: endomembrane system; Has 186248 Blast hits to 53970 proteins in 2688 species: Archae - 469; Bacteria - 57770; Metazoa - 55630; Fungi - 12355; Plants - 17666; Viruses - 2624; Other Eukaryotes - 39734 (source: NCBI BLink).
-2.85	12.94	-1.51	13.69	0.00581265	AT1G66540	Cytochrome P450 superfamily protein Cytochrome P450 superfamily protein
-2.85	12.86	-1.51	13.62	0.00267504	AT5G48920	TED7__tracheary element differentiation-related 7
-2.85	11.03	-1.51	11.79	0.00788305	AT5G58140	NPL1_PHOT2__phototropin 2
-2.85	9.17	-1.51	9.92	0.00784132	AT5G62100	ATBAG2_BAG2__BCL-2-associated athanogene 2
-2.86	11.08	-1.51	11.83	0.00294186	AT1G50280	Phototropic-responsive NPH3 family protein
-2.86	11.53	-1.52	12.29	0.00605413	AT3G10150	ATPAP16_PAP16__purple acid phosphatase 16
-2.86	14.13	-1.51	14.89	0.00507786	AT3G48310	CYP71A22__cytochrome P450, family 71, subfamily A, polypeptide 22
-2.87	11.57	-1.52	12.33	0.00810235	AT1G16090	WAKL7__wall associated kinase-like 7
-2.87	9.06	-1.52	9.82	0.00683646	AT1G65570	Pectin lyase-like superfamily protein
-2.87	8.93	-1.52	9.69	0.01150797	AT1G69720	HO3__heme oxygenase 3 HO3__heme oxygenase 3
-2.87	9.98	-1.52	10.74	0.00326511	AT1G76955	Expressed protein
-2.87	12.82	-1.52	13.58	0.02530678	AT2G21330	FBA1__fructose-bisphosphate aldolase 1
-2.87	12.99	-1.52	13.75	0.00306866	AT2G29120	ATGLR2.7_GLR2.7_GLR2.7__glutamate receptor 2.7
-2.87	9.47	-1.52	10.23	0.00294186	AT4G34320	Protein of unknown function (DUF677)
-2.87	8.84	-1.52	9.6	0.00601931	AT5G56700	FBD / Leucine Rich Repeat domains containing protein
-2.88	12.01	-1.53	12.77	0.00764879	AT2G03710	AGL3_SEP4__K-box region and MADS-box transcription factor family protein AGL3_SEP4__K-box region and MADS-box transcription factor family protein AGL3_SEP4__K-box region and MADS-box transcription factor family protein
-2.88	12.71	-1.53	13.47	0.00784613	AT2G27395	
-2.88	8.98	-1.53	9.74	0.00195989	AT3G26125	CYP86C2__cytochrome P450, family 86, subfamily C, polypeptide 2
-2.88	12.81	-1.53	13.57	0.01459363	AT5G42760	Leucine carboxyl methyltransferase
-2.89	11.17	-1.53	11.94	0.00417125	AT2G04790	unknown protein; FUNCTIONS IN: molecular_function unknown; INVOLVED IN: biological_process unknown; EXPRESSED IN: 17 plant structures; EXPRESSED DURING: 11

						growth stages; Has 33 Blast hits to 33 proteins in 10 species: Archae - 0; Bacteria - 0; Metazoa - 0; Fungi - 0; Plants - 33; Viruses - 0; Other Eukaryotes - 0 (source: NCBI BLink). unknown protein; FUNCTIONS IN: molecular_function unknown; INVOLVED IN: biological_process unknown; EXPRESSED IN: 17 plant structures; EXPRESSED DURING: 11 growth stages; Has 33 Blast hits to 33 proteins in 10 species: Archae - 0; Bacteria - 0; Metazoa - 0; Fungi - 0; Plants - 33; Viruses - 0; Other Eukaryotes - 0 (source: NCBI BLink). unknown protein; FUNCTIONS IN: molecular_function unknown; INVOLVED IN: biological_process unknown; EXPRESSED IN: 17 plant structures; EXPRESSED DURING: 11 growth stages; Has 33 Blast hits to 33 proteins in 10 species: Archae - 0; Bacteria - 0; Metazoa - 0; Fungi - 0; Plants - 33; Viruses - 0; Other Eukaryotes - 0 (source: NCBI BLink). unknown protein; FUNCTIONS IN: molecular_function unknown; INVOLVED IN: biological_process unknown; EXPRESSED IN: 17 plant structures; EXPRESSED DURING: 11 growth stages; Has 33 Blast hits to 33 proteins in 10 species: Archae - 0; Bacteria - 0; Metazoa - 0; Fungi - 0; Plants - 33; Viruses - 0; Other Eukaryotes - 0 (source: NCBI BLink).
-2.89	11.1	-1.53	11.86	0.00583609	AT2G45800	PLIM2a__GATA type zinc finger transcription factor family protein PLIM2a__GATA type zinc finger transcription factor family protein
-2.89	8.07	-1.53	8.83	0.00492377	AT5G61910	DCD (Development and Cell Death) domain protein
-2.9	9.61	-1.54	10.38	0.0030017	AT1G01240	unknown protein; BEST Arabidopsis thaliana protein match is: unknown protein (TAIR:AT2G46550.1); Has 35333 Blast hits to 34131 proteins in 2444 species: Archae - 798; Bacteria - 22429; Metazoa - 974; Fungi - 991; Plants - 531; Viruses - 0; Other Eukaryotes - 9610 (source: NCBI BLink).
-2.9	11.29	-1.54	12.06	0.00580211	AT1G17990	FMN-linked oxidoreductases superfamily protein
-2.91	9.83	-1.54	10.61	0.01013972	AT2G34440	AGL29__AGAMOUS-like 29
-2.92	13.24	-1.54	14.02	0.00179583	AT3G48280	CYP71A25__cytochrome P450, family 71, subfamily A, polypeptide 25
-2.92	10.38	-1.55	11.16	0.003464	AT5G59845	Gibberellin-regulated family protein
-2.93	10.02	-1.55	10.79	0.04012296	AT1G10586	basic helix-loop-helix (bHLH) DNA-binding superfamily protein basic helix-loop-helix (bHLH) DNA-binding superfamily protein
-2.93	10.02	-1.55	10.79	0.01304971	AT4G04490	CRK36__cysteine-rich RLK (RECEPTOR-like protein kinase) 36
-2.94	9.04	-1.55	9.82	0.00356968	AT1G10657	Plant protein 1589 of unknown function
-2.94	9.56	-1.55	10.34	0.00434282	AT1G76954	Defensin-like (DEFL) family protein
-2.94	9.12	-1.55	9.9	0.00735554	AT3G48070	RING/U-box superfamily protein
-2.94	12.14	-1.56	12.92	0.00673815	AT4G27820	BGLU9__beta glucosidase 9
-2.95	9.42	-1.56	10.2	0.00421466	AT3G29185	Domain of unknown function (DUF3598)
-2.95	9.48	-1.56	10.26	0.0029534	AT3G57460	catalytics;metal ion binding
-2.95	10.52	-1.56	11.3	0.01075468	AT4G23150	CRK7__cysteine-rich RLK (RECEPTOR-like protein kinase) 7
-2.95	8.19	-1.56	8.97	0.01191571	AT5G13360	Auxin-responsive GH3 family protein

-2.95	7.22	-1.56	8	0.00479295	AT5G56800	Protein with RNI-like/FBD-like domains
-2.96	12.58	-1.57	13.37	0.00543099	AT2G29090	CYP707A2__cytochrome P450, family 707, subfamily A, polypeptide 2
-2.96	11.56	-1.57	12.34	0.00809014	AT5G05420	FKBP-like peptidyl-prolyl cis-trans isomerase family protein
-2.97	9.02	-1.57	9.8	0.00702592	AT1G53160	SPL4__squamosa promoter binding protein-like 4 SPL4__squamosa promoter binding protein-like 4
-2.98	12.18	-1.57	12.96	0.00288203	AT1G31173	
-2.98	9.25	-1.57	10.04	0.02889784	AT4G04510	CRK38__cysteine-rich RLK (RECEPTOR-like protein kinase) 38
-2.99	13.2	-1.58	13.99	0.0100502	AT1G58520	RXW8__lipases;hydrolases, acting on ester bonds
-2.99	10.05	-1.58	10.84	0.010454	AT4G19820	Glycosyl hydrolase family protein with chitinase insertion domain
-3.01	12.77	-1.59	13.56	0.00394474	AT1G76530	Auxin efflux carrier family protein
-3.01	12.27	-1.59	13.07	0.00309366	AT3G20270	lipid-binding serum glycoprotein family protein
-3.03	11.39	-1.6	12.19	0.00306866	AT4G18425	Protein of unknown function (DUF679)
-3.04	8.63	-1.61	9.44	0.00229082	AT2G03530	ATUPS2_UPS2__ureide permease 2 ATUPS2_UPS2__ureide permease 2
-3.04	10.3	-1.6	11.1	0.00542262	AT2G06002	
-3.04	8.51	-1.6	9.32	0.00962279	AT3G61230	PLIM2c__GATA type zinc finger transcription factor family protein
-3.04	10.11	-1.6	10.91	0.01500365	AT4G04500	CRK37__cysteine-rich RLK (RECEPTOR-like protein kinase) 37
-3.04	8.28	-1.6	9.08	0.03649194	AT5G05270	Chalcone-flavanone isomerase family protein
-3.05	13.1	-1.61	13.91	0.0242498	AT1G33960	AIG1__P-loop containing nucleoside triphosphate hydrolases superfamily protein
-3.05	11.46	-1.61	12.27	0.00420969	AT3G28140	RNA ligase/cyclic nucleotide phosphodiesterase family protein RNA ligase/cyclic nucleotide phosphodiesterase family protein
-3.05	13.03	-1.61	13.84	0.00608255	AT4G13810	AtRLP47_RLP47__receptor like protein 47
-3.06	9.39	-1.61	10.2	0.00999028	AT1G79529	
-3.06	8.93	-1.61	9.74	0.00430342	AT3G46375	
-3.07	7.93	-1.62	8.74	0.003464	AT1G48610	AT hook motif-containing protein
-3.07	9.51	-1.62	10.32	0.00546655	AT2G22200	Integrase-type DNA-binding superfamily protein
-3.07	8.58	-1.62	9.39	0.00356968	AT4G15710	unknown protein; Has 18 Blast hits to 18 proteins in 2 species: Archae - 0; Bacteria - 0; Metazoa - 0; Fungi - 0; Plants - 18; Viruses - 0; Other Eukaryotes - 0 (source: NCBI BLink).
-3.08	10.61	-1.63	11.42	0.00445289	AT1G26208	
-3.08	10.52	-1.62	11.33	0.01967281	AT5G49920	Octicosapeptide/Phox/Bem1p family protein
-3.08	10.86	-1.62	11.67	0.01867869	AT5G60900	RLK1__receptor-like protein kinase 1
-3.09	11.7	-1.63	12.52	0.00367141	AT1G01390	UDP-Glycosyltransferase superfamily protein
-3.11	7.41	-1.63	8.23	0.00779272	AT3G26815	
-3.11	10.28	-1.64	11.1	0.00826348	AT5G15800	AGL2_SEP1__K-box region and MADS-box transcription factor family protein AGL2_SEP1__K-box region and MADS-box transcription factor family protein
-3.12	9.47	-1.64	10.29	0.01426607	AT2G25780	Protein of unknown function (DUF1677)
-3.13	8.64	-1.65	9.46	0.00944571	AT1G04880	HMG (high mobility group) box protein with ARID/BRIGHT DNA-binding domain

-3.13	9.13	-1.65	9.95	0.00253321	AT1G32540	LOL1__lsd one like 1
-3.14	9.41	-1.65	10.23	0.01304559	AT3G48240	Octicosapeptide/Phox/Bem1p family protein
-3.15	9.22	-1.66	10.05	0.00323071	AT2G39240	RNA polymerase I specific transcription initiation factor RRN3 protein
-3.16	9.5	-1.66	10.33	0.01023906	AT1G66060	Family of unknown function (DUF577)
-3.17	8.81	-1.66	9.64	0.00487427	AT1G07010	AtSLP1_SLP1__Calcineurin-like metallo-phosphoesterase superfamily protein
-3.17	12.95	-1.67	13.79	0.00286412	AT4G36530	alpha/beta-Hydrolases superfamily protein
-3.19	10.07	-1.67	10.9	0.01226263	AT3G15840	PIFI__post-illumination chlorophyll fluorescence increase
-3.2	9.76	-1.68	10.6	0.00294186	AT1G04640	LIP2__lipoyltransferase 2
-3.2	10.34	-1.68	11.18	0.02236798	AT1G64710	GroES-like zinc-binding dehydrogenase family protein
-3.2	11.71	-1.68	12.55	0.01427376	AT5G16960	Zinc-binding dehydrogenase family protein
-3.21	9.78	-1.68	10.62	0.01008356	AT1G19640	JMT__jasmonic acid carboxyl methyltransferase
-3.21	8.42	-1.68	9.26	0.00947649	AT1G25430	
-3.21	7.71	-1.68	8.55	0.00309366	AT2G27030	ACAM-2_CAM5__calmodulin 5
-3.22	11	-1.69	11.84	0.003464	AT1G58889	
-3.23	11.1	-1.69	11.94	0.00315341	AT2G29290	NAD(P)-binding Rossmann-fold superfamily protein
-3.23	9.44	-1.69	10.29	0.02074873	AT3G44350	anac061_NAC061__NAC domain containing protein 61
-3.24	11.29	-1.7	12.14	0.00451369	AT5G28910	unknown protein; FUNCTIONS IN: molecular_function unknown; INVOLVED IN: biological_process unknown; BEST Arabidopsis thaliana protein match is: unknown protein (TAIR:AT5G28960.1); Has 82 Blast hits to 80 proteins in 14 species: Archae - 0; Bacteria - 0; Metazoa - 1; Fungi - 0; Plants - 78; Viruses - 0; Other Eukaryotes - 3 (source: NCBI BLink). unknown protein; FUNCTIONS IN: molecular_function unknown; INVOLVED IN: biological_process unknown; BEST Arabidopsis thaliana protein match is: unknown protein (TAIR:AT5G28960.1); Has 82 Blast hits to 80 proteins in 14 species: Archae - 0; Bacteria - 0; Metazoa - 1; Fungi - 0; Plants - 78; Viruses - 0; Other Eukaryotes - 3 (source: NCBI BLink).
-3.25	11.72	-1.7	12.57	0.00413041	AT1G69523	S-adenosyl-L-methionine-dependent methyltransferases superfamily protein
-3.26	11.05	-1.7	11.9	0.00562376	AT1G53870	Protein of unknown function (DUF567) Protein of unknown function (DUF567)
-3.27	10.18	-1.71	11.03	0.01453255	AT3G19270	CYP707A4__cytochrome P450, family 707, subfamily A, polypeptide 4
-3.28	9.5	-1.71	10.36	0.00610922	AT2G29280	
-3.28	9.62	-1.71	10.48	0.00326511	AT3G01960	unknown protein; Has 13 Blast hits to 13 proteins in 5 species: Archae - 0; Bacteria - 0; Metazoa - 0; Fungi - 0; Plants - 13; Viruses - 0; Other Eukaryotes - 0 (source: NCBI BLink).
-3.28	10.3	-1.71	11.16	0.00947254	AT3G23120	AtRLP38_RLP38__receptor like protein 38
-3.28	10.49	-1.72	11.35	0.00269259	AT4G09760	Protein kinase superfamily protein
-3.28	8.48	-1.71	9.34	0.003464	AT5G53380	O-acyltransferase (WSD1-like) family protein
-3.29	13.29	-1.72	14.15	0.00507786	AT3G28540	P-loop containing nucleoside triphosphate hydrolases superfamily protein
-3.33	8.16	-1.74	9.03	0.00517823	AT3G51760	Protein of unknown function (DUF688) Protein of unknown function (DUF688)
-3.35	7.69	-1.75	8.56	0.01121422	AT1G72980	LBD7__LOB domain-containing protein 7

-3.35	9.91	-1.75	10.79	0.00329522	AT3G61430	ATPIP1_PIP1_PIP1;1_PIP1A__plasma membrane intrinsic protein 1A
-3.37	11.84	-1.75	12.72	0.00356968	AT2G03710	AGL3_SEP4__K-box region and MADS-box transcription factor family protein AGL3_SEP4__K-box region and MADS-box transcription factor family protein AGL3_SEP4__K-box region and MADS-box transcription factor family protein
-3.37	10.89	-1.75	11.76	0.01027468	AT3G21890	BBX31_B-box type zinc finger family protein
-3.37	9.58	-1.75	10.46	0.00742041	AT3G26040	HXXXD-type acyl-transferase family protein
-3.37	9.43	-1.75	10.31	0.00784768	AT5G18407	Defensin-like (DEFL) family protein
-3.38	10.94	-1.76	11.82	0.04871459	AT4G15248	BBX30_B-box type zinc finger family protein
-3.39	10.71	-1.76	11.59	0.00896923	AT1G03055	unknown protein; BEST Arabidopsis thaliana protein match is: unknown protein (TAIR:AT1G64680.1); Has 35333 Blast hits to 34131 proteins in 2444 species: Archae - 798; Bacteria - 22429; Metazoa - 974; Fungi - 991; Plants - 531; Viruses - 0; Other Eukaryotes - 9610 (source: NCBI BLink). unknown protein; BEST Arabidopsis thaliana protein match is: unknown protein (TAIR:AT1G64680.1); Has 35333 Blast hits to 34131 proteins in 2444 species: Archae - 798; Bacteria - 22429; Metazoa - 974; Fungi - 991; Plants - 531; Viruses - 0; Other Eukaryotes - 9610 (source: NCBI BLink).
-3.39	10.59	-1.76	11.47	0.03871652	AT1G44575	CP22_NPQ4_PSBS__Chlorophyll A-B binding family protein
-3.39	13.73	-1.76	14.61	0.00358222	AT4G14400	ACD6__ankyrin repeat family protein
-3.4	12.79	-1.76	13.67	0.00221781	AT1G69730	Wall-associated kinase family protein
-3.41	10.47	-1.77	11.36	0.02152208	AT1G18710	AtMYB47_MYB47__myb domain protein 47
-3.41	11.35	-1.77	12.23	0.00294186	AT3G59580	Plant regulator RWP-RK family protein
-3.41	8.21	-1.77	9.09	0.00472051	AT5G46871	Putative membrane lipoprotein
-3.42	8.98	-1.78	9.87	0.00392318	AT3G13403	Defensin-like (DEFL) family protein
-3.44	11.29	-1.78	12.18	0.00404197	AT5G28910	unknown protein; FUNCTIONS IN: molecular_function unknown; INVOLVED IN: biological_process unknown; BEST Arabidopsis thaliana protein match is: unknown protein (TAIR:AT5G28960.1); Has 82 Blast hits to 80 proteins in 14 species: Archae - 0; Bacteria - 0; Metazoa - 1; Fungi - 0; Plants - 78; Viruses - 0; Other Eukaryotes - 3 (source: NCBI BLink). unknown protein; FUNCTIONS IN: molecular_function unknown; INVOLVED IN: biological_process unknown; BEST Arabidopsis thaliana protein match is: unknown protein (TAIR:AT5G28960.1); Has 82 Blast hits to 80 proteins in 14 species: Archae - 0; Bacteria - 0; Metazoa - 1; Fungi - 0; Plants - 78; Viruses - 0; Other Eukaryotes - 3 (source: NCBI BLink).
-3.45	8.77	-1.79	9.66	0.0039921	AT2G14060	S-adenosyl-L-methionine-dependent methyltransferases superfamily protein
-3.45	10.19	-1.78	11.08	0.01075069	AT3G52310	ABCG27__ABC-2 type transporter family protein
-3.46	12.03	-1.79	12.92	0.00835941	AT2G32680	AtRLP23_RLP23__receptor like protein 23
-3.46	7.71	-1.79	8.61	0.00784132	AT5G15853	unknown protein; LOCATED IN: endomembrane system; Has 30201 Blast hits to 17322 proteins in 780 species: Archae - 12; Bacteria - 1396; Metazoa - 17338; Fungi - 3422; Plants - 5037; Viruses - 0; Other Eukaryotes - 2996 (source: NCBI BLink).

-3.47	10.27	-1.79	11.17	0.0075122	AT3G23110	AtRLP37_EMB2800_RLP37__receptor like protein 37
-3.48	8.45	-1.8	9.35	0.03715538	AT2G45560	CYP76C1__cytochrome P450, family 76, subfamily C, polypeptide 1
-3.48	10.92	-1.8	11.82	0.0055488	AT3G01480	ATCYP38_CYP38__cyclophilin 38
-3.49	11.05	-1.8	11.95	0.01819975	AT1G09080	BIP3__Heat shock protein 70 (Hsp 70) family protein
-3.5	10.43	-1.81	11.34	0.00294186	AT3G01100	ATHYP1_HYP1__hypothetical protein 1
-3.5	11.27	-1.81	12.17	0.00592108	AT3G59250	F-box/RNI-like superfamily protein
-3.5	8.03	-1.81	8.93	0.01377727	AT4G32630	ArfGap/RecO-like zinc finger domain-containing protein
-3.5	9.21	-1.81	10.12	0.03578152	AT5G59670	Leucine-rich repeat protein kinase family protein
-3.51	7.78	-1.81	8.68	0.03063386	AT1G48020	ATPMEI1_PMEI1__pectin methylesterase inhibitor 1
-3.51	10.45	-1.81	11.36	0.00381387	AT2G40020	Nucleolar histone methyltransferase-related protein
-3.52	10.14	-1.82	11.05	0.00978611	AT3G21460	Glutaredoxin family protein
-3.53	8.96	-1.82	9.87	0.00681626	AT1G07330	unknown protein; BEST Arabidopsis thaliana protein match is: unknown protein (TAIR:AT2G29620.1); Has 597 Blast hits to 536 proteins in 121 species: Archae - 2; Bacteria - 47; Metazoa - 170; Fungi - 43; Plants - 98; Viruses - 0; Other Eukaryotes - 237 (source: NCBI BLink).
-3.54	9.27	-1.83	10.19	0.00411275	AT1G75250	ATRL6_RL6_RSM3__RAD-like 6
-3.55	10.9	-1.83	11.82	0.0040103	AT4G13260	YUC2__Flavin-binding monooxygenase family protein
-3.57	11.26	-1.84	12.18	0.00326047	AT5G56850	unknown protein; EXPRESSED IN: 21 plant structures; EXPRESSED DURING: 13 growth stages; Has 35333 Blast hits to 34131 proteins in 2444 species: Archae - 798; Bacteria - 22429; Metazoa - 974; Fungi - 991; Plants - 531; Viruses - 0; Other Eukaryotes - 9610 (source: NCBI BLink). unknown protein; EXPRESSED IN: 21 plant structures; EXPRESSED DURING: 13 growth stages; Has 35333 Blast hits to 34131 proteins in 2444 species: Archae - 798; Bacteria - 22429; Metazoa - 974; Fungi - 991; Plants - 531; Viruses - 0; Other Eukaryotes - 9610 (source: NCBI BLink).
-3.6	8.08	-1.85	9	0.00329522	AT5G26618	
-3.61	10.61	-1.85	11.54	0.03203546	AT1G69140	
-3.61	11.08	-1.85	12.01	0.00627604	AT4G26530	Aldolase superfamily protein
-3.61	13.15	-1.85	14.07	0.00737142	AT5G38510	Rhomboid-related intramembrane serine protease family protein Rhomboid-related intramembrane serine protease family protein
-3.62	10.5	-1.86	11.43	0.00592108	AT4G38960	BBX19_B-box type zinc finger family protein B-box type zinc finger family protein
-3.64	10.44	-1.86	11.37	0.02093813	AT1G18730	NDF6_PnsB4__NDH dependent flow 6
-3.65	8.12	-1.87	9.06	0.00294186	AT5G16023	DVL1_RTFL18__ROTUNDIFOLIA like 18
-3.66	11.59	-1.87	12.53	0.00371237	AT3G09160	RNA-binding (RRM/RBD/RNP motifs) family protein
-3.66	8.52	-1.87	9.45	0.0434544	AT3G15536	
-3.67	9.38	-1.88	10.32	0.01073689	AT4G34240	ALDH3_ALDH3I1__aldehyde dehydrogenase 3I1
-3.68	10.7	-1.88	11.64	0.00500089	AT1G13130	Cellulase (glycosyl hydrolase family 5) protein

-3.68	12.62	-1.88	13.56	0.0029534	AT1G32780	GroES-like zinc-binding dehydrogenase family protein
-3.69	7.69	-1.89	8.63	0.00603002	AT1G26140	unknown protein; BEST Arabidopsis thaliana protein match is: unknown protein (TAIR:AT1G68700.1); Has 35 Blast hits to 35 proteins in 8 species: Archae - 0; Bacteria - 0; Metazoa - 0; Fungi - 0; Plants - 35; Viruses - 0; Other Eukaryotes - 0 (source: NCBI BLink).
-3.69	8.82	-1.88	9.76	0.00279459	AT1G64770	NDF2_NDH45_PnsB2__NDH-dependent cyclic electron flow 1
-3.7	9.62	-1.89	10.56	0.00309366	AT1G22590	AGL87__AGAMOUS-like 87 AGL87__AGAMOUS-like 87
-3.7	11.43	-1.89	12.38	0.01528848	AT5G66740	Protein of unknown function (DUF620)
-3.71	9.35	-1.89	10.29	0.00965735	AT1G76190	SAUR-like auxin-responsive protein family
-3.72	7.25	-1.9	8.2	0.0048338	AT5G41780	myosin heavy chain-related
-3.77	8.56	-1.91	9.51	0.00860028	AT1G78290	SNRK2-8_SNRK2.8_SRK2C__Protein kinase superfamily protein
-3.78	9.94	-1.92	10.9	0.00309366	AT1G65790	ARK1_RK1__receptor kinase 1
-3.79	12.89	-1.92	13.86	0.00426996	AT5G59130	Subtilase family protein
-3.84	11.58	-1.94	12.55	0.00605413	AT1G43675	
-3.85	9.38	-1.94	10.35	0.04906014	AT1G01310	CAP (Cysteine-rich secretory proteins, Antigen 5, and Pathogenesis-related 1 protein) superfamily protein
-3.85	8.22	-1.94	9.19	0.04063691	AT1G13607	Defensin-like (DEFL) family protein
-3.86	13.37	-1.95	14.35	0.00659732	AT3G14420	Aldolase-type TIM barrel family protein Aldolase-type TIM barrel family protein
-3.89	11.25	-1.96	12.23	0.0284224	AT4G17098	
-3.94	12.99	-1.98	13.98	0.00367141	AT5G38510	Rhomboid-related intramembrane serine protease family protein Rhomboid-related intramembrane serine protease family protein
-3.95	10.38	-1.98	11.37	0.00572337	AT5G28020	ATCYSD2_CYSD2__cysteine synthase D2
-3.97	11.3	-1.99	12.29	0.00417125	AT3G13065	SRF4__STRUBBELIG-receptor family 4
-3.97	10.42	-1.99	11.42	0.01440026	AT3G15310	
-3.99	10.23	-2	11.23	0.01165814	AT2G14560	LURP1__Protein of unknown function (DUF567)
-4	12.16	-2	13.16	0.01040772	AT5G54610	ANK__ankyrin
-4.01	11.46	-2	12.46	0.021903	AT1G01520	ASG4__Homeodomain-like superfamily protein
-4.04	9.6	-2.02	10.61	0.00411275	AT3G55580	Regulator of chromosome condensation (RCC1) family protein
-4.11	11.1	-2.04	12.12	0.00514085	AT3G45860	CRK4__cysteine-rich RLK (RECEPTOR-like protein kinase) 4
-4.15	10.2	-2.05	11.23	0.00250146	AT1G45616	AtRLP6_RLP6__receptor like protein 6
-4.16	11.31	-2.06	12.34	0.02979336	AT4G35090	CAT2__catalase 2
-4.18	12.64	-2.06	13.67	0.00229082	AT3G46370	Leucine-rich repeat protein kinase family protein
-4.19	9.25	-2.07	10.29	0.00326047	AT3G27970	Exonuclease family protein
-4.22	8.42	-2.08	9.46	0.00404197	AT2G25095	
-4.26	7.69	-2.09	8.74	0.00196725	AT3G57810	Cysteine proteinases superfamily protein
-4.27	9.08	-2.09	10.12	0.005834	AT2G04039	unknown protein; FUNCTIONS IN: molecular_function unknown; LOCATED IN: chloroplast; EXPRESSED IN: 21 plant structures; EXPRESSED DURING: 13 growth stages; CONTAINS

						InterPro DOMAIN/s: Protein of unknown function DUF2996 (InterPro:IPR021374); Has 38 Blast hits to 38 proteins in 13 species: Archae - 0; Bacteria - 0; Metazoa - 0; Fungi - 0; Plants - 38; Viruses - 0; Other Eukaryotes - 0 (source: NCBI BLink).
-4.32	10.41	-2.11	11.46	0.00663139	AT5G65890	ACR1__ACT domain repeat 1 ACR1__ACT domain repeat 1
-4.34	10.61	-2.12	11.67	0.00294186	AT3G48298	unknown protein; Has 30201 Blast hits to 17322 proteins in 780 species: Archae - 12; Bacteria - 1396; Metazoa - 17338; Fungi - 3422; Plants - 5037; Viruses - 0; Other Eukaryotes - 2996 (source: NCBI BLink).
-4.34	9.3	-2.12	10.35	0.0034714	AT4G13830	J20__DNAJ-like 20 J20__DNAJ-like 20
-4.38	8.43	-2.13	9.5	0.04799851	AT1G07050	CCT motif family protein
-4.42	10.32	-2.15	11.39	0.00610922	AT2G36760	UGT73C2__UDP-glucosyl transferase 73C2
-4.45	9.97	-2.15	11.05	0.00566288	AT1G77960	unknown protein; BEST Arabidopsis thaliana protein match is: unknown protein (TAIR:AT1G28630.4); Has 13187 Blast hits to 6837 proteins in 407 species: Archae - 0; Bacteria - 447; Metazoa - 4509; Fungi - 1333; Plants - 399; Viruses - 45; Other Eukaryotes - 6454 (source: NCBI BLink).
-4.46	11.98	-2.16	13.06	0.00356968	AT5G10250	DOT3__Phototropic-responsive NPH3 family protein
-4.5	9.98	-2.17	11.06	0.02467735	AT1G24580	RING/U-box superfamily protein
-4.54	9.24	-2.18	10.33	0.00323071	AT1G52510	alpha/beta-Hydrolases superfamily protein
-4.54	8.48	-2.18	9.57	0.00449194	AT3G28070	nodulin MtN21 /EamA-like transporter family protein
-4.58	10.93	-2.2	12.03	0.00926782	AT1G19510	ATRL5_RL5_RSM4__RAD-like 5
-4.64	10.59	-2.21	11.7	0.00050433	AT4G33810	Glycosyl hydrolase superfamily protein
-4.71	9.69	-2.24	10.81	0.00294186	AT5G05430	RNA-binding protein
-4.72	8.99	-2.24	10.11	0.00294186	AT3G61898	unknown protein; FUNCTIONS IN: molecular_function unknown; INVOLVED IN: biological_process unknown; LOCATED IN: mitochondrion; Has 35333 Blast hits to 34131 proteins in 2444 species: Archae - 798; Bacteria - 22429; Metazoa - 974; Fungi - 991; Plants - 531; Viruses - 0; Other Eukaryotes - 9610 (source: NCBI BLink).
-4.73	12.08	-2.24	13.2	0.00455724	AT1G66840	PMI2_WEB2__Plant protein of unknown function (DUF827)
-4.82	8.97	-2.27	10.1	0.00309366	AT2G29165	
-4.85	9.8	-2.28	10.94	0.00102599	AT1G29724	
-5.04	12.21	-2.33	13.37	0.01255441	AT2G36750	UGT73C1__UDP-glucosyl transferase 73C1
-5.05	9.98	-2.34	11.15	0.0039427	AT5G15800	AGL2_SEP1__K-box region and MADS-box transcription factor family protein AGL2_SEP1__K-box region and MADS-box transcription factor family protein
-5.13	10.57	-2.36	11.75	0.00844279	AT1G20120	GDSL-like Lipase/Acylhydrolase superfamily protein
-5.13	11.83	-2.36	13.01	0.00877158	AT4G34550	BEST Arabidopsis thaliana protein match is: F-box family protein (TAIR:AT2G16365.3); Has 37 Blast hits to 37 proteins in 8 species: Archae - 0; Bacteria - 0; Metazoa - 0; Fungi - 0; Plants - 37; Viruses - 0; Other Eukaryotes - 0 (source: NCBI BLink).
-5.22	9.82	-2.38	11.01	0.00487529	AT4G03450	Ankyrin repeat family protein

-5.23	8.55	-2.39	9.75	0.03606894	AT5G44430	PDF1.2c__plant defensin 1.2C
-5.29	8.9	-2.4	10.1	0.00294186	AT1G22370	AtUGT85A5_UGT85A5__UDP-glucosyl transferase 85A5
-5.46	12.44	-2.45	13.66	0.0052996	AT2G27420	Cysteine proteinases superfamily protein
-5.53	10.78	-2.47	12.01	0.00162136	AT3G13062	Polyketide cyclase/dehydrase and lipid transport superfamily protein
-5.8	11.71	-2.54	12.98	0.00346132	AT2G32290	BAM6_BMY5__beta-amylase 6
-5.84	10.57	-2.55	11.85	0.0029534	AT1G42560	ATMLO9_MLO9__Seven transmembrane MLO family protein
-5.86	11.41	-2.55	12.69	0.02238487	AT3G21660	UBX domain-containing protein
-6.1	8.52	-2.61	9.82	0.01547273	AT3G21370	BGLU19__beta glucosidase 19
-6.17	9.18	-2.63	10.5	0.00294186	AT3G52720	ACA1_ATACA1_CAH1__alpha carbonic anhydrase 1
-6.49	13.45	-2.7	14.8	0.00896923	AT2G15020	unknown protein; BEST Arabidopsis thaliana protein match is: unknown protein (TAIR:AT5G64190.1); Has 72 Blast hits to 72 proteins in 10 species: Archae - 0; Bacteria - 0; Metazoa - 0; Fungi - 0; Plants - 72; Viruses - 0; Other Eukaryotes - 0 (source: NCBI BLink).
-6.58	8.19	-2.72	9.55	0.00196725	AT1G13650	BEST Arabidopsis thaliana protein match is: 18S pre-ribosomal assembly protein gar2-related (TAIR:AT2G03810.4); Has 3688 Blast hits to 1629 proteins in 255 species: Archae - 22; Bacteria - 222; Metazoa - 684; Fungi - 292; Plants - 62; Viruses - 14; Other Eukaryotes - 2392 (source: NCBI BLink). BEST Arabidopsis thaliana protein match is: 18S pre-ribosomal assembly protein gar2-related (TAIR:AT2G03810.4); Has 3688 Blast hits to 1629 proteins in 255 species: Archae - 22; Bacteria - 222; Metazoa - 684; Fungi - 292; Plants - 62; Viruses - 14; Other Eukaryotes - 2392 (source: NCBI BLink).
-6.76	9.5	-2.76	10.88	0.04244268	AT1G30390	
-6.86	10.68	-2.78	12.07	0.00887205	AT3G09450	CONTAINS InterPro DOMAIN/s: Fusaric acid resistance protein, conserved region (InterPro:IPR006726); BEST Arabidopsis thaliana protein match is: unknown protein (TAIR:AT2G28780.1); Has 503 Blast hits to 494 proteins in 215 species: Archae - 0; Bacteria - 423; Metazoa - 0; Fungi - 0; Plants - 65; Viruses - 0; Other Eukaryotes - 15 (source: NCBI BLink).

Supplementary Table S3. Gene Ontology analyses of NO-responsive genes.
http://www.geneontology.org/

Analysis Type:	PANTHER Overrepresentation Test (Released 20171205)						
Annotation Version and Release Date:	GO Ontology database Released 2017-12-27						
Analyzed List:	upload_1 NO-responsive at 1 h (Arabidopsis thaliana)						
Reference List:	Arabidopsis thaliana (all genes in database)						
Test Type:	FISHER						
GO biological process complete	Arabidopsis thaliana REFLIST (27502)	upload_1 1101	upload_1 (expected)	upload_1 (over/under)	upload_1 (fold Enrichment)	upload_1 (raw P-value)	upload_1 (FDR)
response to stimulus (GO:0050896)	5805	350	232.39	+	1.51	0.0000000000000000607	0.00000000000336
response to drug (GO:0042493)	529	62	21.18	+	2.93	0.00000000000107	0.00000000296
response to chitin (GO:0010200)	132	26	5.28	+	4.92	0.000000000396	0.00000073
response to chemical (GO:0042221)	2762	177	110.57	+	1.6	0.00000000136	0.00000188
defense response (GO:0006952)	1407	104	56.33	+	1.85	0.00000000848	0.00000938
response to ethylene (GO:0009723)	297	36	11.89	+	3.03	0.0000000283	0.0000261
response to organic substance (GO:0010033)	1947	129	77.95	+	1.66	0.0000000586	0.000036
response to stress (GO:0006950)	3283	195	131.43	+	1.48	0.0000000555	0.0000384
response to endogenous stimulus (GO:0009719)	1680	115	67.26	+	1.71	0.0000000801	0.0000402

response to hormone (GO:0009725)	1672	115	66.94	+	1.72	0.0000000544	0.000043
chromosome organization (GO:0051276)	428	0	17.13	-	< 0.01	0.0000000784	0.0000433
ethylene-activated signaling pathway (GO:0009873)	174	25	6.97	+	3.59	0.000000218	0.0000927
response to wounding (GO:0009611)	212	28	8.49	+	3.3	0.000000201	0.0000927
response to organonitrogen compound (GO:0010243)	190	26	7.61	+	3.42	0.000000294	0.000116
phosphorelay signal transduction system (GO:0000160)	214	27	8.57	+	3.15	0.000000741	0.000273
cellular response to ethylene stimulus (GO:0071369)	195	25	7.81	+	3.2	0.00000145	0.000502
response to nitrogen compound (GO:1901698)	297	32	11.89	+	2.69	0.00000172	0.00056
response to oxygen-containing compound (GO:1901700)	1506	100	60.29	+	1.66	0.00000251	0.000772
response to abiotic stimulus (GO:0009628)	1954	119	78.23	+	1.52	0.0000108	0.00297
oxidation-reduction process (GO:0055114)	1331	88	53.28	+	1.65	0.0000102	0.00298
response to light stimulus (GO:0009416)	699	54	27.98	+	1.93	0.0000126	0.00331
secondary metabolic process (GO:0019748)	475	41	19.02	+	2.16	0.0000136	0.00342
regulation of hormone levels (GO:0010817)	380	35	15.21	+	2.3	0.0000153	0.00368
response to decreased oxygen levels (GO:0036293)	70	13	2.8	+	4.64	0.0000165	0.00379
amide biosynthetic process (GO:0043604)	618	6	24.74	-	0.24	0.0000192	0.00379

response to oxygen levels (GO:0070482)	71	13	2.84	+	4.57	0.0000188	0.00385
signal transduction (GO:0007165)	1625	102	65.05	+	1.57	0.0000185	0.00393
response to radiation (GO:0009314)	726	55	29.06	+	1.89	0.000018	0.00398
signaling (GO:0023052)	1655	103	66.26	+	1.55	0.0000219	0.00417
secondary metabolite biosynthetic process (GO:0044550)	311	30	12.45	+	2.41	0.0000269	0.0048
response to acid chemical (GO:0001101)	1148	77	45.96	+	1.68	0.0000262	0.00482
chromatin organization (GO:0006325)	284	0	11.37	-	< 0.01	0.00003	0.00518
response to hypoxia (GO:0001666)	64	12	2.56	+	4.68	0.0000318	0.00533
cellular amide metabolic process (GO:0043603)	716	9	28.66	-	0.31	0.0000346	0.00562
cell communication (GO:0007154)	1888	113	75.58	+	1.5	0.0000393	0.00621
hormone biosynthetic process (GO:0042446)	184	21	7.37	+	2.85	0.000047	0.00722
organelle organization (GO:0006996)	1197	22	47.92	-	0.46	0.0000489	0.0073
intracellular signal transduction (GO:0035556)	505	41	20.22	+	2.03	0.000064	0.00932
peptide biosynthetic process (GO:0043043)	567	6	22.7	-	0.26	0.0000671	0.00951
translation (GO:0006412)	562	6	22.5	-	0.27	0.0000935	0.0129
isoprenoid catabolic process (GO:0008300)	15	6	0.6	+	9.99	0.000106	0.0136
terpenoid catabolic process (GO:0016115)	15	6	0.6	+	9.99	0.000106	0.0139

vesicle-mediated transport (GO:0016192)	369	2	14.77	-	0.14	0.000106	0.0143
organonitrogen compound biosynthetic process (GO:1901566)	1293	26	51.76	-	0.5	0.000126	0.0159
cellular response to hypoxia (GO:0071456)	26	7	1.04	+	6.73	0.000218	0.0268
response to external stimulus (GO:0009605)	1520	91	60.85	+	1.5	0.000236	0.0284
cellular response to decreased oxygen levels (GO:0036294)	28	7	1.12	+	6.24	0.000321	0.0362
cellular response to oxygen levels (GO:0071453)	28	7	1.12	+	6.24	0.000321	0.037
biological regulation (GO:0065007)	5517	271	220.86	+	1.23	0.000318	0.0374
cellular response to hormone stimulus (GO:0032870)	838	56	33.55	+	1.67	0.000377	0.0409
peptide metabolic process (GO:0006518)	639	9	25.58	-	0.35	0.000385	0.0409
response to jasmonic acid (GO:0009753)	211	21	8.45	+	2.49	0.000377	0.0417

Supplementary Table S4. TPT lines that were screened for differential effect of NO on hypocotyl elongation under darkness. The 968 TPT lines displayed in this table and expressing 363 TFs under a β-estradiol inducible promoter were screened for hypocotyl length in etiolated seedlings grown in media supplemented or not with 10 μM β-estradiol and each of them treated or not with 300 ppm NO

AGI code	Annotation	TPT lines					
AT1G67970	AT-HSFA8_HSFA8__heat shock transcription factor A8	1.67970.1B4	1.67970.1E3	1.67970.1F4			
AT2G18300	basic helix-loop-helix (bHLH) DNA-binding superfamily protein	2.18300.1B1	2.18300.1C3	2.18300.1I6			
AT2G33880	HB-3_STIP_WOX9_WOX9A__homeobox-3	2.33880.1A2	2.33880.1C6	2.33880.1E2			
AT2G37000	TCP family transcription factor	2.37000.1A3	2.37000.1B2				
AT3G04730	IAA16__indoleacetic acid-induced protein 16	3.04730.1A8	3.04730.1B2	3.04730.1D9			
AT3G16770	ATEBP_EBP_ERF72_RAP2.3__ethylene-responsive element binding protein	3.16770.1A9	3.16770.1C8	3.16770.1D7			
AT3G45150	TCP16__TCP domain protein 16	3.45150.1B2	3.45150.1I4				
AT4G11880	AGL14__AGAMOUS-like 14	4.11880.1D2	4.11880.1H4				
AT4G17490	ATERF6_ERF-6-6_ERF6__ethylene responsive element binding factor 6	4.17490.1A6	4.17490.1B8	4.17490.1D5	4.17490.1E1		
AT4G18880	AT-HSFA4A_HSF A4A__heat shock transcription factor A4A	4.18880.1C1	4.18880.1E2	4.18880.1F3			
AT4G36540	BEE2__BR enhanced expression 2	4.36540.1B8	4.36540.1C1	4.36540.1E4			
AT5G10140	AGL25_FLC_FLC_FLF__K-box region and MADS-box transcription factor family protein	5.10140.1B2	5.10140.1D5	5.10140.1E6			
AT5G18270	ANAC087__Arabidopsis NAC domain containing protein 87	5.18270.1A4	5.18270.1B8	5.18270.1C1			
AT5G23260	ABS_AGL32_TT16__K-box region and MADS-box transcription factor family protein	5.23260.1E2	5.23260.1F5	5.23260.1I3			
AT5G51870	AGL71__AGAMOUS-like 71	5.51870.1D7	5.51870.1E1	5.51870.1F8			
AT5G55690	MADS-box transcription factor family protein	5.55690.1A3	5.55690.1C4	5.55690.1G5			
AT5G65050	AGL31_MAF2__AGAMOUS-like 31	5.65050.1A2	5.65050.1C5	5.65050.1E4			
AT1G30210	ATTCP24_TCP24__TEOSINTE BRANCHED 1, cycloidea, and PCF family 24	1.30210.1D2	1.30210.1F5				
AT1G35560	TCP family transcription factor	1.35560.1C4	1.35560.1F1	1.35560.1G5			
AT1G50420	SCL-3_SCL3__scarecrow-like 3	1.50420.1A2	1.50420.1E5				
AT1G67260	TCP1__TCP family transcription factor	1.67260.1B5	1.67260.1C7	1.67260.1D4			
AT1G72010	TCP family transcription factor	1.72010.1D4	1.72010.1G9				
AT3G05200	ATL6__RING/U-box superfamily protein	3.05200.1B20	3.05200.1D17	3.05200.1J3	3.05200.1K7	3.05200.1L3	
AT3G27010	AT-TCP20_PCF1_ TEOSINTE BRANCHED 1, cycloidea, PCF (TCP)-domain family protein 20	3.27010.1A3	3.27010.1E2	3.27010.1F4	3.27010.1G1		
AT3G46600	GRAS family transcription factor	3.46600.1C4	3.46600.1D3	3.46600.1F3	3.46600.1G2		
AT3G60630	ATHAM2_HAM2_LOM2__GRAS family transcription factor	3.60630.1B4	3.60630.1C8	3.60630.1E8			
AT4G00050	UNE10__basic helix-loop-helix (bHLH) DNA-binding superfamily protein	4.00050.1F24	4.00050.1G2	4.00050.1J7			

AT4G18390	TCP2_TCP2__TEOSINTE BRANCHED 1, cycloidea and PCF transcription factor 2	4.18390.1H5					
AT4G18770	AtMYB98_MYB98__myb domain protein 98	4.18770.1A3	4.18770.1B6				
AT5G08070	TCP17__TCP domain protein 17	5.08070.1B3	5.08070.1D4				
AT5G11050	AtMYB64_MYB64__myb domain protein 64	5.11050.1D15					
AT5G49330	ATMYB111_MYB111_PFG3__myb domain protein 111	5.49330.1C5	5.49330.1D5	5.49330.1E6			
AT5G51910	TCP family transcription factor	5.51910.1B3	5.51910.1D1	5.51910.1G2	5.51910.1H2		
AT5G52510	SCL8__SCARECROW-like 8	5.52510.1B2	5.52510.1E3	5.52510.1H1			
AT5G60970	TCP5__TEOSINTE BRANCHED 1, cycloidea and PCF transcription factor 5	5.60970.1C8	5.60970.1F5	5.60970.1G7			
AT1G51950	IAA18__indole-3-acetic acid inducible 18	1.51950.1A9	1.51950.1B8				
AT1G52890	ANAC019_NAC019__NAC domain containing protein 19	1.52890.1B3	1.52890.1H2				
AT1G53910	RAP2.12__related to AP2 12	1.53910.1A6	1.53910.1B1	1.53910.1I4			
AT2G22630	AGL17__AGAMOUS-like 17	2.22630.1A4	2.22630.1C9	2.22630.1G9	2.22630.1H4		
AT3G04060	anac046_NAC046__NAC domain containing protein 46	3.04060.1B8	3.04060.1D4				
AT3G23050	AXR2_IAA7__indole-3-acetic acid 7	3.23050.1H3	3.23050.1I9				
AT3G57230	AGL16__AGAMOUS-like 16	3.57230.1A6	3.57230.1C8				
AT4G17500	ATERF-1_ERF-1__ethylene responsive element binding factor 1	4.17500.1B3	4.17500.1E2	4.17500.1I7			
AT4G27410	ANAC072_RD26	4.27410.1C2	4.27410.1D1				
AT4G34410	RRTF1__redox responsive transcription factor 1	4.34410.1C1	4.34410.1G1	4.34410.1H2			
AT4G36710	AtHAM4_HAM4__GRAS family transcription factor	4.36710.1B2	4.36710.1C6	4.36710.1D7	4.36710.1G3	4.36710.1H1	4.36710.1I9
AT5G11060	KNAT4__KNOTTED1-like homeobox gene 4	5.11060.1B3	5.11060.1D5	5.11060.1G4			
AT5G13790	AGL15__AGAMOUS-like 15	5.13790.1D4	5.13790.1F6	5.13790.1G3			
AT5G47230	ATERF-5_ATERF5_ERF5__ethylene responsive element binding factor 5	5.47230.1B1	5.47230.1C3	5.47230.1E4	5.47230.1G5		
AT5G53420	CCT motif family protein	5.53420.1F9	5.53420.1G2	5.53420.1I1			
AT5G62020	AT-HSFB2A_HSFB2A__heat shock transcription factor B2A	5.62020.1B4	5.62020.1F8	5.62020.1I3			
AT5G66160	ATRMR1_RMR1__receptor homology region transmembrane domain ring H2 motif protein 1	5.66160.1D1	5.66160.1G2				
AT1G01260	basic helix-loop-helix (bHLH) DNA-binding superfamily protein	1.01260.2C3	1.01260.2G5	1.01260.2I7			
AT1G63840	RING/U-box superfamily protein	1.63840.1E3	1.63840.1I5				
AT1G69490	ANAC029_ATNAP_NAP__NAC-like, activated by AP3/PI	1.69490.1A99	1.69490.1B99	1.69490.1C99	1.69490.1H99		
AT1G80400	RING/U-box superfamily protein	1.80400.1A3	1.80400.1G8	1.80400.1H6			
AT2G20180	PIF1_PIL5__phytochrome interacting factor 3-like 5	2.20180.1D5	2.20180.1F5	2.20180.1G6			
AT2G22750	basic helix-loop-helix (bHLH) DNA-binding superfamily protein	2.22750.2G6					
AT3G15500	ANAC055_ATNAC3_NAC055_NAC3__NAC domain containing protein 3	3.15500.1A99					
AT3G56970	BHLH038_ORG2__basic helix-loop-helix (bHLH) DNA-binding superfamily protein	3.56970.1D99	3.56970.1E99	3.56970.1G99	3.56970.1H99		
AT3G61150	HD-GL2-1_HDG1__homeodomain GLABROUS 1	3.61150.1A3	3.61150.1B6	3.61150.1D7			
AT3G61460	BRH1__brassinosteroid-responsive RING-H2	3.61460.1E6	3.61460.1G7	3.61460.1H2			
AT3G62690	ATL5__AtL5	3.62690.1F8	3.62690.1K3	3.62690.1Q8			

AT4G09180	basic helix-loop-helix (bHLH) DNA-binding superfamily protein	4.09180.1B2	4.09180.1C8				
AT4G11680	Zinc finger, C3HC4 type (RING finger) family protein	4.11680.1F7	4.11680.1G9	4.11680.1I2			
AT4G14550	IAA14_SLR__indole-3-acetic acid inducible 14	4.14550.1B9	4.14550.1C2	4.14550.1J9			
AT4G36060	bHLH11__basic helix-loop-helix (bHLH) DNA-binding superfamily protein	4.36060.1A7	4.36060.1C8	4.36060.1H7			
AT5G02470	DPA__Transcription factor DP	5.02470.1D99	5.02470.1E99	5.02470.1F99			
AT5G03510	C2H2-type zinc finger family protein	5.03510.1B99	5.03510.1F99	5.03510.1G99			
AT5G10380	ATRING1_RING1__RING/U-box superfamily protein	5.10380.1B8	5.10380.1C7	5.10380.1I2			
AT1G69180	CRC__Plant-specific transcription factor YABBY family protein	1.69180.1G1	1.69180.1I1				
AT1G70510	ATK1_KNAT2__KNOTTED-like from Arabidopsis thaliana 2	1.70510.1B1	1.70510.1E7	1.70510.1F5			
AT2G18670	RING/U-box superfamily protein	2.18670.1A1	2.18670.1B4				
AT2G22850	AtbZIP6_bZIP6__basic leucine-zipper 6	2.22850.1A3	2.22850.1E1	2.22850.1F1	2.22850.1H9		
AT2G26580	YAB5__plant-specific transcription factor YABBY family protein	2.26580.1B1					
AT2G45190	AFO_FIL_YAB1__Plant-specific transcription factor YABBY family protein	2.45190.1C3	2.45190.1F3				
AT2G45680	TCP family transcription factor	2.45680.1A4	2.45680.1D2				
AT3G07650	COL9__CONSTANS-like 9	3.07650.1D6	3.07650.1F8				
AT3G12820	AtMYB10_MYB10__myb domain protein 10	3.12820.1A5	3.12820.1G1	3.12820.1H1			
AT3G15170	ANAC054_ATNAC1_CUC1	3.15170.1B6	3.15170.1C8	3.15170.1G5			
AT3G18400	anac058_NAC058__NAC domain containing protein 58	3.18400.1D3	3.18400.1G9				
AT3G62550	Adenine nucleotide alpha hydrolases-like superfamily protein	3.62550.1A1	3.62550.1C2				
AT4G23750	CRF2_TMO3__cytokinin response factor 2	4.23750.2B3	4.23750.2C8	4.23750.2F7			
AT5G04410	anac078_NAC2__NAC domain containing protein 2	5.04410.1D6	5.04410.1G6				
AT5G06950	AHBP-1B_TGA2__bZIP transcription factor family protein	5.06950.1C5	5.06950.1D9	5.06950.1I7			
AT5G49450	AtbZIP1_bZIP1__basic leucine-zipper 1	5.49450.1C1	5.49450.1D4				
AT1G07530	ATGRAS2_GRAS2_SCL14__SCARECROW-like 14	1.07530.1B99	1.07530.1E99				
AT1G55580	LAS_SCL18__GRAS family transcription factor	1.55580.1A3	1.55580.1D9				
AT1G55760	BTB/POZ domain-containing protein	1.55760.1A5	1.55760.1C5	1.55760.1E4			
AT2G17180	DAZ1__C2H2-like zinc finger protein	2.17180.1E6	2.17180.1F3	2.17180.1H4			
AT2G24790	ATCOL3_COL3__CONSTANS-like 3	2.24790.1A3	2.24790.1H2				
AT2G25000	ATWRKY60_WRKY60__WRKY DNA-binding protein 60	2.25000.1C5	2.25000.1D5	2.25000.1E6	2.25000.1H4		
AT2G33710	Integrase-type DNA-binding superfamily protein	2.33710.1C1	2.33710.1E7	2.33710.1G2	2.33710.1H6		
AT2G38340	DREB19__Integrase-type DNA-binding superfamily protein	2.38340.1B7	2.38340.1I9				
AT3G01220	ATHB20_HB20__homeobox protein 20	3.01220.1D1	3.01220.1E2	3.01220.1I6			
AT3G03450	RGL2__RGA-like 2	3.03450.1C99	3.03450.1F99	3.03450.1H99			
AT3G10480	ANAC050_NAC050__NAC domain containing protein 50	3.10480.1D3	3.10480.1E3				
AT3G11580	AP2/B3-like transcriptional factor family protein	3.11580.1A5	3.11580.1E6				
AT4G31800	ATWRKY18_WRKY18__WRKY DNA-binding protein 18	4.31800.1C99	4.31800.1E99				

AT4G36900	DEAR4_RAP2.10__related to AP2 10	4.36900.1A3	4.36900.1B2	4.36900.1E1	4.36900.1I2		
AT5G03790	ATHB51_HB51_LMI1__homeobox 51	5.03790.1E8	5.03790.1F1				
AT5G13080	ATWRKY75_WRKY75__WRKY DNA-binding protein 75	5.13080.1G9	5.13080.1H5				
AT5G22220	ATE2FB_E2F1_E2FB__E2F transcription factor 1	5.22220.1D6	5.22220.1E7	5.22220.1I4			
AT5G43290	ATWRKY49_WRKY49__WRKY DNA-binding protein 49	5.43290.1A6	5.43290.1D2				
AT1G56160	ATMYB72_MYB72__myb domain protein 72	1.56160.1B6	1.56160.1C2	1.56160.1E2	1.56160.1G5		
AT1G68520	B-box type zinc finger protein with CCT domain	1.68520.1H3					
AT1G71130	CRF8__Integrase-type DNA-binding superfamily protein	1.71130.1C4	1.71130.1F4	1.71130.1H9			
AT1G71450	Integrase-type DNA-binding superfamily protein	1.71450.1D2	1.71450.1I5				
AT1G79180	ATMYB63_MYB63__myb domain protein 63	1.79180.1E3					
AT3G01970	ATWRKY45_WRKY45__WRKY DNA-binding protein 45	3.01970.1C5	3.01970.1D6	3.01970.1F5			
AT3G05700	Drought-responsive family protein	3.05700.1D4	3.05700.1E1	3.05700.1F4	3.05700.1G6		
AT3G11260	WOX5_WOX5B__WUSCHEL related homeobox 5	3.11260.1A4	3.11260.1B7	3.11260.1E9			
AT3G16280	Integrase-type DNA-binding superfamily protein	3.16280.1A4	3.16280.1B3				
AT3G23240	ATERF1_ERF1__ethylene response factor 1	3.23240.1D4	3.23240.1I2				
AT4G13640	UNE16__Homeodomain-like superfamily protein	4.13640.1C3	4.13640.1D5	4.13640.1G5	4.13640.1I1		
AT4G28270	ATRMA2_RMA2__RING membrane-anchor 2	4.28270.1B6	4.28270.1C6	4.28270.1E6	4.28270.1F3		
AT4G35610	zinc finger (C2H2 type) family protein	4.35610.1A3	4.35610.1B3	4.35610.1D2	4.35610.1F6		
AT5G05410	DREB2_DREB2A__DRE-binding protein 2A	5.05410.1C5	5.05410.1E2	5.05410.1H5	5.05410.1I6		
AT5G17300	RVE1__Homeodomain-like superfamily protein	5.17300.1A3	5.17300.1B9	5.17300.1I8			
AT5G18450	Integrase-type DNA-binding superfamily protein	5.18450.1B6					
AT5G20240	PI__K-box region and MADS-box transcription factor family protein	5.20240.1A5	5.20240.1B6	5.20240.1F5			
AT5G25890	IAA28_IAR2__indole-3-acetic acid inducible 28	5.25890.1F4					
AT1G18710	AtMYB47_MYB47__myb domain protein 47	1.18710.1A9	1.18710.1E3				
AT1G21450	SCL1__SCARECROW-like 1	1.21450.1A4	1.21450.1D10	1.21450.1G9			
AT1G47760	AGL102__AGAMOUS-like 102	1.47760.1H7					
AT1G66230	AtMYB20_MYB20__myb domain protein 20	1.66230.1C2	1.66230.1G2				
AT2G18550	ATHB21_HB-2_HB21__homeobox protein 21	2.18550.1C4	2.18550.1E1	2.18550.1I4			
AT2G22800	HAT9__Homeobox-leucine zipper protein family	2.22800.1A3	2.22800.1B2	2.22800.1D1	2.22800.1G4		
AT2G31180	ATMYB14_MYB14_MYB14AT__myb domain protein 14	2.31180.1A1	2.31180.1D7	2.31180.1G8			
AT2G44940	Integrase-type DNA-binding superfamily protein	2.44940.1A1	2.44940.1B6	2.44940.1E3	2.44940.1F2	2.44940.1G5	
AT2G45660	AGL20_ATSOC1_SOC1__AGAMOUS-like 20	2.45660.1I5					
AT3G03200	anac045_NAC045__NAC domain containing protein 45	3.03200.1A4	3.03200.1C2	3.03200.1G2			
AT3G21890	B-box type zinc finger family protein	3.21890.1A8	3.21890.1B5	3.21890.1H8			
AT4G05100	AtMYB74_MYB74__myb domain protein 74	4.05100.1D5	4.05100.1H9				
AT4G39070	B-box zinc finger family protein	4.39070.1C2	4.39070.1E3	4.39070.1F9			

AT5G05770	WOX5A_WOX7__WUSCHEL related homeobox 7	5.05770.1A9	5.05770.1B9	5.05770.1E8			
AT5G15150	ATHB-3_ATHB3_HAT7_HB-3__homeobox 3	5.15150.1H3	5.15150.1I1				
AT5G25810	tny__Integrase-type DNA-binding superfamily protein	5.25810.1B9	5.25810.1C9	5.25810.1H7			
AT5G65230	AtMYB53_MYB53__myb domain protein 53	5.65230.1I4					
AT1G15050	IAA34__indole-3-acetic acid inducible 34	1.15050.1C6					
AT1G62975	basic helix-loop-helix (bHLH) DNA-binding superfamily protein	1.62975.1A2	1.62975.1B2	1.62975.1D4	1.62975.1F4	1.62975.1H4	1.62975.1I6
AT1G66350	RGL_RGL1__RGA-like 1	1.66350.1A4	1.66350.1E8	1.66350.1F3			
AT2G03710	AGL3_SEP4__K-box region and MADS-box transcription factor family protein	2.03710.1A6	2.03710.1E3	2.03710.1G5			
AT2G33480	ANAC041_NAC041__NAC domain containing protein 41	2.33480.1G7	2.33480.1H1				
AT2G34140	Dof-type zinc finger DNA-binding family protein	2.34140.1C6	2.34140.1D1	2.34140.1G1	2.34140.1H1		
AT2G37740	ATZFP10_ZFP10__zinc-finger protein 10	2.37740.1A5.6	2.37740.1H2				
AT3G10490	ANAC051_ANAC052_NAC052__NAC domain containing protein 52	3.10490.1A1	3.10490.1C3	3.10490.1D3	3.10490.1E4	3.10490.1F2	3.10490.1H3
AT3G10760	Homeodomain-like superfamily protein	3.10760.1C7	3.10760.1D3	3.10760.1E3			
AT3G52440	Dof-type zinc finger DNA-binding family protein	3.52440.1B6	3.52440.1D9	3.52440.1I4			
AT4G03510	ATRMA1_RMA1__RING membrane-anchor 1	4.03510.1H5	4.03510.1I4				
AT4G17460	HAT1__Homeobox-leucine zipper protein 4 (HB-4) / HD-ZIP protein	4.17460.1B1	4.17460.1C8	4.17460.1E8	4.17460.1G1		
AT4G36740	ATHB40_HB-5_HB40__homeobox protein 40	4.36740.1C6	4.36740.1H2				
AT5G25220	KNAT3__KNOTTED1-like homeobox gene 3	5.25220.1A3	5.25220.1B1	5.25220.1C3	5.25220.1E2	5.25220.1G3	5.25220.1H5
AT5G62380	ANAC101_NAC101_VND6__NAC-domain protein 101	5.62380.1D3	5.62380.1F6	5.62380.1G2			
AT1G08465	YAB2__Plant-specific transcription factor YABBY family protein	1.08465.1F2	1.08465.1H2	1.08465.1I7			
AT1G08780	AIP3_PFD4__ABI3-interacting protein 3	1.08780.1F8	1.08780.1I5				
AT1G14900	HMGA__high mobility group A HMGA__high mobility group A	1.14900.1C2	1.14900.1G9	1.14900.1H7			
AT1G54330	ANAC020_NAC020__NAC domain containing protein 20	1.54330.1A10	1.54330.1C3	1.54330.1I2			
AT1G68880	AtbZIP_bZIP__basic leucine-zipper 8	1.68880.1A3	1.68880.1G5				
AT2G43000	anac042_NAC042__NAC domain containing protein 42	2.43000.1D1	2.43000.1H2	2.43000.1I2			
AT3G46640	LUX_PCL1__Homeodomain-like superfamily protein	3.46640.1C99	3.46640.1D99	3.46640.1G99			
AT3G47620	AtTCP14_TCP14__TEOSINTE BRANCHED, cycloidea and PCF (TCP) 14	3.47620.1D3	3.47620.1E7				
AT3G52270	Transcription initiation factor IIF, beta subunit	3.52270.1F5	3.52270.1H2	3.52270.1I1			
AT4G08150	BP_BP1_KNAT1__KNOTTED-like from Arabidopsis thaliana	4.08150.1A4	4.08150.1E4	4.08150.1F1	4.08150.1G12		
AT4G32040	KNAT5__KNOTTED1-like homeobox gene 5	4.32040.1F3	4.32040.1H5				
AT4G35280	DAZ2__C2H2-like zinc finger protein	4.35280.1A99	4.35280.1C99	4.35280.1D99	4.35280.1F99	4.35280.1H99	
AT4G38000	DOF4.7__DNA binding with one finger 4.7	4.38000.1C13	4.38000.1E13				
AT5G08790	anac081_ATAF2	5.08790.1B99	5.08790.1H99				
AT5G15840	CO_FG__B-box type zinc finger protein with CCT domain	5.15840.1C99	5.15840.1F99	5.15840.1I99			
AT5G45580	Homeodomain-like superfamily protein	5.45580.1B99	5.45580.1C99	5.45580.1G99			
AT5G64810	ATWRKY51_WRKY51__WRKY DNA-binding protein 51	5.64810.1F3	5.64810.1G1	5.64810.1H1			

AT1G06040	BBX24_STO__B-box zinc finger family protein	1.06040.1C6					
AT1G06850	AtbZIP52_bZIP52__basic leucine-zipper 52	1.06850.1B2	1.06850.1C9	1.06850.1G9			
AT1G26960	AtHB23_HB23__homeobox protein 23	1.26960.1A9	1.26960.1F9	1.26960.1H8			
AT1G54140	TAF9_TAFII21__TATA binding protein associated factor 21kDa subunit	1.54140.1D2	1.54140.1E1				
AT1G66600	ABO3_ATWRKY63_WRKY63__ABA overly sensitive mutant 3	1.66600.1F5	1.66600.1G5				
AT2G04038	AtbZIP48_bZIP48__basic leucine-zipper 48	2.04038.1C9	2.04038.1F9				
AT2G36610	ATHB22_HB22__homeobox protein 22	2.36610.1E5	2.36610.1F4	2.36610.1H3			
AT3G04030	Homeodomain-like superfamily protein	3.04030.3C5	3.04030.3D1	3.04030.3F4			
AT3G12890	ASML2__activator of spomin::LUC2	3.12890.1A9	3.12890.1D7	3.12890.1I2			
AT3G13540	ATMYB5_MYB5__myb domain protein 5	3.13540.1B1	3.13540.1D4	3.13540.1F5			
AT3G21880	B-box type zinc finger protein with CCT domain	3.21880.1A1	3.21880.1E5				
AT3G57480	zinc finger (C2H2 type, AN1-like) family protein	3.57480.1C9	3.57480.1D5	3.57480.1I1			
AT3G61890	ATHB-12_ATHB12_HB-12__homeobox 12	3.61890.1A3	3.61890.1B3				
AT4G08250	GRAS family transcription factor	4.08250.1B99	4.08250.1C99	4.08250.1G99	4.08250.1H99		
AT4G15250	B-box type zinc finger protein with CCT domain	4.15250.1E9	4.15250.1G6				
AT4G21050	Dof-type zinc finger domain-containing protein	4.21050.1C9	4.21050.1G6				
AT5G60890	ATMYB34_ATR1_MYB34__myb domain protein 34	5.60890.1C6	5.60890.1D9	5.60890.1H9			
AT5G64060	anac103_NAC103__NAC domain containing protein 103	5.64060.1E99	5.64060.1G99	5.64060.1H3	5.64060.1I99		
AT1G08320	bZIP21_TGA9__bZIP transcription factor family protein	1.08320.1F3	1.08320.1G8	1.08320.1I9			
AT1G13600	AtbZIP58_bZIP58__basic leucine-zipper 58	1.13600.1C3	1.13600.1G7	1.13600.1I7			
AT1G20693	HMG BETA 1_HMGB2_NFD02_NFD2__high mobility group B2	1.20693.1E4	1.20693.1F1	1.20693.1H9			
AT1G28520	ATVOZ1_VOZ1__vascular plant one zinc finger protein	1.28520.1A1	1.28520.1D2	1.28520.1F3			
AT1G59640	BPE_BPEp_BPEub_ZCW32__BIG PETAL P	1.59640.1E7	1.59640.1F5	1.59640.1G6			
AT1G75510	Transcription initiation factor IIF, beta subunit	1.75510.1B99	1.75510.1C99	1.75510.1E99	1.75510.1G99		
AT2G01060	myb-like HTH transcriptional regulator family protein	2.01060.1A1	2.01060.1F7	2.01060.1G7			
AT3G18990	REM39_VRN1__AP2/B3-like transcriptional factor family protein	3.18990.1A3	3.18990.1C1	3.18990.1H1			
AT3G30260	AGL79__AGAMOUS-like 79	3.30260.1B6	3.30260.1E9	3.30260.1H2			
AT3G50650	GRAS family transcription factor	3.50650.1A4	3.50650.1B4	3.50650.1E1	3.50650.1I6		
AT3G56530	anac064_NAC064__NAC domain containing protein 64	3.56530.1C5	3.56530.1E6	3.56530.1F1			
AT4G17230	SCL13__SCARECROW-like 13	4.17230.1A2	4.17230.1C6	4.17230.1D5			
AT4G35570	HMGB5_HMGD_NFD05_NFD5__high mobility group B5	4.35570.1B8	4.35570.1D3	4.35570.1I4			
AT4G37750	ANT_CKC_CKC1_DRG__Integrase-type DNA-binding superfamily protein	4.37750.1A8	4.37750.1E7	4.37750.1F2			
AT5G48150	PAT1__GRAS family transcription factor	5.48150.1A99	5.48150.1G15	5.48150.1H99			
AT5G48250	B-box type zinc finger protein with CCT domain	5.48250.1B6	5.48250.1C2	5.48250.1G1			
AT5G60850	OBP4__OBF binding protein 4	5.60850.1B5	5.60850.1C7	5.60850.1G9			
AT5G65590	Dof-type zinc finger DNA-binding family protein	5.65590.1F99	5.65590.1I99				

AT1G08810	AtMYB60_MYB60__myb domain protein 60	1.08810.1C1	1.08810.1H2				
AT1G25440	B-box type zinc finger protein with CCT domain	1.25440.1B4	1.25440.1E7	1.25440.1H9			
AT1G27730	STZ_ZAT10__salt tolerance zinc finger	1.27730.1E5	1.27730.1I1				
AT1G42990	ATBZIP60_BZIP60_BZIP60__basic region/leucine zipper motif 60	1.42990.1A2	1.42990.1D8				
AT1G43160	RAP2.6__related to AP2 6	1.43160.1E4	1.43160.1H2	1.43160.1I1			
AT1G51600	GATA28_TIFY2A_ZML2__ZIM-LIKE 2	1.51600.2B99	1.51600.2E99	1.51600.2G99			
AT1G56170	ATHAP5B_HAP5B_NF-YC2__nuclear factor Y, subunit C2	1.56170.1C99	1.56170.1F99	1.56170.1H99			
AT1G59530	ATBZIP4_bZIP4__basic leucine-zipper 4	1.59530.1B1	1.59530.1E1	1.59530.1G2			
AT1G60250	B-box zinc finger family protein	1.60250.1A99	1.60250.1C99	1.60250.1H99			
AT1G69690	AtTCP15_TCP15__TCP family transcription factor	1.69690.1A9	1.69690.1B1	1.69690.1C1			
AT2G20570	ATGLK1_GLK1_GPRI1__GBF's pro-rich region-interacting factor 1	2.20570.1B4					
AT2G31720	Domain of unknown function (DUF313)	2.31720.1A99	2.31720.1C99	2.31720.1D99			
AT2G43220	Cysteine/Histidine-rich C1 domain family protein	2.43220.1B9	2.43220.1G9	2.43220.1H1			
AT3G61910	ANAC066_NAC066_NST2__NAC domain protein 66	3.61910.1B2	3.61910.1H3	3.61910.1I4			
AT4G38140	RING/U-box superfamily protein	4.38140.1F7					
AT5G50470	NF-YC7__nuclear factor Y, subunit C7	5.50470.1A1	5.50470.1B8	5.50470.1D11	5.50470.1E5		
AT5G53980	ATHB52_HB52__homeobox protein 52	5.53980.1E5	5.53980.1F2	5.53980.1G2			
AT1G17310	MADS-box transcription factor family protein	1.17310.1A5	1.17310.1F1				
AT1G25340	AtMYB116_MYB116__myb domain protein 116	1.25340.1D7					
AT1G59750	ARF1__auxin response factor 1	1.59750.1B3	1.59750.1C5				
AT2G31380	STH__salt tolerance homologue	2.31380.1A3	2.31380.1B1	2.31380.1F3			
AT2G40340	AtERF48_DREB2C__Integrase-type DNA-binding superfamily protein	2.40340.1H5					
AT2G47700	RFI2__RING/U-box superfamily protein	2.47700.1E1	2.47700.1G4	2.47700.1I2			
AT3G13445	TBP1_TFIID-1__TATA binding protein 1	3.13445.1A1	3.13445.1D1	3.13445.1F4			
AT3G21270	ADOF2_DOF2__DOF zinc finger protein 2	3.21270.1H1	3.21270.1I1				
AT4G30935	ATWRKY32_WRKY32__WRKY DNA-binding protein 32	4.30935.1H6	4.30935.1I8				
AT4G38960	B-box type zinc finger family protein	4.38960.1A3	4.38960.1G3				
AT5G04640	AGL99__AGAMOUS-like 99	5.04640.1A6	5.04640.1D6				
AT5G13330	Rap2.6L__related to AP2 6l	5.13330.1D9	5.13330.1G9	5.13330.1I4			
AT5G26990	Drought-responsive family protein	5.26990.1C10	5.26990.1E6				
AT5G47220	ATERF-2_ATERF2_ERF2__ethylene responsive element binding factor 2	5.47220.1A7	5.47220.1G3				
AT5G50490	NF-YC5__nuclear factor Y, subunit C5	5.50490.1B6	5.50490.1F8	5.50490.1H9			
AT5G57620	AtMYB36_MYB36__myb domain protein 36	5.57620.1C3	5.57620.1F4				
AT5G66940	Dof-type zinc finger DNA-binding family protein	5.66940.1D4	5.66940.1H6				
AT1G01010	ANAC001_NAC001__NAC domain containing protein 1	1.01010.1E5	1.01010.1F9	1.01010.1G3			
AT1G09530	PAP3_PIF3_POC1__phytochrome interacting factor 3	1.09530.1F4					

AT1G12610	DDF1__Integrase-type DNA-binding superfamily protein	1.12610.1B2	1.12610.1D9	1.12610.1E4				
AT1G13300	HRS1__myb-like transcription factor family protein	1.13300.1A9	1.13300.1D3	1.13300.1E5				
AT1G18570	AtMYB51_BW51A_BW51B_HIG1_MYB51__myb domain protein 51	1.18570.1F7						
AT1G56650	ATMYB75_MYB75_PAP1_SIAA1__production of anthocyanin pigment 1	1.56650.1C5	1.56650.1H3					
AT1G68190	B-box zinc finger family protein	1.68190.1D1						
AT1G71030	ATMYBL2_MYBL2__MYB-like 2	1.71030.1C5	1.71030.1H9					
AT1G74930	ORA47__Integrase-type DNA-binding superfamily protein	1.74930.1E2	1.74930.1F8	1.74930.1H4				
AT2G13570	NF-YB7__nuclear factor Y, subunit B7	2.13570.1A1	2.13570.1C3	2.13570.1I2				
AT2G22200	Integrase-type DNA-binding superfamily protein	2.22200.1B1	2.22200.1E7	2.22200.1G8				
AT2G40970	MYBC1__Homeodomain-like superfamily protein	2.40970.1F1	2.40970.1H3					
AT3G28910	ATMYB30_MYB30__myb domain protein 30	3.28910.1C1	3.28910.1E5					
AT4G18020	APRR2_PRR2__CheY-like two-component responsive regulator family protein	4.18020.1B9	4.18020.1F4					
AT4G22680	AtMYB85_MYB85__myb domain protein 85	4.22680.1F1						
AT4G29080	IAA27_PAP2__phytochrome-associated protein 2	4.29080.1B2	4.29080.1I5					
AT5G62320	ATMYB99_ATMYBCU15_MYB99__myb domain protein 99	5.62320.1B8	5.62320.1C5	5.62320.1G1				
AT5G67190	DEAR2__DREB and EAR motif protein 2	5.67190.1C4	5.67190.1F5	5.67190.1G6				
AT1G17590	NF-YA8__nuclear factor Y, subunit A8	1.17590.1B5	1.17590.1F5					
AT1G68920	basic helix-loop-helix (bHLH) DNA-binding superfamily protein	1.68920.2A4	1.68920.2F3					
AT1G69120	AGL7_AP1__K-box region and MADS-box transcription factor family protein	1.69120.1B3	1.69120.1D2					
AT1G69780	ATHB13__Homeobox-leucine zipper protein family	1.69780.1D6	1.69780.1F9	1.69780.1H2				
AT1G71930	ANAC030_VND7__vascular related NAC-domain protein 7	1.71930.1B4	1.71930.1C9	1.71930.1F9				
AT1G73410	ATMYB54_MYB54__myb domain protein 54	1.73410.1G9	1.73410.1H6					
AT1G75410	BLH3__BEL1-like homeodomain 3	1.75410.2B7	1.75410.2C5	1.75410.2D6				
AT1G77850	ARF17__auxin response factor 17	1.77850.1A5	1.77850.1G5	1.77850.1I7				
AT2G20400	myb-like HTH transcriptional regulator family protein	2.20400.1B1	2.20400.1C9	2.20400.1I9				
AT2G44910	ATHB-4_ATHB4_HB4__homeobox-leucine zipper protein 4	2.44910.1A3	2.44910.1C5	2.44910.1G6				
AT3G12730	Homeodomain-like superfamily protein	3.12730.1D5	3.12730.1E3	3.12730.1F2				
AT3G29035	ANAC059_ATNAC3_NAC3_ORS1__NAC domain containing protein 3	3.29035.1C2	3.29035.1D9	3.29035.1H8	3.29035.1I2			
AT3G61250	AtMYB17_LMI2_MYB17__myb domain protein 17	3.61250.1A6	3.61250.1F4	3.61250.1G2	3.61250.1I3			
AT4G01250	AtWRKY22_WRKY22__WRKY family transcription factor	4.01250.1C4	4.01250.1E9	4.01250.1F1				
AT4G02220	zinc finger (MYND type) family protein / programmed cell death 2 C-terminal domain-containing protein	4.02220.1C6	4.02220.1E5	4.02220.1G5	4.02220.1H4			
AT5G29000	PHL1__Homeodomain-like superfamily protein	5.29000.1C3	5.29000.1D9	5.29000.1G5				
AT5G46590	anac096_NAC096__NAC domain containing protein 96	5.46590.1D5	5.46590.1G6	5.46590.1I5				
AT5G57660	ATCOL5_COL5__CONSTANS-like 5	5.57660.1A3	5.57660.1B9	5.57660.1H4				
AT1G08010	GATA11__GATA transcription factor 11	1.08010.2H4						

AT1G25560	EDF1_TEM1__AP2/B3 transcription factor family protein	1.25560.1C1	1.25560.1F2				
AT1G44830	Integrase-type DNA-binding superfamily protein	1.44830.1A99	1.44830.1F99	1.44830.1G99			
AT1G48000	AtMYB112_MYB112__myb domain protein 112	1.48000.2B9	1.48000.2C8	1.48000.2H1			
AT1G78600	BBX22_DBB3_LZF1_STH3__light-regulated zinc finger protein 1	1.78600.1D3	1.78600.1E8	1.78600.1F9			
AT2G28910	CXIP4__CAX interacting protein 4	2.28910.1C6	2.28910.1D3	2.28910.1E3			
AT2G31370	Basic-leucine zipper (bZIP) transcription factor family protein	2.31370.1B7	2.31370.1D8				
AT2G33290	ATSUVH2_SDG3_SUVH2__SU(VAR)3-9 homolog 2	2.33290.1A8	2.33290.1G7	2.33290.1H7			
AT3G04450	Homeodomain-like superfamily protein	3.04450.1C7	3.04450.1F7				
AT3G25730	EDF3__ethylene response DNA binding factor 3	3.25730.1A5	3.25730.1G6				
AT3G49690	ATMYB84_MYB84_RAX3__myb domain protein 84	3.49690.1A3					
AT3G49950	GRAS family transcription factor	3.49950.1F3					
AT3G55730	AtMYB109_MYB109__myb domain protein 109	3.55730.1A8	3.55730.1F9	3.55730.1G1			
AT5G02460	Dof-type zinc finger DNA-binding family protein	5.02460.1D2					
AT5G41570	ATWRKY24_WRKY24__WRKY DNA-binding protein 24	5.41570.1B1	5.41570.1F7				
AT5G47140	GATA27__GATA transcription factor 27	5.47140.1A99	5.47140.1C99	5.47140.1F99	5.47140.1I99		
AT5G63470	NF-YC4__nuclear factor Y, subunit C4	5.63470.1A4	5.63470.1B9	5.63470.1F1			
AT5G66700	ATHB53_HB-8_HB53__homeobox 53	5.66700.1E7	5.66700.1G2	5.66700.1H9			
AT1G14900	HMGA__high mobility group A HMGA__high mobility group A	1.14900.1B9	1.14900.1D5	1.14900.1I7			
AT1G55520	ATTBP2_TBP2__TATA binding protein 2	1.55520.2A3	1.55520.2D1				
AT1G65360	AGL23__AGAMOUS-like 23	1.65360.1A5	1.65360.1G2	1.65360.1H1			
AT1G68640	PAN__bZIP transcription factor family protein	1.68640.1E9	1.68640.1F6				
AT1G77920	bZIP transcription factor family protein	1.77920.1B3	1.77920.1E1	1.77920.1G3	1.77920.1H2		
AT2G04890	SCL21__SCARECROW-like 21	2.04890.1C5	2.04890.1F5				
AT2G45160	ATHAM1_HAM1_LOM1__GRAS family transcription factor	2.45160.1C3	2.45160.1E1	2.45160.1H4			
AT3G30530	ATBZIP42_bZIP42__basic leucine-zipper 42	3.30530.1C2	3.30530.1E3				
AT3G46090	ZAT7__C2H2 and C2HC zinc fingers superfamily protein	3.46090.1B99	3.46090.1E99	3.46090.1H99	3.46090.1I99		
AT3G53200	AtMYB27_MYB27__myb domain protein 27	3.53200.1F2					
AT4G24540	AGL24__AGAMOUS-like 24	4.24540.1G2	4.24540.1I5				
AT5G04940	SUVH1__SU(VAR)3-9 homolog 1	5.04940.2D9	5.04940.2G4				
AT5G09740	HAM2__histone acetyltransferase of the MYST family 2	5.09740.2D2	5.09740.2I9				
AT5G17490	AtRGL3_RGL3__RGA-like protein 3	5.17490.1C4	5.17490.1E9	5.17490.1F2			
AT5G27910	NF-YC8__nuclear factor Y, subunit C8	5.27910.1D2	5.27910.1G1				
AT5G35550	ATMYB123_ATTT2_MYB123_TT2__Duplicated homeodomain-like superfamily protein	5.35550.1A4	5.35550.1C7				
AT5G49420	MADS-box transcription factor family protein	5.49420.1A3	5.49420.1B3	5.49420.1H1			
AT5G67060	HEC1__basic helix-loop-helix (bHLH) DNA-binding superfamily protein	5.67060.1A1	5.67060.1B5	5.67060.1F7			
AT1G29160	Dof-type zinc finger DNA-binding family protein	1.29160.1B3	1.29160.1F3	1.29160.1G7			

AT1G59810	AGL50__AGAMOUS-like 50	1.59810.1D6					
AT1G71520	Integrase-type DNA-binding superfamily protein	1.71520.1A3					
AT2G32460	ATM1_ATMYB101_MYB101__myb domain protein 101	2.32460.1F3					
AT2G34000	RING/U-box superfamily protein	2.34000.1A5	2.34000.1H6				
AT2G36450	HRD__Integrase-type DNA-binding superfamily protein	2.36450.1E6	2.36450.1G6				
AT2G46960	CYP709B1__cytochrome P450, family 709, subfamily B, polypeptide 1	2.46960.1B3	2.46960.1I5	2.46960.1K5			
AT2G47190	ATMYB2_MYB2__myb domain protein 2	2.47190.1H3					
AT3G02310	AGL4_SEP2__K-box region and MADS-box transcription factor family protein	3.02310.1E1	3.02310.1F1	3.02310.1H1			
AT3G03550	RING/U-box superfamily protein	3.03550.1B5					
AT3G49530	ANAC062_NAC062_NTL6__NAC domain containing protein 62	3.49530.1C9	3.49530.1E9	3.49530.1H3			
AT4G20280	TAF11__TBP-associated factor 11	4.20280.1C99	4.20280.1D99	4.20280.1H99	4.20280.1I99		
AT4G31610	ATREM1_REM1__Transcriptional factor B3 family protein	4.31610.1B99	4.31610.1C99	4.31610.1E99	4.31610.1F99	4.31610.1H99	4.31610.1I99
AT5G12870	ATMYB46_MYB46__myb domain protein 46	5.12870.1D1	5.12870.1E1	5.12870.1I1			
AT5G16600	AtMYB43_MYB43__myb domain protein 43	5.16600.1F4					
AT5G28770	AtbZIP63_BZO2H3__bZIP transcription factor family protein	5.28770.1C9	5.28770.1H9	5.28770.1I8			
AT1G09540	ATMYB61_MYB61__myb domain protein 61	1.09540.1B4	1.09540.1D5	1.09540.1F5			
AT1G33760	Integrase-type DNA-binding superfamily protein	1.33760.1I99					
AT1G50680	AP2/B3 transcription factor family protein	1.50680.1B99	1.50680.1D99	1.50680.1H99			
AT1G73100	SDG19_SUVH3__SU(VAR)3-9 homolog 3	1.73100.1C99	1.73100.1E99	1.73100.1H99			
AT2G23340	DEAR3__DREB and EAR motif protein 3	2.23340.1A3	2.23340.1E2	2.23340.1F2			
AT2G30250	ATWRKY25_WRKY25__WRKY DNA-binding protein 25	2.30250.1C5	2.30250.1D2	2.30250.1F2			
AT2G43010	AtPIF4_PIF4_SRL2__phytochrome interacting factor 4	2.43010.1B99	2.43010.1C99	2.43010.1H99			
AT2G45050	GATA2__GATA transcription factor 2	2.45050.1A6	2.45050.1D6	2.45050.1G3	2.45050.1I5		
AT2G46830	AtCCA1_CCA1__circadian clock associated 1	2.46830.2D1	2.46830.2E1	2.46830.2F4			
AT2G47810	NF-YB5__nuclear factor Y, subunit B5	2.47810.1A2	2.47810.1B4	2.47810.1C1			
AT3G03750	SDG20_SUVR3__SET domain protein 20	3.03750.1B99	3.03750.1C99	3.03750.1E99	3.03750.1H99		
AT4G09460	AtMYB6_MYB6__myb domain protein 6	4.09460.1A1	4.09460.1D1	4.09460.1G1			
AT4G14540	NF-YB3__nuclear factor Y, subunit B3	4.14540.1A99	4.14540.1C99	4.14540.1E99	4.14540.1I99		
AT4G16610	C2H2-like zinc finger protein	4.16610.1A4	4.16610.1E6	4.16610.1G6	4.16610.1I2		
AT4G37260	ATMYB73_MYB73__myb domain protein 73	4.37260.1A99	4.37260.1B99	4.37260.1D99	4.37260.1E99	4.37260.1G99	
AT5G26580	AGL34__AGAMOUS-like-34	5.26580.1C4	5.26580.1D2	5.26580.1G3	5.26580.1H2		
AT5G60200	TMO6__TARGET OF MONOPTEROS 6	5.60200.1A99	5.60200.1E99	5.60200.1G99	Falta el tubo del G99		
AT1G03800	ATERF10_ERF10__ERF domain protein 10	1.03800.1A2	1.03800.1E5				
AT1G08970	HAP5C_NF-YC9__nuclear factor Y, subunit C9	1.08970.2D5	1.08970.2E3	1.08970.2I8			
AT1G09030	NF-YB4__nuclear factor Y, subunit B4	1.09030.1A1	1.09030.1C1	1.09030.1F99			
AT1G51120	AP2/B3 transcription factor family protein	1.51120.1F2					

AT1G75388	CPuORF5__conserved peptide upstream open reading frame 5	1.75388.1A2	1.75388.1D3	1.75388.1H3			
AT1G75490	Integrase-type DNA-binding superfamily protein	1.75490.1B4					
AT1G77200	Integrase-type DNA-binding superfamily protein	1.77200.1C99	1.77200.1H99				
AT2G34440	AGL29__AGAMOUS-like 29	2.34440.1A2	2.34440.1B1	2.34440.1C1			
AT2G36890	ATMYB38_BIT1_MYB38_RAX2__Duplicated homeodomain-like superfamily protein	2.36890.1G2					
AT2G40950	BZIP17__Basic-leucine zipper (bZIP) transcription factor family protein	2.40950.1A2	2.40950.1F4	2.40950.1H3			
AT3G13890	ATMYB26_MS35_MYB26__myb domain protein 26	3.13890.1E2	3.13890.1G1				
AT3G16500	IAA26_PAP1__phytochrome-associated protein 1	3.16500.1A6					
AT3G28470	ATMYB35_TDF1__Duplicated homeodomain-like superfamily protein	3.28470.1B99	3.28470.1C99				
AT4G31420	Zinc finger protein 622	4.31420.1E4	4.31420.1G2	4.31420.1H1			
AT5G06500	AGL96__AGAMOUS-like 96	5.06500.1B99	5.06500.1G99				
AT5G23090	NF-YB13__nuclear factor Y, subunit B13	5.23090.1D4	5.23090.1F6	5.23090.1G4			
AT5G58890	AGL82__AGAMOUS-like 82	5.58890.1A8	5.58890.1D10	5.58890.1F6			

Supplementary Table S5. Prediction of S-nitrosylation and nitration sites of potential NO targets

			GPS SNO & YNO2		iNitro -Tyr	iSNO-PseAAC
Protein	Position	Peptide	Score	Cutoff	Δ	
P49333, ETR1	4	****MEVCNCIEPQW	4.142	1.484		
	65	LVQFGAFIVLCGATHLINLWT				(+)
	99	VLTAVVSCATALMLV	3.549	2.443		
	573	WIESDGLGKGCTAIFDVKLGI				(+)
	634	VTKGLLVHLGCEVTTVSSNEE				(+)
	661	KVVFMDVCMPGVENY	2.832	2.443		
	195	GLELQLSYTLRHQHP			0.556	
	735	LLEPRVLYEGM****	2.766	2.51		
Q9S814, EIN2	344	RILAVAPALYCVWTSGADGIY				(+)
	370	TQVLVAMMLPCSVIPLFRIAS				(+)
	729	SFGKDISSGYCMSPTAKGMDS				(+)
	**1063**	**LLQSFRHCILKLIKL**	**3.505**	**2.443**		
	**1063**	**EAKLLQSFRHCILKLIKLEGS**				**(+)**
	1141	ADLIVSFGVWCIHRVLDLSLM				(+)
	**1218**	**AKPAKGKCTTAVTLL**	**2.897**	**2.443**		
	**1218**	**PPAAKPAKGKCTTAVTLLDLI**				**(+)**
	32	LLVSVGYIDPGKWV			0.072	
	525	TSVTSSVYDLPENIL	1.116	0.725		
	746	SQMTSSLYDSLKQQR	0.946	0.725		
	**783**	**RMQMLGAYGNTTNNN**	**1.264**	**0.828**		
	**783**	**RMQMLGAYGNTTNNN**			**0.548**	
	828	HGYQMKSYVDNLAKE	1.674	1.065		
	859				0.012	
	901	RQSERSYYGVPSSGN	0.757	0.725		
	984	RVGVPSTYDDISQSR	1.114	0.828		
	1099				0.587	
	1161				0.542	
B9DFU2, MAX1	467	FIPFGIGPRACVGQRFALQEI				(+)
	147				0.066	
	316	KNIFTSDYISAVTYE	1.14	1.065		
	341				0.291	
	409	KEVEIGGYLLPKGTW	1.4	1.065		
	504				0.322	
Q9SIM9, MAX2	**63**	**DLSLVPDCFRSISHL**	**2.511**	**2.443**		
	**63**	**NARDLSLVPDCFRSISHLDLS**				**(+)**
	215	SSEIVSITKSCPNLKTFRVAC				(+)
	317	GVALEALNSKCKKLRVLKLGQ				(+)
	332	VLKLGQFQGVCSATEWRRLDG				(+)
	346	EWRRLDGVALCGGLQSLSIKN				(+)
	372	DMGLVAIGRGCCKLTTFEIQG				(+)
	373	MGLVAIGRGCCKLTTFEIQGC				(+)
	410	KTLTDVRISCCKNLDTAASLK				(+)
	426	AASLKAIEPICDRIKRLHIDC				(+)
	471	DDGYERSQKRCKYSFEEEHCS				(+)
	536	EEIRIKIEGDCRGKRRPAEPE				(+)
	551	RPAEPEFGLSCLALYPKLSKM				(+)
	565	YPKLSKMQLDCGDTIGFALTA				(+)

	111	FVESLNVYTRSPSSL	0.865	0.828		
	169				1.363	
	464	HEEEDDGYERSQKRC	1.189	0.725		
	500				0.415	
Q8VY26, MAX4	-	-	-	-		
	79	EKEKVEGERRCHVAWTSVQQE				(+)
	166	YKAAKKHNRLCYREFSETPKS				(+)
	217	VIKLGDGRVMCLTETQKGSIL				(+)
	395	GKATVIIADCCEHNADTRILD				(+)
	474	LGQKYRYVYACGAQRPCNFPN				(+)
	480	YVYACGAQRPCNFPNALSKVD				(+)
	565	PYGLPYGLHGCWIPKDXXXXX				(+)
	167				0.461	
	313				0.342	
	368				1.265	
	463				1.023	
	566				0.060	
Q8S8E3, PYL6	61	HVVGPSQCFSVVVQD	4.196	2.443		
	96	PQAYKHFVKSCHVVIGDGREV				(+)
	199	TCSFADTIVRCNLQSLAKLAE				(+)
	89				0.187	
	**176**	**RTRVVESYVVDVPAG**	**1.4**	**1.065**		
	**176**				**0.739**	
Q1ECF1, PYL7	36	HHCRENQCTSVLVKY	3.549	2.443		
	170	TCYFVESLIKCNLKSLACVSE				(+)
	177	LIKCNLKSLACVSERLAAQDI				(+)
	195	QDITNSIATFCNASNGYREKN				(+)
	14	DDTDTEMYGALVTAQ	1.405	0.725		
	162				0.527	
Q9LN63, BES1/BZR2	62	AQGNYNLPKHCDNNEVLKALC				(+)
	-	-	-	-		
	-	-			-	
